# Determining Phase Separation Dynamics with an Automated Image Processing Algorithm

**DOI:** 10.1021/acs.oprd.2c00357

**Published:** 2023-03-14

**Authors:** James Daglish, A. John Blacker, Gregory de Boer, Alex Crampton, David R. J. Hose, Anna R. Parsons, Nikil Kapur

**Affiliations:** † School of Mechanical Engineering, University of Leeds, Leeds LS2 9JT, U.K.; ‡ School of Chemistry, University of Leeds, Leeds LS2 9JT, U.K.; § Chemical Development, Pharmaceutical Technology and Development, Operations, AstraZeneca, Macclesfield SK10 2NA, U.K.

**Keywords:** separation science, image analyses, emulsion, liquid−liquid system

## Abstract

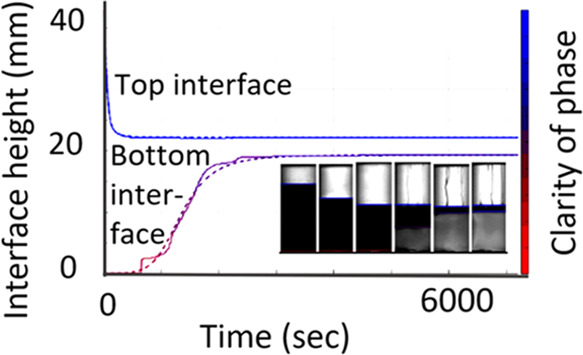

The problems of extracting products efficiently from reaction workups are often overlooked. Issues such as emulsions and rag layer formation can cause long separation times and slow production, thus resulting in manufacturing inefficiencies. To better understand science within this area and to support process development, an image processing methodology has been developed that can automatically track the interface between liquid–liquid phases and provide a quantitative measure of the separation rate of two immiscible liquids. The algorithm is automated and has been successfully applied to 29 cases. Its robustness has been demonstrated with a variety of different liquid mixtures that exhibit a wide range of separation behavior—making such an algorithm suited to high-throughput experimentation. The information gathered from applying the algorithm shows how issues resulting from poor separations can be detected early in process development.

## Introduction

High-throughput experimentation (HTE) enables many experiments to be investigated in parallel and on a small scale by utilizing automated technology and statistical design of experiments. It requires less human intervention than conventional lab-scale experimentation resulting in higher precision and better repeatability. HTE is used in the pharmaceutical industry to screen for possible drug candidates and to optimize reactions and crystallizations during early process development within a reduced time frame.^1,2^ However, it is far less frequently used for the optimization of intermediate work-up steps, possibly due to the need to procure specialist equipment and adopt specialized procedures.^3−5^ Within workups, HTE is most commonly used to optimize the final crystallization process as a “catch-all” purification technique.^5−7^ Liquid–liquid extraction is used routinely as a post-reaction work-up to separate byproducts, excess reagents, and other impurities.^8,9^ However, its optimization is often overlooked or is quite rudimentary. If done incorrectly, the work-up can cause downstream process or product inconsistencies or impact upon subsequent crystallization. A requirement of HTE is the design of robust algorithms to extract data from physical systems, which is the focus of this work.

In the field of extraction science, Selekman et al. demonstrated a high-throughput extraction workflow and used this to identify optimum conditions for the removal of a genotoxic impurity and a residual amine base from a process stream through liquid–liquid extraction.^5^ The separation of the two phases (settling time, emulsion formation, and phase split quality) was considered within the workflow, as these can affect the feasibility of process scale-up and production cost. Emulsion or rag layer formation and phase split quality were observed only qualitatively between samples, and phase separation time was compared visually over a timescale of minutes. Duffield et al. used a high-throughput methodology to extract phase volumes of a liquid–liquid system in equilibrium.^10^ After cropping the images and selecting the color channel giving the greatest contrast between the phases, the gradient of the intensity was calculated, and where this passed a threshold, an interface was located. This allowed partition coefficients of a third soluble phase to be established. Barrington et al. used an imaging approach to establish changes in contrast across an image, and from this infer process diagnostics.^11^ While it does not address liquid–liquid separations, it does capture dynamic data.

The dynamic phase separation behavior of liquid–liquid systems is complex and depends upon the physicochemical properties of the system. The controlling properties of the liquid include density, viscosity, and interfacial tension together with the relative phase volume. Process variables include mixing energy and vessel geometry with the link between the two being the formation of a dispersed phase of droplets suspended within the continuous phase.^12^ Taken together, these variables influence the droplet size with the interfacial properties playing an important role in the subsequent behavior. Mechanisms of liquid–liquid separation are sedimentation, creaming, flocculation, coalescence, and Ostwald ripening.^13^

**Figure 1:**
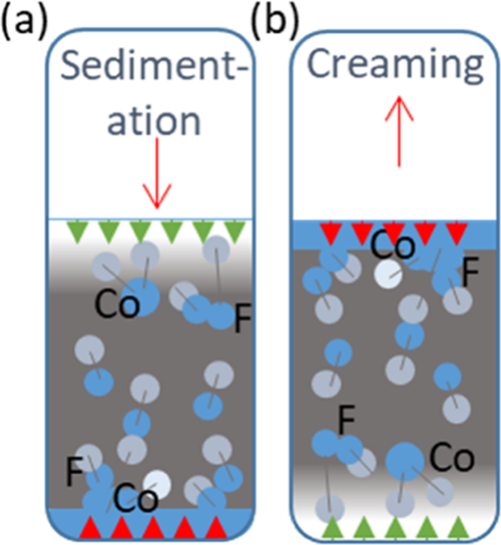
Schematic showing (a) sedimentation, where droplets are of a higher density than the continuous phase and (b) creaming where droplets are of a lower density than the surrounding phase. Light gray droplets are starting points and dark blue drops after some elapsed time, lines show tracks. Co are coalescing events, and F are flocculating events. Green arrows indicate the gradually emerging band clear from droplets due to gravity separation, and red arrows the emerging band due to coalescence of the droplet phase.

Sedimentation is where the droplets are denser than the continuous phase, and creaming is where the droplets are lighter than the continuous phase (Figure 1). At the simplest level, the movement of a single droplet can be captured by Stokes law (eq 1), which depends on the difference in density between the two phases, ρ_d_ – ρ_c_, continuous phase viscosity, μ_c_, acceleration due to gravity, *g*, and droplet diameter, *d*.
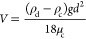
1Coalescence refers to where two or more droplets merge to form a fluid continuum, whereas flocculation is where two droplets aggregate but do not merge together (Figure 1). The coalescence and flocculation of droplets during separation can hinder the rate of settling,^14,15^ for example, by restricting the expulsion of the continuous phase. The interfacial properties of the two phases are important due to the requirement for film drainage of the continuous phase between the two droplets before coalescence can take place.^16^ The rate of separation in liquid–liquid systems can be severely limited if the dispersed phase droplets are stabilized within the continuous phase such that emulsions or rag layers form. Small particulates can generate Pickering emulsions and surface-active molecules can form stable barriers that resist coalescence.^17^ Surface-active molecules may also reduce interfacial tension, resulting in smaller droplets and longer separation times. Moreover, small changes in salinity, pH, temperature, or phase composition can drastically change how compounds interact at the liquid–liquid interface and subsequently the rate of separation in systems that include surface-active molecules.^18−24^ Within this work, samples with systematically varying separation properties have been established. The samples can be viewed as ones in which each formulation has different settling properties. The HLD method has been used to support the development of this set as it captures the influence of salt concentration, solvent, and temperature on surfactant systems and the type of emulsion.^18,25,26^ A brief explanation of the HLD method is included in Section 6.0 with a more comprehensive explanation found in ref (27).

A number of techniques have been developed to characterize emulsion stability.^13,28^ Light scattering techniques are commonly used for lab-scale investigations and can predict droplet size distributions and sedimentation rates.^29−31^ However, these methods are sensitive to small differences in refractive index or large differences in dispersed phase concentrations, which can lead to errors. Ultrasound and magnetic resonance imaging has been used to provide droplet size distributions and volume fraction information.^32,33^ These techniques are well suited to highly stable emulsions that can be monitored over a long period of time.

“Bottle tests” and visual observations can be used as a fast and simple method to determine creaming/sedimentation rates during separation. BS2000-412:1996 and ISO 6614:1994 describe this method with standardized mixing regimes for qualitative comparision.^34^ Imaging techniques have been used to augment this method and provide a quantitative measure of sedimentation/creaming rates. Novales et al. plotted the grayscale intensity value against height for different emulsions over time.^35^ Wang et al. integrated the grayscale intensity data with respect to height, which gave a series of curves that corresponded to the clarity of emulsions and amount of phase separation that had occurred.^36^ Ghanbari et al. measured the absorbance of light by an emulsion sample and compared this to a clear sample, resulting in a “light absorbance index.”^37^ Image analysis techniques are suited to investigating the phase separation behavior of relatively fast settling systems as information about the entire height of the sample can be obtained in one instance and images can be collected at a high frequency. However, image analysis has not yet been demonstrated to explicitly track interface behavior in dynamically settling systems.

Edge detection image analysis techniques are ideally suited to determining phase heights over time. Edge detection is a method to find edges and boundaries between objects within an image based on differences in pixel brightness. There are three steps to edge detection algorithms (i) filtering—to reduce noise in the image which would produce false edges; (ii) enhancement—to emphasize pixels where there is a significant change in local intensity values, frequently through computing the gradient of the pixel intensities; (iii) detection—to find the location of the edge via thresholds applied to the gradient function or finding the zero-crossing point of the second derivative. Some commonly applied first-order algorithms are the Canny,^38^ Roberts,^39^ Sobel,^40^ and Prewitt^41^ methods. A common second-order algorithm is the Marr and Hildreth or Laplacian of Gaussian method (LoG).^42^ One of the challenges in edge detection is balancing necessary noise reduction while not over-smoothing edges and losing detail.^43^ Determining the direction of an edge and creating a function that represents the gradient creates significant challenges in many applications, but fortunately for emulsion separations, we are only interested in edge detection as a one-dimensional problem (height within a vessel) and therefore much of the complexity involved in image analysis can be reduced and an algorithm based on the first and second derivatives of the image grayscale data in the vertical direction (detailed in the Materials and Methods section).

Presented here is a robust and flexible image processing algorithm that can detect the height of the interface between two liquid phases over time for both fast and slow settling systems. The nature of the dispersed phase, whether an aqueous droplet phase suspended in a continuous organic phase (W/O meaning water in oil) or an organic droplet phase dispersed in a continuous aqueous (O/W meaning oil in water) has been deduced from image data and confirmed with conductivity readings. The algorithm has been designed to give results for a multitude of different systems with minimal human intervention or changes to the algorithm inputs with a view for further development and integration into high-throughput experimentations of extraction processes. Several liquid–liquid systems were studied; three different liquid biphasic solutions were analyzed at three different phase ratios: toluene–deionized water (pH 7); toluene–acetate buffer (pH 4); toluene–glycine buffer (pH 10). Two surfactant solutions (0.01 and 0.1 M SDBS) at 10 different salt concentrations were also analyzed. By varying the salt concentration systematically, a range of very slow through to fast settling interfaces, O/W and W/O emulsions, and distinct and indistinct interfaces were produced. A selection of the liquid–liquid systems studied at small scale has been scaled up to 20 L to demonstrate how variations in separation characteristics captured by the algorithm correspond to the behavior at a larger scale.

## Results and Discussion

**Figure 2:**
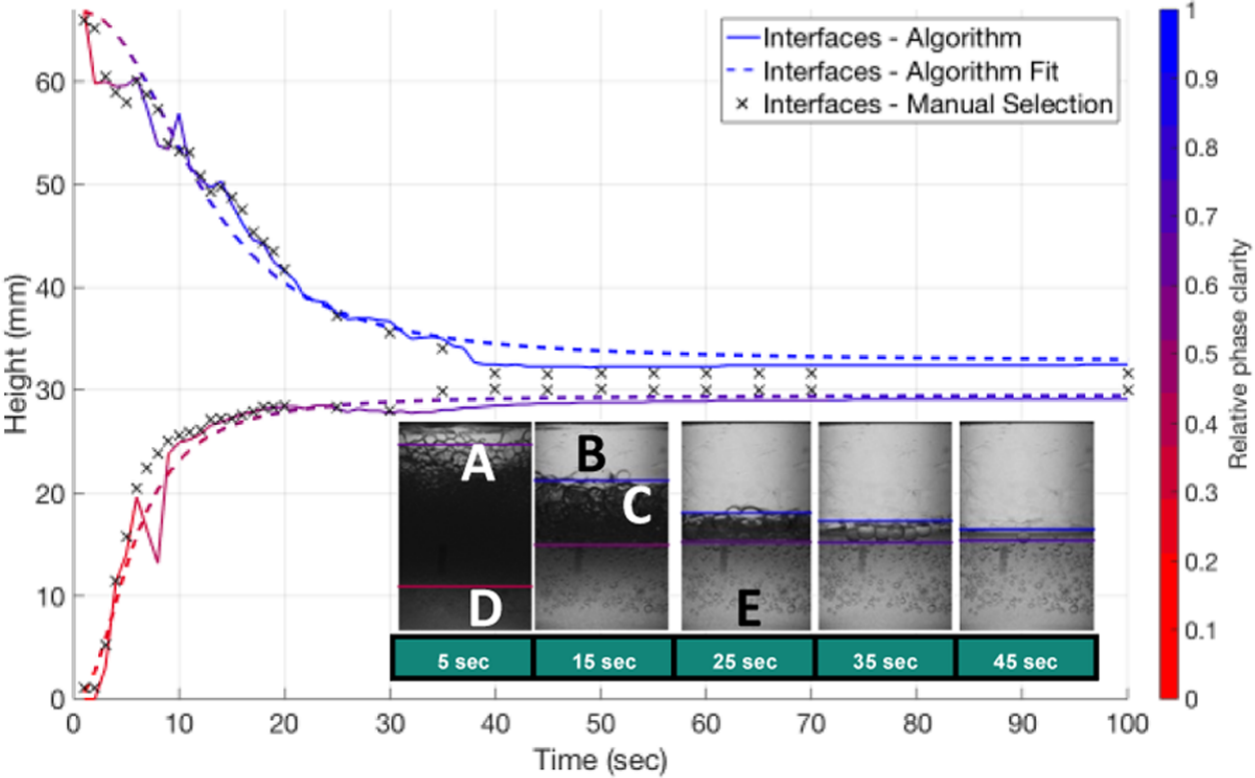
Detected interfaces and relative phase clarity over time for repeat 1 of the toluene–water time series at a phase ratio of 1 compared with values for the interface location found manually.

In total, 29 separation experiments were carried out, each with a different formulation/phase ratio. Before discussing the results in detail, a typical separation pattern is described. Figure 2 (inset images) shows a typical separation at five time points. Upon initial mixing, the vial is black due to the multiple refractions at each droplet surface. In this example, a clear layer at the top of the emulsion starts to develop after 5 s (region A in Figure 2; also shown in Figure 1b, red arrows) as the suspended droplets rise and coalesce, indicating an O/W emulsion. A sharp interface is observed as coalesced oil droplets form this band (region B). There is still a dark region below this clear layer (region C) where uncoalesced droplets produce multiple refractions. Below the emulsion layer, a second clear region is observed (region D). Since the rising droplets in this area span a range of sizes, the interface delimiting the clear continuous phase is less distinct, particularly for rapidly separating systems (compare Figure 2 to Figure 4), but over time, this region becomes more transparent (region E). Oil droplets can be seen to cling to the vessel wall in this region which further reduces clarity. Below the lower interface and above the upper interface are the separated phases, and between these is the separating emulsion. Figure 2 shows the height of the two settling interfaces over time determined by the algorithm and by eye. The phase clarity relative to a sample of pure toluene for the top section and pure water for the bottom section is provided by a color map.

### Separation Behavior of Toluene and Aqueous Solutions

**Figure 3:**
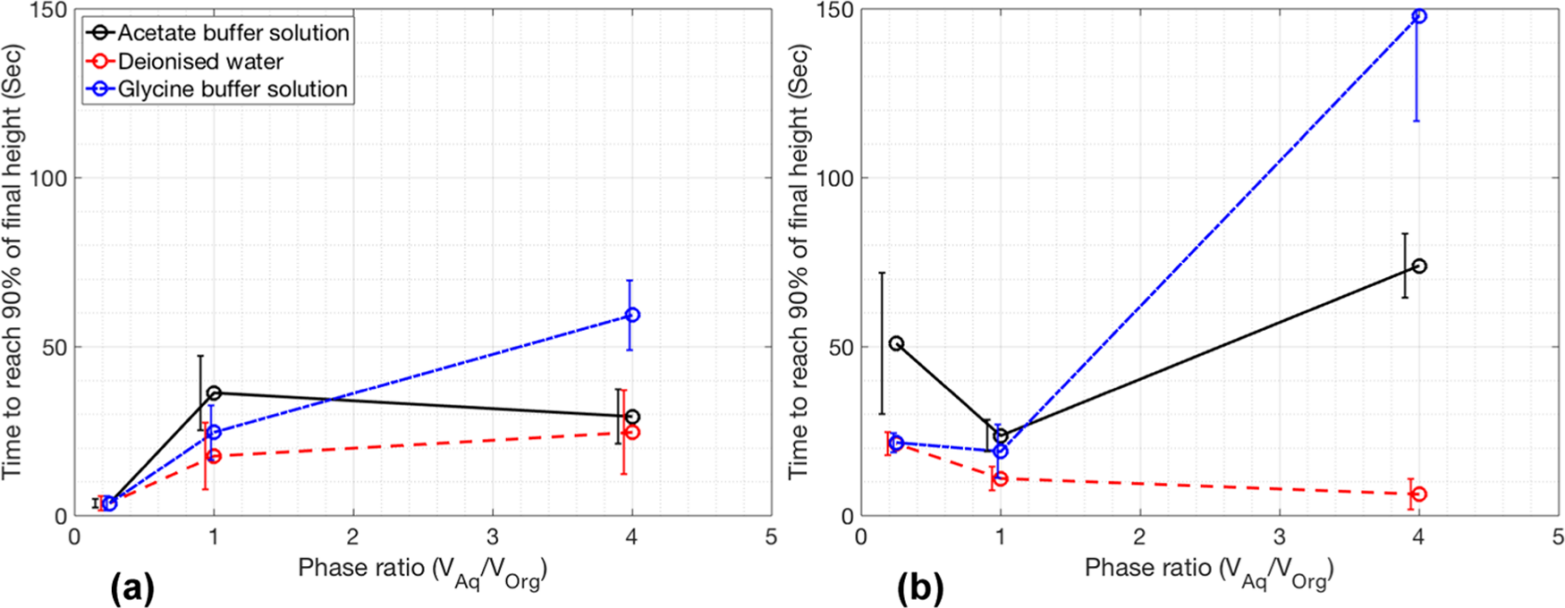
Time taken for (a) top and (b) bottom interface of each solution to reach 90% of its final value at three different phase ratios.

Nine liquid combinations were analyzed using the algorithm. The three liquid biphasic solutions used were toluene–deionized water (pH 7); toluene–acetate buffer (pH 4); and toluene–glycine buffer (pH 10). These were prepared in three phase ratios (*V*_Aq_/*V*_Org_) of 0.25, 1, and 4. From the collected images, it could be deduced that all of the cases at phase ratios 4 and 1 were O/W emulsions, while the 0.25 phase ratio cases were W/O. For each of the nine cases (with three repeats per experiment—data in the Supporting Information), the average time at which each interface reached 90% of its final height is shown in Figure 3.

All systems at a phase ratio of 0.25 are such that a W/O is formed with separation by sedimentation (aqueous droplets sedimenting in a less dense organic phase—Figure 1a). In all cases, the upper interface reached its equilibrium quickly (<10 s), suggesting the droplets are relatively large in size (eq 1) and sediment quickly. The emergence of a clear coalesced band at the bottom of the container is slower, possibly as a result of charge or due to the nature of the dynamics of the film of oil that must be excluded between the droplets^44^ before coalescence.

At a phase ratio of 4, all systems are an O/W emulsion and separation is by creaming (oil droplets rising in a more dense aqueous phase—Figure 1b). The top interface, which is reached in 25–50 s is a result of the rising droplets which then coalesce to form a clear band. The lower interface for the buffer solutions takes considerably longer (up to 150 s). The gradual rise of this interface (Figures S82–S91) suggests that there is a size fraction of smaller droplets within the emulsion. The rate of coalescence may have been reduced in the buffer solutions because of the nature of the charge between the two phases (which varies with ionic strength and pH). An increased charge may have increased repulsion between droplets and in turn reduced the frequency of collisions, giving a reduction in the number of coalescing droplets and a slower separation.^22,24^ It has also been found that sodium acetate salts can reduce the surface tension of aqueous–organic mixtures, which would result in the formation of smaller dispersed droplets.^21^

### Separation Behavior of Toluene and Sodium Dodecylbenzenesulfonate (SDBS) Surfactant Solutions

To systematically demonstrate the performance of the imaging algorithm, a system with settling performance across a range of times was identified using a constant toluene/water/surfactant mixture with the single parameter of the salinity of the aqueous phase used to drive the nature of the emulsion. A series of surfactant-stabilized liquid bi-phases were produced by increasing the NaCl concentration to raise the HLD value of the system from ca. −3 through to 1, passing close to the 0 point; see Table 1. Close to HLD = 0, the lowest interfacial tension arises and consequently gives rapid separation. At this point, and dependent on the concentration of surfactant, a more complex emulsion can exist where both phases are continuous with a complex structure of interconnectedness of the aqueous phase through the organic phase.^45^ A positive HLD value corresponds to a W/O emulsion, and a negative HLD value results in an O/W emulsion. The time required for the emulsion phases to separate increases exponentially as the HLD value deviates from 0. Only when a system is situated far from the phase inversion point does the time for separation tend to reduce again, due to increased interfacial tension, larger droplet sizes, and less efficient surfactant interfacial packing. Twenty emulsions were formed with two different surfactant concentrations and 10 salt concentrations. Both very slow and fast settling interfaces, O/W and W/O emulsions, and clear and unclear interfaces were observed during the experiment. The 10 salt concentrations shifted the equilibrium of the surfactant system from strongly negative on the HLD scale (O/W) through to positive (W/O) passing through a region of mixed O/W and W/O. A third microemulsion phase was not observed during these experiments, possibly due to the low surfactant concentration or limited surfactant solubility in toluene. Nevertheless, a significant decrease in emulsion stability was recorded near to HLD = 0.

**Table 1:** NaCl Concentration (mg/mL) in the Aqueous Phase of Each Vial and Its Corresponding HLD Value

vial no.	NaCl concentration (mg/mL) in 0.01 M SDBS solution	HLD value (0.01 M solution)
1	0.45	–3.38
2	10.76	–0.91
3	16.63	–0.49
4	21.72	–0.23
5	27.68	0.006
6	33.85	0.20
7	43.39	0.45
8	57.13	0.72
9	66.48	0.87
10	78.6	1.04
11	0.41	–2.99
12	10.76	–0.87
13	15.91	–0.50
14	22.21	–0.19
15	26.8	0.007
16	32.87	0.19
17	43.48	0.46
18	56.59	0.72
19	66.51	0.88
20	77.93	1.04

**Figure 4:**
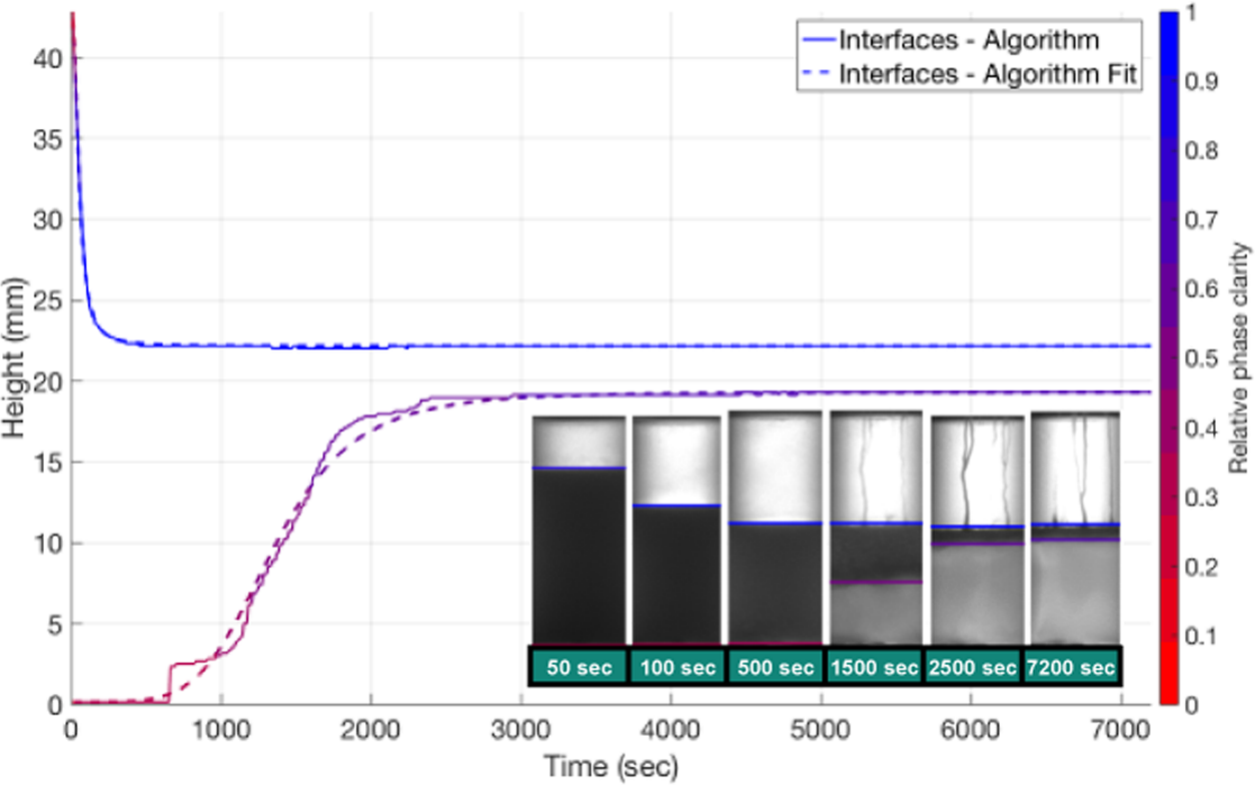
Detected interfaces and normalized grayscale intensity over time for the 0.01 M solution with 27.68 mg/mL NaCl (HLD = 0.006).

An example interface location graph from vial 5 (HLD ∼ 0) has been presented in Figure 4. The top interface is more distinct than the bottom, so was detected earlier than the other interface by the algorithm. Sample conductivity was measured post experiment by remixing the samples and quickly placing a conductivity probe inside the vial once mixing had ceased. The conductance of this sample was in the 10 μS/cm range which, considering the overall salt concentration of the sample, suggests it was mostly a W/O emulsion but with some O/W. The sample lies within the transitional region of the HLD scale meaning the emulsion was expected to be partly O/W and partly W/O. However, the cloudy bottom phase suggests that fine toluene droplets are present in the water, which would mean the emulsion is mostly O/W. It is inconclusive if this sample is mostly O/W or W/O, but as the sample lies within the transitional region of HLD space, this is to be expected. The interface detected by the algorithm closely matches the interface locations shown in the inset images of Figure 4, and the sigmoidal curve demonstrated a good fit to this data with *R*^2^ values of 0.997 and 0.995 for the two interfaces.

**Figure 5:**
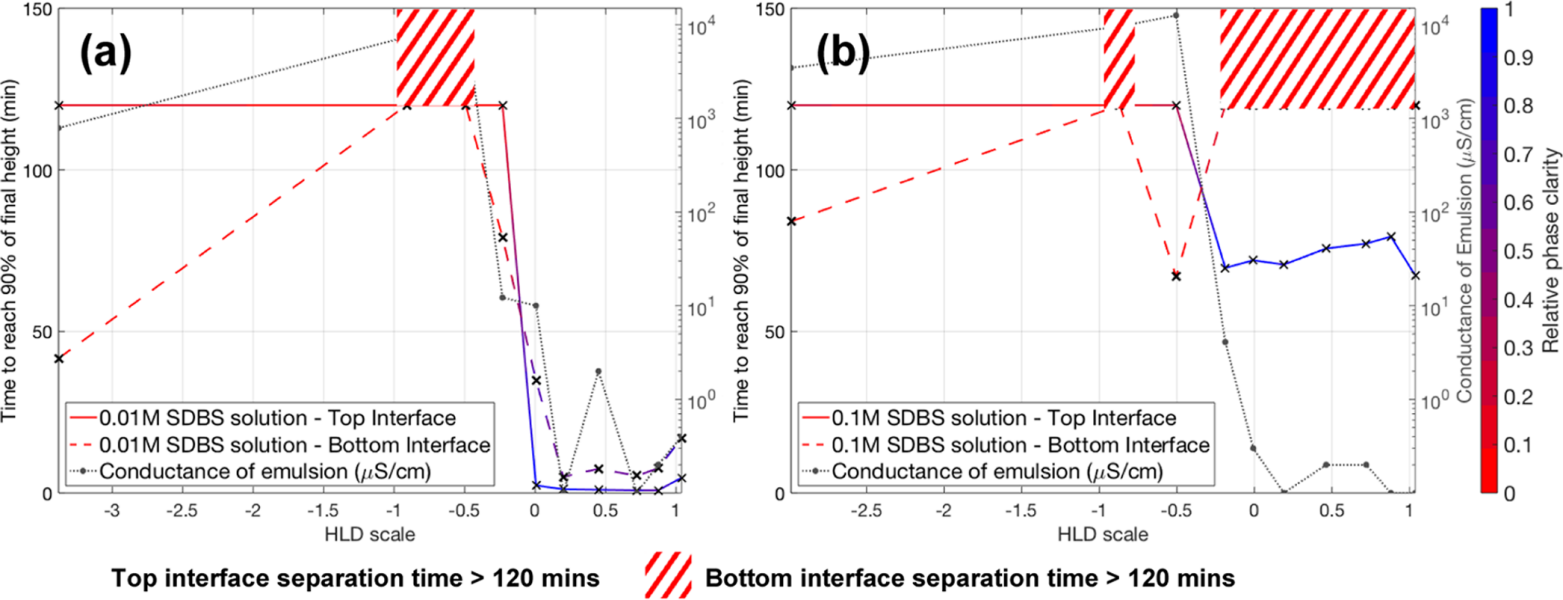
Time taken for (a) 0.01 M SDBS and (b) 0.1 M SDBS solution top and bottom interfaces to reach 90% of their final height and the recorded conductance of each emulsion. The relative phase clarity above and below the interface at the final timestep is shown. The shaded blocks are located at the points where separation of the top or bottom interface took longer than 120 min to separate.

Figure 5a shows the time for both the top and bottom interfaces of the 0.01 M emulsions to separate depending on its location in the HLD scale. For the lower surfactant concentration, the emulsion was observed to be stable at HLD <−0.5, with long settling times for both interfaces (for this process-related study, settling times were truncated at >120 min). The measurement of conductivity indicated that these are O/W emulsions. Increasing salt concentration shifts the surfactant equilibrium toward the organic phase, the system becomes less stable, and the emulsion separates rapidly. At the point of HLD = 0, the emulsion enters a transitional phase and is a mixture of W/O and O/W. This transitional phase appears to span a range of HLD values from 0 up to 0.87 (vials 5–9). Both phases in vials 7, 8, and 9 remain opaque after the bulk of separation has occurred, suggesting fine droplets of water are present in the continuous toluene phase and toluene droplets in the continuous water phase (see Section 4.0). At HLD = 1, a more stable W/O emulsion forms, as suggested by the increased clarity of the bottom interface, and increased separation time of both interfaces (see Section 4.0). Theoretically, if more salt were added, the W/O emulsion would become more stable before reaching a plateau, similar to the highly negative HLD samples. However, the relationship between the HLD scale and salinity is logarithmic, so additional salt has a diminishing effect on the HLD value.

For the 0.1 M SDBS surfactant solutions, Figure 5b shows that an increase in surfactant concentration resulted in the expected increase in emulsion stability. Any interface that did not settle out within 120 min is shown with a shaded block. Within the recording time investigated, the top interface can be seen to follow a similar trend to that of the 0.01 M samples. As before, as the HLD value approaches 0, the emulsion becomes less stable. This reduction in separation time coincides with a reduction in conductivity, suggesting that the emulsion is transitioning from O/W to W/O. It is clear from Figure 4a,b that the separation time and emulsion type can be modified by varying salt concentration in line with HLD theory even at low surfactant concentrations. The proposed imaging algorithm can determine the separation time of emulsion systems consistently across the HLD range.

### Predicting Separation Behavior Upon Scale-Up

Five cases from the previous experiments were scaled up to 20 L, to illustrate the challenges of using small-scale experimentation for better understanding processing conditions. In total, five systems were selected, covering a range of settling times: toluene–deionized water and toluene–glycine buffer solutions at a phase ratio of 4 due to a large difference in settling times and surfactant solutions with HLD values of −0.49, 0 and 0.45 covering settling times with an order of magnitude difference. A glass pilot scale vessel was selected, as shown in Figure S153. The transparency, often not available on larger vessels, allowed manual recording of the upper and lower interface position to be made.

**Figure 6:**
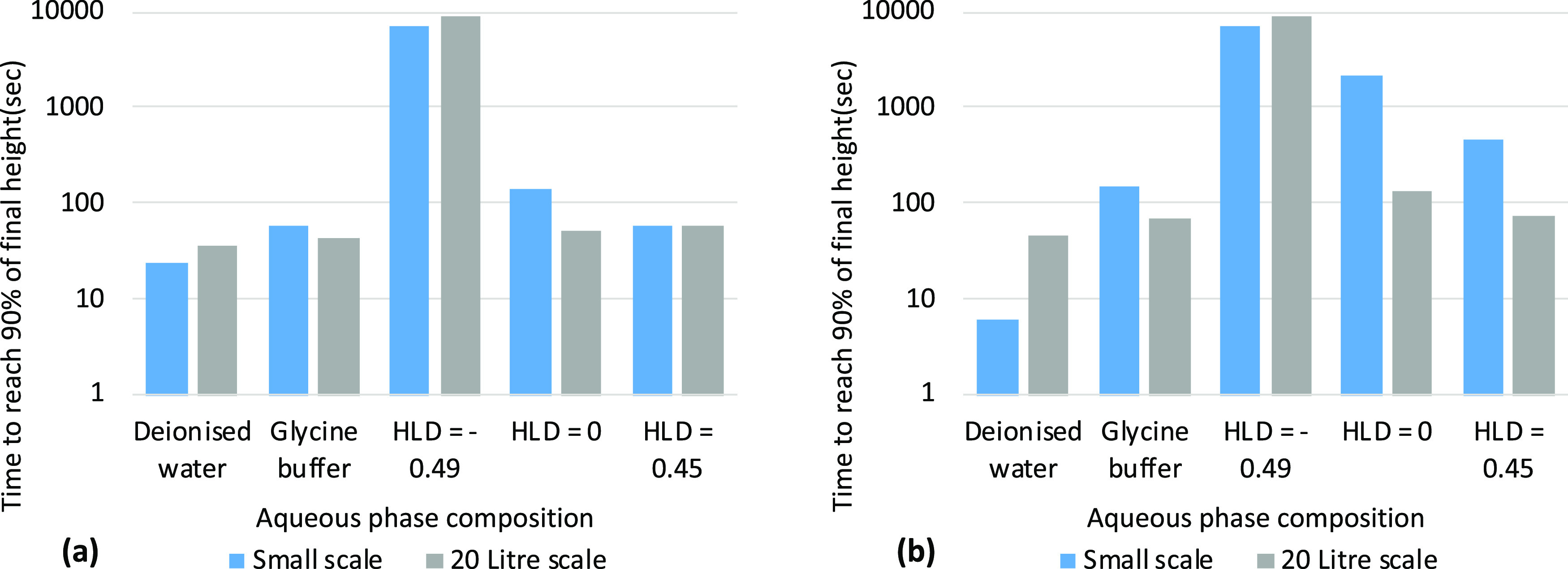
Time to reach 90% of the final height for (a) the top interface and (b) the bottom interface in the toluene–deionized water and glycine buffer solutions and three different HLD values −0.49, 0, 0.45 at small and 20 L scales.

Figure 6 shows the results of the study, with the time for the interface to reach 90% of its long-time settled value recorded. Are the small-scale lab tests really a good predictor of settling performance in the large vessel? The HLD = −0.49 case shows long settling times in both the vial tests and the pilot vessel. Under these conditions, a stable emulsion is formed with a settling time of ∼3 h. In all other cases except the deionized water case, the settling of phases within the vials takes longer than the vessel, but is of a similar order. Considering the geometry, the distance required for the droplets to travel to leave two separate layers is an order greater in the scale-up vessel compared to the vials and, because the settling time is a strong function of droplet diameter (eq 1), this suggests that the droplet size from the hand-agitated vials is considerably smaller than for the pilot vessel. The rotor within the vessel is a simple paddle, located toward the base of the vessel and without baffles present, which would give a low specific energy and form relatively coarse emulsions. Such a system will draw down the oil phase into the aqueous phase,^10^ promoting O/W emulsions for cases between HLD 0 and HLD 1.

Other adaptations of the shake test (e.g., vortex mixing, orbital mixers,^46^ servo-driven shake platforms—Section 5.0) will offer differing energy densities. The more controllable nature would allow for reporting under more standardized conditions, but all physical influences of the mixer on the resulting emulsion must be reported.

So, while the two scales correlate to one another and the vial-based test with the imaging algorithm does support a quick assessment of process scale-up, a good understanding of process changes such as mixing regime and energy input across the scales is still important, particularly where conditions can lead to either O/W or W/O emulsions depending on processing parameters.^47^

## Conclusions

The image processing algorithm has been shown to work well across a range of emulsion types. Both O/W and W/O mixtures have been examined with separation rates ranging from seconds through to hours and very subtle interfaces between the two phases have been detected. The algorithm dealt well with imperfect images where there was noise in the images due to droplets adhering to the glass walls. The algorithm has been used on volumes as small as 15 mL demonstrating applicability to HTE with application in both formulation science and (as is the motivation of this work) process separations in liquid–liquid systems. Further developing the experimental apparatus to include an automated shaker rack would increase the number of samples that could be analyzed at once—an initial prototype of such a shaker rig is shown in Section 5.0, but as shown in the study of scale-up, understanding the influence of process parameters on the emulsion type remains important.

The integration of this image processing algorithm into a work-up extraction analysis could provide vital and quantitative information on the feasibility of scale-up due to long or challenging separations and loss of product to rag layer formation. Furthermore, this work highlights that small changes in salinity or aqueous and organic phase characteristics can have a significant effect on separation rates and should be considered during initial process screening alongside conventional extraction efficiency studies.

## Materials and Methods

### Toluene and Aqueous Solution Experiments

**Figure 7:**
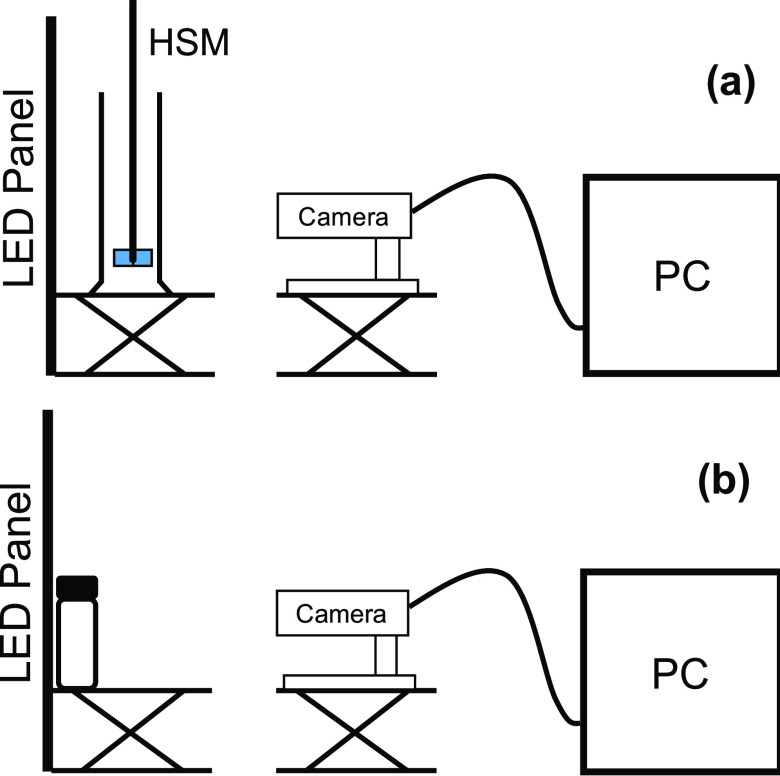
(a) Experimental setup with high shear mixer (HSM). (b) Experimental setup with hand-shaken test tubes.

All solvents and chemicals were purchased from Sigma-Aldrich, Inc. One liter of both 0.12 M, pH 4 acetate and 0.08 M, pH 10 glycine buffer solutions were made using standard methods. 200 mL of three biphasic solutions, toluene–deionized water, toluene–acetate buffer, and toluene–glycine buffer, were prepared in phase ratios (*V*_Aq_/*V*_Org_) of 0.25, 1, and 4. An illuminated LED panel was set behind a 1 L measuring cylinder containing the test solution. A Basler acA1300-30 μm area scan monochrome camera was set at a fixed distance from the center of the measuring cylinder and horizontally in line with the 100 mL marker as shown in Figure 7a. A high shear mixer (HSM) was lowered halfway into the liquid and mixed for 5 min at 1500 rpm. Immediately after mixing, the HSM was removed to avoid obstructing the camera view and images were taken in 1 s intervals for 10 min after mixing ceased and the images were stored for later analysis. All experiments were conducted at room temperature.

### Toluene and Surfactant Solution Experiments

15 mL vials were filled with equal volumes of toluene and 0.01 or 0.1 M solutions of sodium dodecyl benzene sulfonate (SDBS). Sodium chloride was added to each vial in aqueous phase concentrations ranging from 0.05% (w/v) to 7.8% (w/v) to create the range of HLD values found in Table 1. The vials were placed as in Figure 7b, and up to five were lined up in front of the LED screen to simultaneously measure separation rates. Each vial was hand-shaken for 2 min, left for 10 min, and then shaken again for 2 min before being placed in front of the LED screen for recording. Images were taken every 10 s. All experiments were conducted at room temperature. Each image was then later processed according to the algorithm presented below and in Section 1.0.

### Description of the Algorithm

**Figure 8:**
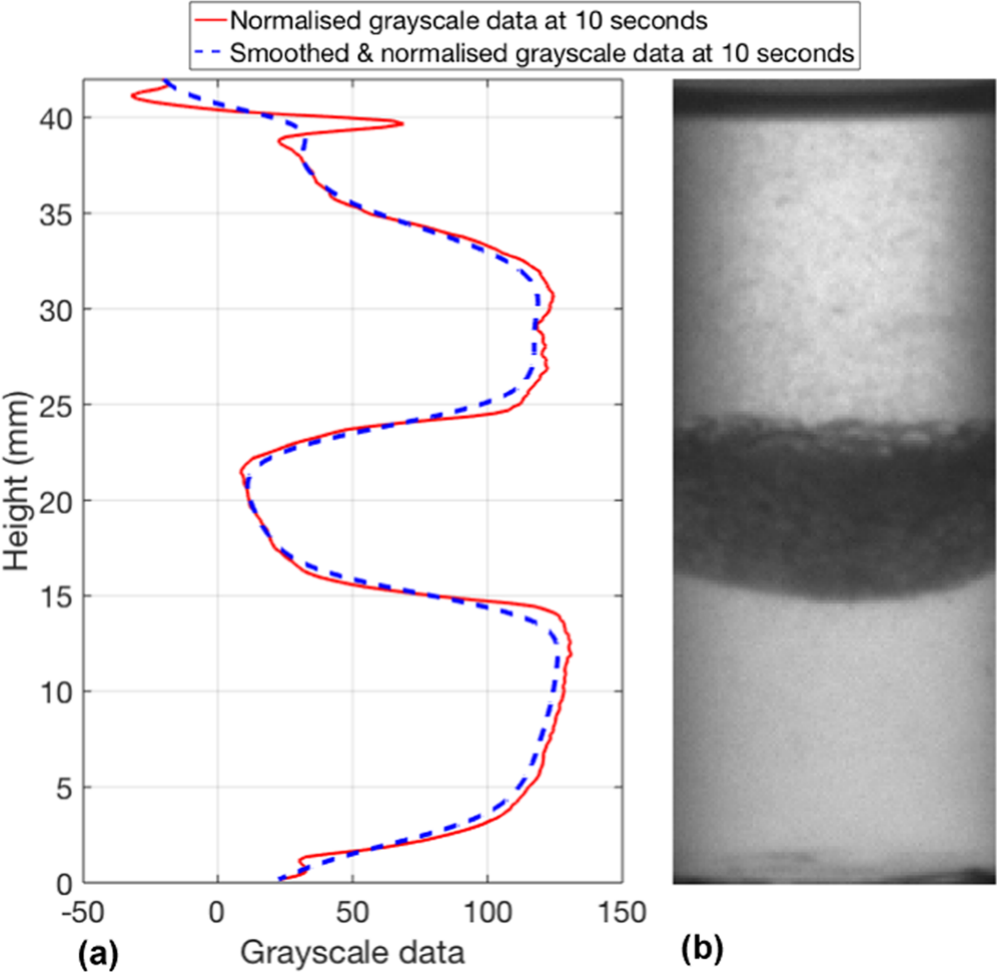
Normalized and smoothed grayscale data at 10 s (a). Image of toluene and deionized water separation after 10 s (b).

The 24 processing steps are broken down by the flowchart in Figure S1. The algorithmic procedure is described for an example image series recording the separation of toluene and water in a 15 mL vial. In step 1, the average grayscale intensity is calculated (255 = white pixel, 0 = black pixel) across the width of the liquid vessel at each pixel height and each timestep in the image series. The vial images were captured by 250 vertical pixels and 100 horizontal pixels, each pixel corresponding to 168 μm. Each grayscale measurement *I*_h,t_ was normalized by subtracting the grayscale measurement at the initial timestep, *I*_h,t_0__. Figure 8 shows the normalized grayscale intensity profile over the height of the liquid vessel at a single timestep (10 s) and the corresponding image. Once the normalized intensity profile for each image in the time series was obtained, a zero-phase low-pass finite impulse response (FIR) filter was applied in step 2. This smooths the normalized grayscale intensity data removing high-frequency noise and leaving only the low-frequency curve features. Details of the applied low-pass filter for each case are given in Section 2.1.

**Figure 9:**
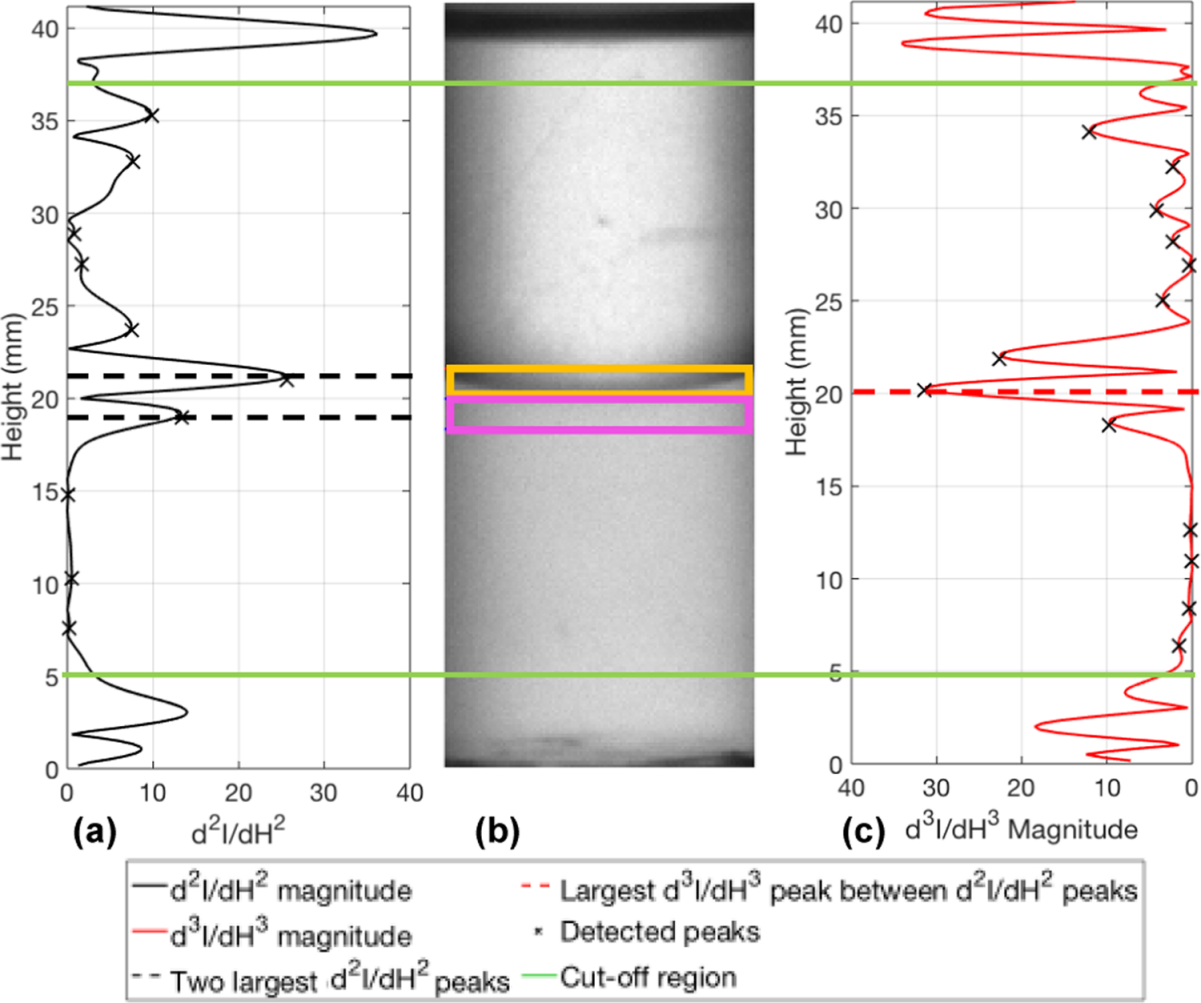
(a) Magnitude of second and (c) third derivatives at the final timestep and (b) the corresponding image of toluene and water. The orange and pink areas are the areas over which *I*_Ave_top__ and *I*_Ave_Bottom__ are calculated.

In step 3, the first, second, and third derivatives of the grayscale intensity data, with respect to height, are calculated and their magnitudes are found. All of the maximum and minimum peaks in , , and ,  are then found in step 4. These are stored for use in steps 5, 7, 11, 13, and 17. It is assumed that two settling fronts exist in each image series, a sedimentation front and a creaming front. If one or more of the fronts is not detected by the algorithm, then a stable emulsion has formed, which does not fully separate within the time frame of the image series. In step 5, the final image in the time series is analyzed to determine the location of the interface at the final recorded time. The algorithm is set to find the most “intense” peak in the final image. If the mixture has fully separated, then only one interface will exist but depending on the meniscus size, sample clarity, and light refraction through the sample, either the top or bottom edge of the interface will be found. If both sedimentation and creaming have occurred but there still exists a rag layer between the phases, then either the top or bottom of the rag layer will be found depending on which edge provides the most “intense” peak. If only sedimentation or only creaming occurs, then the interface will be found at the edge of the emulsion phase. If settling does not occur, then the algorithm will find the largest peak, due to noise in the data, but this will be noticeably smaller than if separation had occurred. To find the final interface location (*h*_set_), the top and bottom 30 pixels are cropped from the data set (in steps 5, 7, and 11 only). The number of pixels can be increased or decreased as needed depending on the image resolution and container height, but 30 pixels was sufficient for every case in this study. This is done so that the large second and third derivative peaks that occur at the liquid/air interface and container bottom are ignored. The cropped portion of the data is shown in Figure 9 by the green lines. The crosses marked on the two graphs show where a peak has been found. The final interface location is selected as the largest third derivative peak between the two largest second derivative peaks. The two black dashed lines in Figure 9 show the heights of the two largest second derivative peaks, and the dashed red line shows the largest third derivative peak located between them.

**Figure 10:**
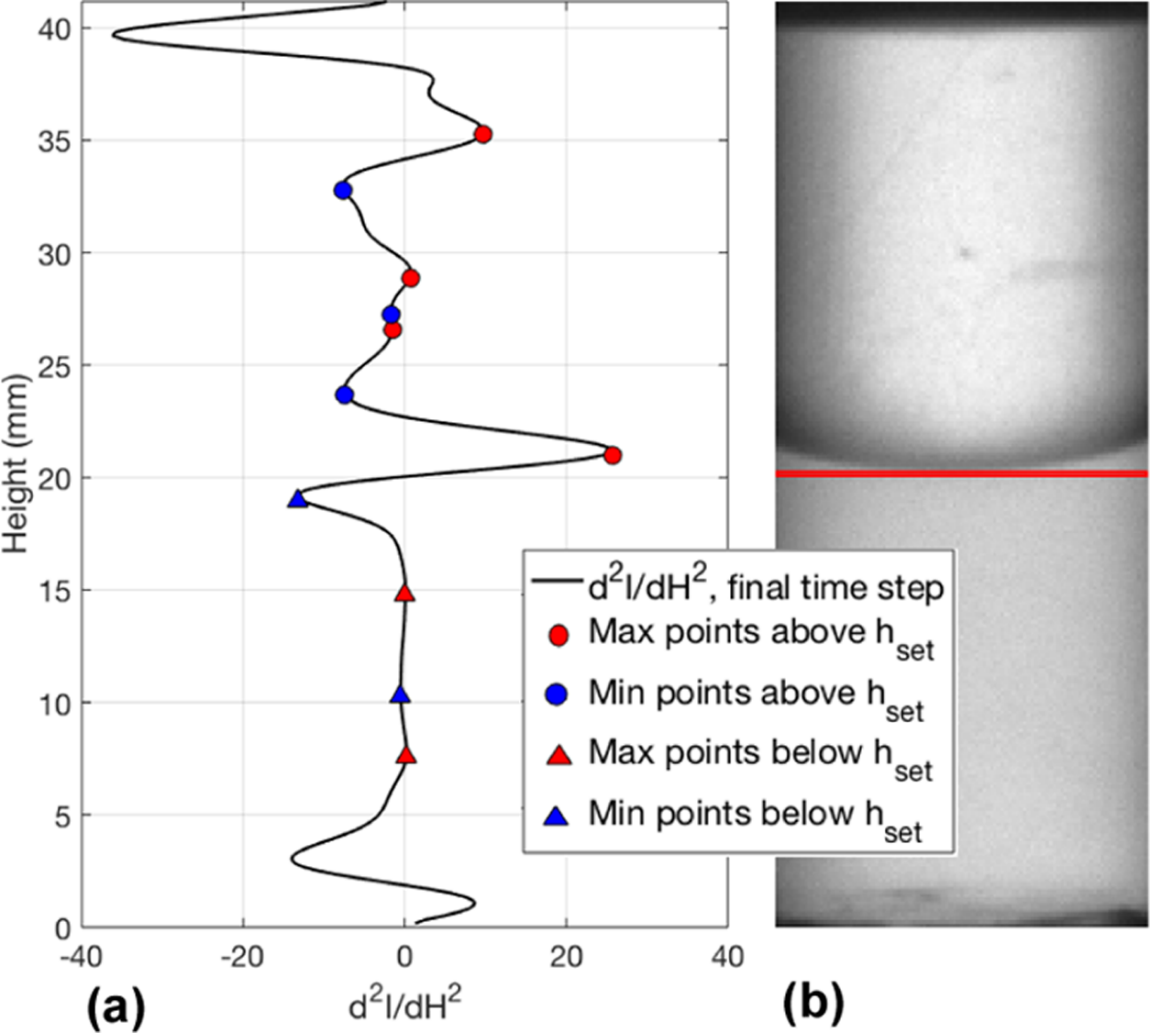
Second derivative of the final timesteps normalized and smoothed grayscale height data and the located maximum and minimum points above and below *h*_set_ (a). The corresponding grayscale image with *h*_set_ location (b).

Once *h*_set_ has been found, the results from steps 5, 6, and 7 in the flowchart can be used to determine the first search direction and search start location via steps 8–10. In step 5, the average of the maximum and minimum second derivative peaks above and below *h*_set_, and across every timestep are calculated (d2_MxA_, d2_MnA_, d2_MxB_, d2_MnB_). The located maximum and minimum peaks above and below *h*_set_ for the final timestep are shown in Figure 10.

During step 6, the average grayscale values 10 pixels above *h*_set_ (*I*_Ave_top__) and 10 pixels below *h*_set_ (*I*_Ave_bottom__) are calculated (shown by the orange and pink rectangles in Figure 9b). If the area just above *h*_set_ is darker than the area just below *h*_set_, then *h*_set_ is at the lower bound of the interface (as seen in Figure 10), otherwise *h*_set_ is at the upper bound. Steps 8–10 in the flowchart are a series of logical operations depending on if *I*_Ave_top__ or *I*_Ave_bottom__ is larger and if d2_MnA_ or d2_MnB_ is smaller. The aim of this step is to ensure that the first search area passes through the interface. To do this, the result from step 6 is analyzed to find out if *h*_set_ is at the upper or lower bound of the interface. If it is as the upper bound, then the first search area is below *h*_set_, if it is at the lower bound, then the first search area is above *h*_set_. Second, the search algorithm works best when the first detected interface is the most “distinct” in the image series. To check whether the top or bottom settling fronts is more distinct, d2_MnA_ and d2_MnB_ are compared. If d2_MnA_ is less than d2_MnB_, then the top settling front is more distinct than the bottom interface. To ensure that both conditions are true, *h*_set_ is shifted to the next third derivative peak below *h*_set_ if *I*_Ave_top__ > *I*_Ave_bottom__ and d2_MnA_ > d2_MnB_ or to the next third derivative peak above *h*_set_ if *I*_Ave_top__ < *I*_Ave_bottom__ and d2_MnA_ < d2_MnB_. Shifting *h*_set_ in this way moves it to the other side of the interface so that the first search area can pass through the interface and detect the most distinct interface first.

**Figure 11:**
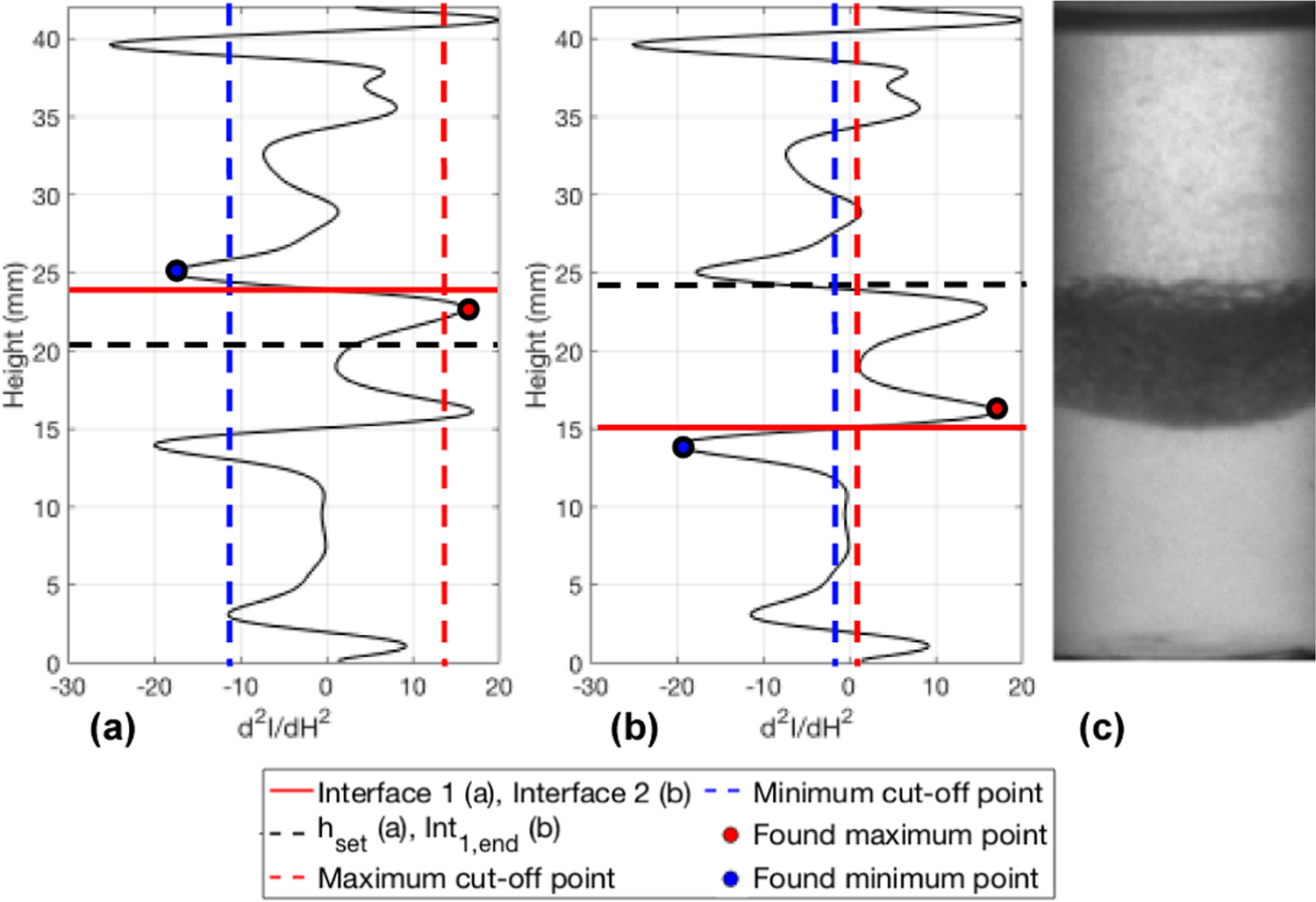
Second derivative data at 10 s with the location of interface 1 determined by the found maximum and minimum points (a). The second derivative data at 10 s with the location of interface 2 determined by the found maximum and minimum points (b). The corresponding image at 10 s (c).

At this point, to keep the algorithm universal to all cases whether the first search area is the top half or the bottom half Δ*I*_h,t_, , , and  are flipped with respect to their height in the vessel if the first search area is the bottom half. The data is flipped back to its original form after the search algorithm is complete. This step ensures that any logic operations within the detection algorithm based on the position of maxima or minima within the vessel are the same. The derivatives d2_MxA_, d2_MnA_, d2_MxB_, and d2_MnB_ are then recalculated with respect to the new *h*_set_ (step 11). These values are used to set a cutoff value at which a given maxima or minima is decided to be significant. For example, if a maximum point above *h*_set_ is larger than d2_MxA_, it is significant. If the cutoff values work well, they will track the interface accurately over each timestep. This is not always the case if d2_MxA_, d2_MnA_, d2_MxB_, and d2_MnB_ are the only cutoff thresholds considered. To select a threshold that tracks the interface accurately, several threshold values should be tried. In step 12, each d2 value is multiplied by 0.1, 0.55, 1, 1.5, and 2 to produce four 5 × 5 vectors. This gives 25 combinations of d2_Mx_ and d2_Mn_ to try for each interface. These multiplication factors were set after a period of trial and error with the algorithm. The range of values produced by these vectors consistently produced at least one case from the 25 pairs that tracked the interface well, for each of the sample cases studied. Table 2 shows the vectors produced for the sample toluene and water case. The red cells correspond to the maxima and minima cutoff thresholds shown in Figure 11.

**Table 2:** Test Maximum and Minimum Cutoff Points for Interfaces 1 and 2 Shown in Bold

Interface 1 Cutoff Vectors					
maximum cutoff threshold	1.01	5.55	10.08	**15.13**	20.17
minimum cutoff threshold	–0.71	–3.89	–7.07	–10.61	**–14.14**

Interface 2 Cutoff Vectors					
maximum cutoff threshold	**0.07**	0.36	0.66	0.99	1.32
minimum cutoff threshold	–0.52	**–2.85**	–5.19	–7.78	–10.37

In step 13, the algorithm searches above *h*_set_ (shown in Figure 11a by the black dashed line) to find interface 1 and selects the first maximum (red dot) larger than the maximum cutoff threshold (red dashed line) and the first minimum above this maximum (blue dot) that is less than the minimum cutoff threshold (blue dashed line). The inflection point between these two is the location of interface 1 (red line). This process is repeated for every timestep and every max and min cutoff threshold combination. The best combination of maximum and minimum cutoff values is decided based on the interface height vs time data and the *R*^2^ fit with the sigmoidal curve given by eq 2, where *c* = Int_1,1_ and *d* = Int_1,end_ (the first and last interface heights given by the algorithm). The constants *a* and *b* in eq 2 are determined by the curve fit algorithm: where a determines the “steepness” of the sigmoidal curve and was limited in the algorithm to ±5, and *b* is the *x* data point at which the curve has reached half of its final height (Int_1,end_/2). There was no limit imposed on what value could be calculated for *b* except for repeats 1–3 of the pH 4, 0.25 phase ratio cases and the 0.46 HLD vial. The reasons for this are discussed in the relevant results sections. Interface_fit_ is the interface height as determined by the sigmoidal curve fit.
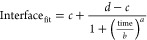
2

**Figure 12:**
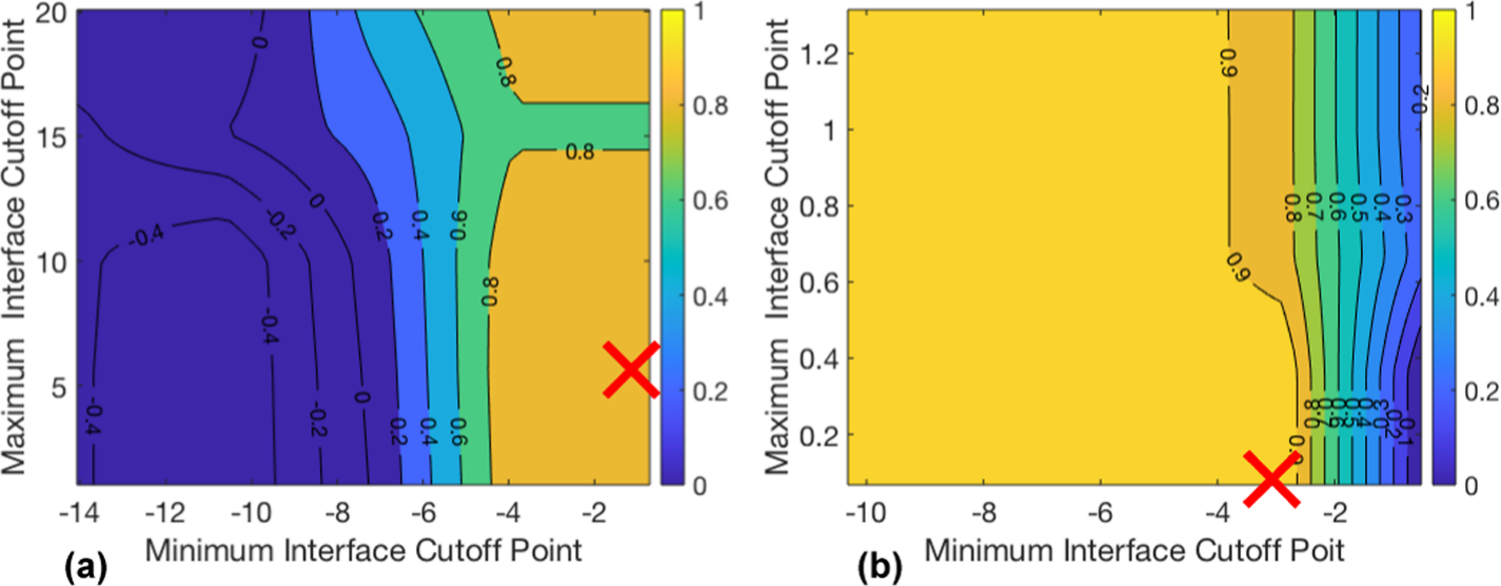
Contour plot of *R*^2^ values for the sigmoidal curve fit for interface 1 data (a) and interface 2 data (b) depending on the maximum and minimum cutoff point combination.

A sigmoidal curve models the settling of an emulsion well, and therefore it was assumed that when the data fit the sigmoidal curve best (highest *R*^2^ value), the algorithm has tracked the interface better than any of the other maximum and minimum cutoff threshold combinations (steps 14–16). A contour plot of the *R*^2^ values obtained from the 25 maximum and minimum cutoff threshold pairs is shown in Figure 12 for interface 1 (a) and interface 2 (b).

There tended to be a large portion within the search area that had the same or very similar best *R*^2^ value and therefore any of the cutoff threshold pairs within that area could be selected. The actual selected max and min cutoff values were the values closest to 0 within the area that had the same *R*^2^ values and are shown by the red X’s in Figure 12. Once the best maximum and minimum threshold values for interface 1 were selected, a similar procedure to determine interface 2 was undertaken (steps 17–20).

The search start position for interface 2 was now Int_1,end_ (black dashed line on Figure 11b) so that any maxima and minima below the final position of interface 1 would be detected. As with interface 1, the 25 maximum and minimum cutoff threshold combinations were tested. Figure 11b shows the cutoff points (red and blue dashed lines) for interface 2. The search for interface 2 is slightly different from the search for interface 1 as the first minimum below Int_1,end_ is found first (blue dot) and then the first maximum above that (red dot). The inflection point between these two points is then selected as interface 2 (red line). This is done so that the same maximum can be selected for both interface 1 and interface 2 but the inflection points chosen are either side of that maximum, this is how the upper and lower bound of the final interface can be found. Figure 11 shows how the algorithm works at a single timestep (10 s), the process depicted here is used for all timesteps, first searching for interface 1 and then interface 2. The same search procedure for the best-fitting curve is applied to interface 2. The *R*^2^ results for interface 2 are shown in Figure 12b.

**Figure 13:**
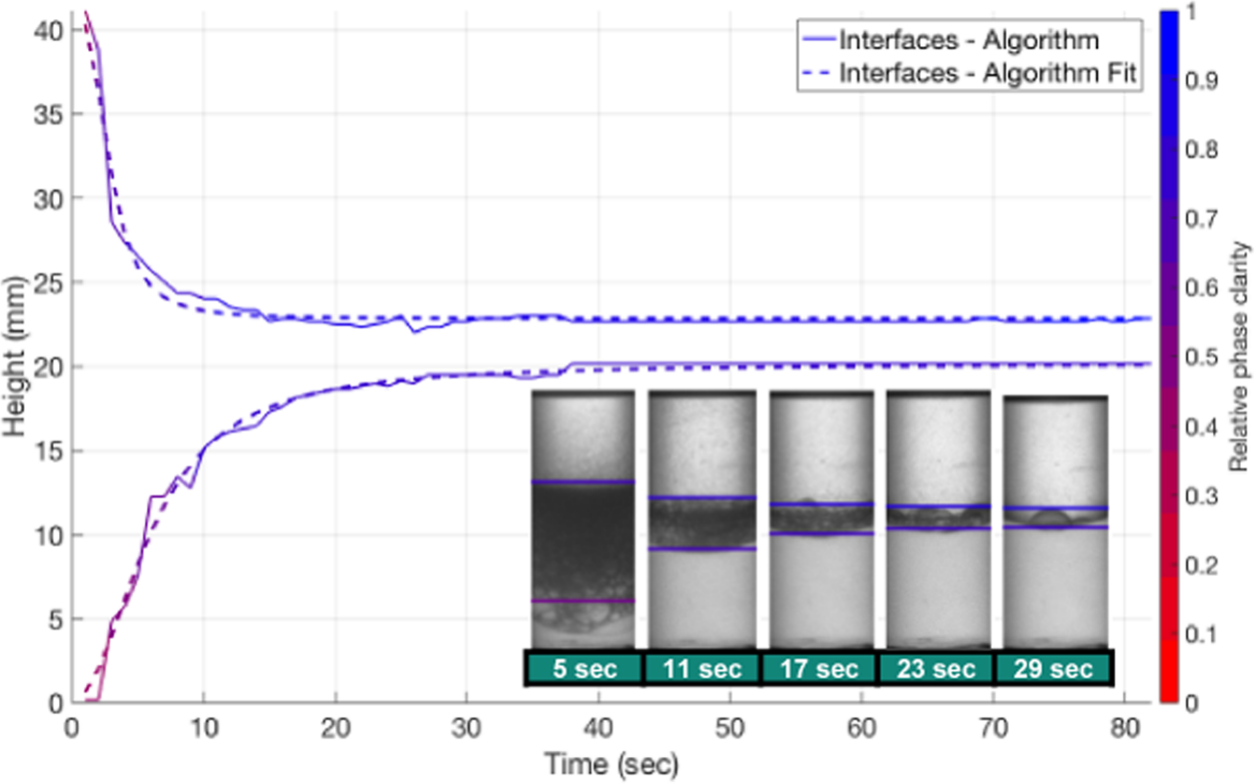
Top and bottom interface heights of example toluene–water case with relative phase clarity over time and sample images annotated with corresponding interface locations and relative phase clarity at selected timesteps.

Once interfaces 1 and 2 have been found, the grayscale values in the area above interface 1 and below interface 2 are averaged for each timestep and normalized with respect to the average grayscale value for pure toluene in the case of the top interface and pure water in the case of the bottom interface (steps 21 and 22). This is done to give an indication of the clarity of the “settled” region at each timestep relative to a pure liquid phase (shown by the color bar in Figure 13 as the “relative phase clarity”). A relative phase clarity of 0 indicated a very dark region that has not settled at all. A relative phase clarity of 1 indicates the settled area is very clear and has no residual droplets or fine dispersions. For example, in Figure 13, there are some large bubbles still below the bottom interface at 5 s. These bubbles darken the area below the interface (reduced average grayscale value) and therefore result in a lower relative phase clarity. In the case of stable emulsions, a settling front may be detected, but fine droplets which have not settled via sedimentation or creaming may exist that cloud the area above or below the interface and reduce the relative phase clarity. This can give an indication of the emulsion type, if the bottom phase is darkest and fine droplets can be seen then that suggests the emulsion was O/W and vice versa for the top phase.

The procedure described above has been applied in the same way to each case in the buffer solution experiments and the surfactant solution experiments. The overall search area changed depending on the size of the liquid vessel but was otherwise consistent. The length of a single pixel was required to convert number of pixels to a physical distance. In the buffer solution experiments, this was 0.1 μm, and in the surfactant solution experiments, this was 0.168 μm. The difference in pixel length depended upon the camera’s zoom settings. The pure toluene and water settings were changed depending on the light level settings used for each experiment. An overview of the constants used for each image set and the algorithmically determined key values such as the applied threshold values are given in Sections 2.0 and 3.0.

### Scale-Up Experiments

Five experimental runs were taken from small scale and reproduced at 20 L: two cases from the toluene and aqueous solution experiments and three cases from the surfactant solution experiments. The toluene and deionized water case and the glycine buffer solution case were tested at a phase ratio of 4. The aqueous solution (16 L) was prepared in both cases and added to the reaction vessel. 4 L of toluene was added and stirred at 242 rpm for 10 min before agitation was stopped. A series of images were taken while the liquid–liquid mixtures settled in the reaction vessel. The location of the liquid interfaces at each timestep was determined via manual image analysis.

The three surfactant experiments selected for scale-up were at HLD values of −0.49, 0, and 0.45. SDBS (0.01 M) was added to 10 L of deionized water and 166.5, 276.7, or 433.3 g of salt was added to each solution to reach the desired HLD value. 10 L of toluene was then added to the vessel and mixed at 242 rpm for 10 min. Once agitation was stopped, a series of images were taken while the liquid–liquid mixtures settled. The location of the liquid interfaces at each timestep was determined via manual image analysis.

## Data Availability

Research data is available within the Supporting Information.

## Supporting Information Available

Description of the algorithm in a logic diagram, inputs and constants used within the algorithm, output of the algorithm in terms of the parameters used within the interface position determination, images of each experiment in triplicate, final separation curves together with statistical data, design of an automated shaking rig, and brief description of the HLD method (PDF)
