# Supplementary material for: Determining Phase Separation Dynamics with an Automated Image Processing Algorithm
Source: Org Process Res Dev. 2023 Mar 14;27(4):627–39. doi: 10.1021/acs.oprd.2c00357 (PMC10127267; doi:10.1021/acs.oprd.2c00357)
Supplement: Supplementary file 1 — op2c00357_si_001.pdf [file op2c00357_si_001.pdf]

Determining phase separation dynamics with an automated image processing algorithm

James Daglish[1], A. John. Blacker[2], Gregory de Boer[1], Alex Crampton[3], David R. J. Hose[3], Anna R. Parsons[3], Nikil Kapur[1*].
[1]School of Mechanical Engineering, University of Leeds, LS2 9JT,
[2]School of Chemistry, University of Leeds, LS2 9JT.
[3]Chemical Development, Pharmaceutical Technology and Development, Operations, AstraZeneca, Macclesfield, SK10 2NA, UK.

*n.kapur@leeds.ac.uk

Experimental Supplementary Information

Contents

1.0 Algorithm logic diagram

2.0 Algorithm inputs and constants

2.1 Low-pass filter parameters

2.2 Experiment specific constants

2.3 Sigmoidal curve fitting inputs

3.0 Algorithm outputs

3.1 Sigmoidal curve fitting outputs

3.2 Maximum and minimum thresholds

4.0 Case figures and images

4.1 Experiment 1

4.2 Experiment 2

4.3 Scale up experiments

5.0 Experimental rig development

6.0 A summary of the HLD method

1.0 Algorithm logic diagram

The logic diagram shown in figure S1 depicts each operation performed by the algorithm to find the interface between settling phases over a period of time.

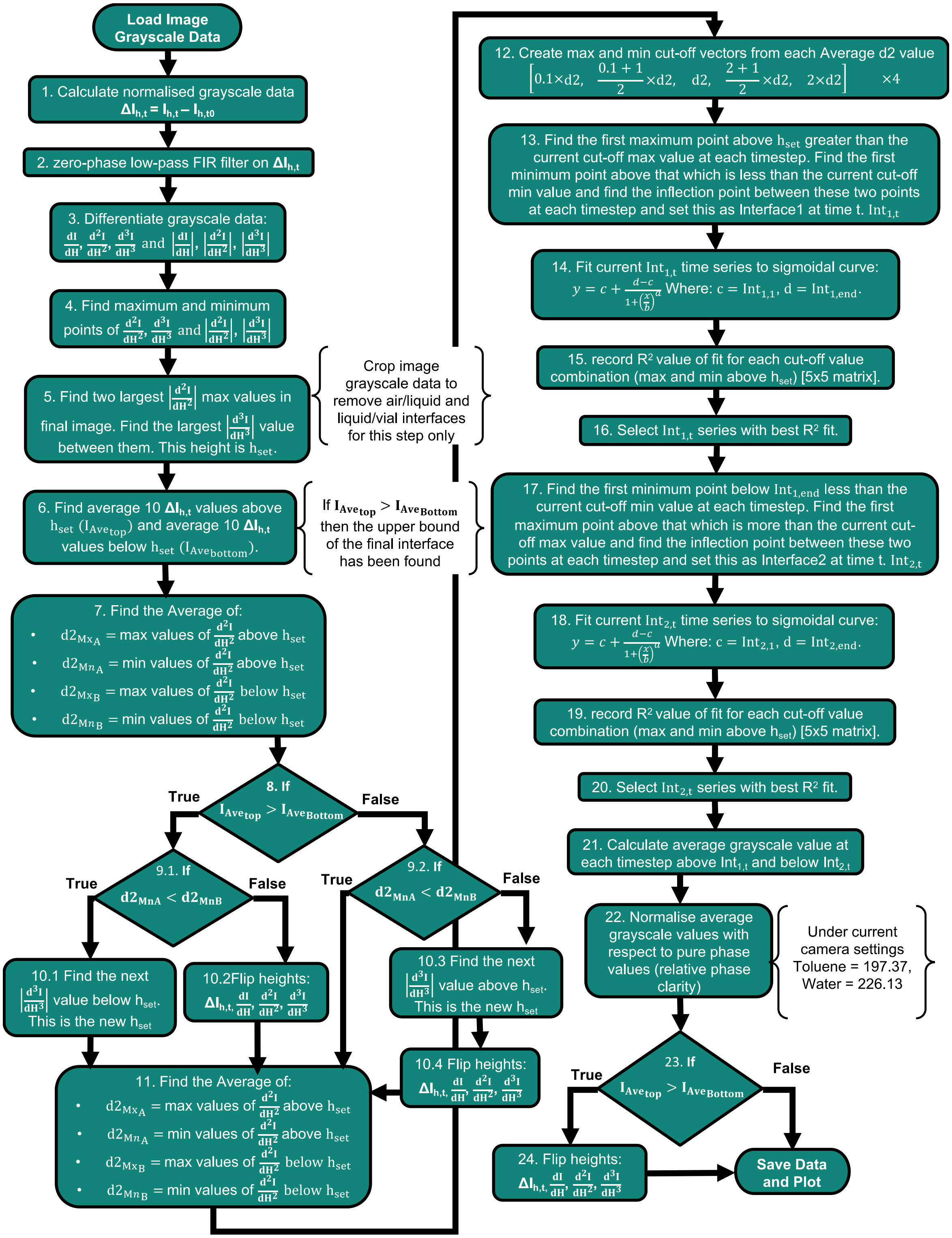

Figure S1: Flowchart detailing the interface detection image processing algorithm

2.0 Algorithm Inputs and Constants

In this section the algorithm inputs and constant values are detailed. This includes the parameters of the low-pass FIR filter, the sigmoidal curve fitting limits, the pure liquid average grayscale values used to normalised the grayscale data, search areas, pixel lengths and sample rates. Phase ratio was defined as V_{Aq}/V_{Org} and experiment 1 corresponds to the liquid biphasic mixtures while experiment 2 corresponds to the surfactant system experiments.

2.1 Low-pass FIR filter

The filter used to smooth the collected grayscale height data was a zero-phase finite impulse response filter (FIR). The Matlab function 'filtfilt' was used to construct the filter and was applied to each case in step 2 of the flow chart S1. Four parameters are required for this function; passband frequency, stopband frequency, passband ripple, and stopband attenuation. The values selected for each of these parameters is shown in table S1.

Table S1: Low-pass FIR filter parameters used during experiments 1 and 2

Filter Parameters	Experiment 1 – Phase ratio = 0.25, 1	Experiment 1 – Phase ratio = 4	Experiment 2
Passband Frequency	0.025	0.025	0.025
Stopband Frequency	0.1	0.6	0.3
Passband Ripple	1	1	1
Stopband Attenuation	100	100	100

2.2 Experiment specific constants

The constants detailed in table S2 are set before running the algorithm for each experiment. The sample rate is the time between each image captured. The average grayscale intensity of toluene and water varied between experiments as the contrast of the camera was varied between experiments. The pixel length was changed as the camera zoom settings were changed between experiments. The number of pixels to crop from the top and bottom of the image for steps 5,7 and 11 of the algorithm procedure remained the same except for vial 7 in experiment 2. The number of overall pixels affects what value this should be set at. The search distance to calculate I_{Avetop} and $I_{Avebottom}$ remained the same for both experiments.

Table S2: Constants used in the algorithm that are specific to each experimental setup

	Sample Rate (sec)	Average grayscale intensity of pure toluene	Average grayscale intensity of pure water	Pixel length (µm)	Number of pixels to crop (step 5,7, and 11)	Search distance for I_{Avetop} and $I_{Avebottom}$ (pxls)
Experiment 1	1	172.71	182.65	100	30	10
Experiment 2	10	197.38	226.13	168	30	10

Table S3 catalogues the horizontal and vertical starting pixel and the horizontal and vertical number of pixels (width and height) that the algorithm used for each experiment run in experiment 1, table S4 catalogues the same values for experiment 2. Ideally these values would not change within a single experiment as the liquid volume would be closely controlled along with the vessel

location within the cameras field of view. However, as the focus of this work was on the algorithm development, creating an experimental apparatus that kept these parameters consistent was not a priority. The development of such an apparatus would remove the variance in start position of the vessel within the cameras field of view and would eliminate variance in the values stated in Table S3.

Table S3: Horizontal and vertical starting pixel and the horizontal and vertical number of pixels (width and height) for each case in experiment 1.

Aqueous solution	Phase ratio	Repeat no	x start	y start	x length	y length
toluene:acetate	0.25	1	700	230	420	660
toluene:acetate	0.25	2	700	230	420	660
toluene:acetate	0.25	3	700	230	420	660
toluene:deionised water	0.25	1	690	240	420	660
toluene:deionised water	0.25	2	690	225	420	660
toluene:deionised water	0.25	3	690	220	420	660
toluene:glycine	0.25	1	690	235	420	670
toluene:glycine	0.25	2	690	220	420	670
toluene:glycine	0.25	3	690	220	420	670
toluene:acetate	1	1	675	235	420	660
toluene:acetate	1	2	680	230	420	660
toluene:acetate	1	3	680	225	420	660
toluene:deionised water	1	1	690	230	420	670
toluene:deionised water	1	2	680	225	420	670
toluene:deionised water	1	3	675	225	420	670
toluene:glycine	1	1	690	235	420	670
toluene:glycine	1	2	680	225	420	670
toluene:glycine	1	3	680	220	420	670
toluene:acetate	4	1	680	235	420	670
toluene:acetate	4	2	680	220	420	670
toluene:acetate	4	3	680	220	420	670
toluene:deionised water	4	1	700	230	420	670
toluene:deionised water	4	2	670	225	420	670
toluene:deionised water	4	3	665	220	420	670
toluene:glycine	4	1	695	235	420	670
toluene:glycine	4	2	685	225	420	670
toluene:glycine	4	3	685	225	420	670

Table S4: Horizontal and vertical starting pixel and the horizontal and vertical number of pixels (width and height) for each case in experiment 2.

Vial Number	SDBS Concentration (M)	Salinity (g/100ml)	HLD value	x start	y start	x length	y length
1	0.01	0.0453	-3.38	365	275	100	255

2	0.01	1.076	-0.91	505	275	100	255
3	0.01	1.6627	-0.49	645	275	100	255
4	0.01	2.172	-0.23	785	275	100	255
5	0.01	2.768	0.006	925	275	100	255
6	0.01	3.372	0.2	335	280	100	255
7	0.01	4.3387	0.45	475	280	100	255
8	0.01	5.7133	0.72	615	280	100	255
9	0.01	6.6480	0.87	755	275	100	260
10	0.01	7.86	1.04	895	275	100	260
11	0.1	0.0413	-2.99	315	275	100	255
12	0.1	1.076	-0.87	455	275	100	255
13	0.1	1.5907	-0.5	595	275	100	255
14	0.1	2.2213	-0.19	740	275	100	255
15	0.1	2.68	0.007	880	275	100	255
16	0.1	3.2867	0.19	390	275	100	255
17	0.1	4.348	0.46	530	275	100	255
18	0.1	5.6587	0.72	670	275	100	255
19	0.1	6.6507	0.88	810	275	100	255
20	0.1	7.7937	1.04	950	275	100	255

2.3 Sigmoidal curve fitting inputs

Table S5 shows the imposed limits on the sigmoidal curve fitting equation for experiment 1. All of the cases in experiment 1 had the same limits imposed on constant a (±5), only the toluene:acetate cases at a phase ratio of 0.25 had a different limit imposed on constant b (±20). Table S6 shows the imposed limits on the sigmoidal curve fitting equation for experiment 2. All of the cases in experiment 2 had the same limits imposed on constant a (±5), only vial 7 had a different limit imposed on constant b (±100). Constants c and d were set by the algorithm depending on the 1st and last interface height detected by the algorithm in both experiments.

Table S5: Imposed limits on the sigmoidal curve fitting equation constants a, b, c, and d for interface 1 and 2 in experiment 1.

Aqueous solution	Phase ratio	Repeat no	a (limits)	b (limits)	Interface 1 c (fixed)	Interface 1 d (fixed)	Interface 2 c (fixed)	Interface 2 d (fixed)
toluene:acetate	0.25	1	±5	±20	0.05	5.58	64.51	9.20
toluene:acetate	0.25	2	±5	±20	0.15	5.70	65.26	9.20
toluene:acetate	0.25	3	±5	±20	0.90	6.64	64.42	10.20
toluene:deionised water	0.25	1	±5	±600	0.00	6.20	65.87	9.70
toluene:deionised water	0.25	2	±5	±600	0.00	7.10	64.74	10.30
toluene:deionised water	0.25	3	±5	±600	0.24	7.40	58.32	10.90
toluene:glycine	0.25	1	±5	±600	0.00	7.50	59.05	11.40
toluene:glycine	0.25	2	±5	±600	0.00	7.20	66.85	10.40

toluene:glycine	0.25	3	±5	±600	0.00	7.50	66.22	10.90
toluene:acetate	1	1	±5	±600	0.00	29.80	65.90	32.70
toluene:acetate	1	2	±5	±600	66.00	32.30	0.10	29.40
toluene:acetate	1	3	±5	±600	66.00	32.60	0.10	29.80
toluene:deionised water	1	1	±5	±600	0.59	29.50	66.82	32.80
toluene:deionised water	1	2	±5	±600	1.98	30.40	66.75	33.70
toluene:deionised water	1	3	±5	±600	5.12	30.90	66.89	34.10
toluene:glycine	1	1	±5	±600	0.00	30.20	66.90	33.20
toluene:glycine	1	2	±5	±600	0.03	29.80	66.54	33.00
toluene:glycine	1	3	±5	±600	0.09	30.10	66.89	33.30
toluene:acetate	4	1	±5	±600	66.98	53.30	0.14	52.16
toluene:acetate	4	2	±5	±600	66.99	52.80	0.11	51.65
toluene:acetate	4	3	±5	±600	66.98	52.50	0.10	50.28
toluene:deionised water	4	1	±5	±600	65.06	51.37	6.52	50.70
toluene:deionised water	4	2	±5	±600	65.86	50.91	4.11	50.40
toluene:deionised water	4	3	±5	±600	65.08	52.91	0.32	51.50
toluene:glycine	4	1	±5	±600	66.78	52.57	0.10	50.72
toluene:glycine	4	2	±5	±600	66.96	53.22	0.10	52.23
toluene:glycine	4	3	±5	±600	66.93	53.61	0.10	52.67

Table S6: Imposed limits on the sigmoidal curve fitting equation constants a, b, c, and d for interface 1 and 2 in experiment 2.

					**Interface 1**		**Interface 2**	
**Vial Number**	**SDBS Concentration (M)**	**HLD value**	**a (limits)**	**b (limits)**	**c (fixed)**	**d (fixed)**	**c (fixed)**	**d (fixed)**
1	0.01	-3.38	±5	±7200	0.00	15.57	42.67	42.67
2	0.01	-0.91	±5	±7200	0.00	10.98	42.58	26.05
3	0.01	-0.49	±5	±7200	42.13	23.41	3.61	20.33
4	0.01	-0.23	±5	±7200	0.00	13.10	38.29	21.67
5	0.01	0.006	±5	±7200	42.33	22.18	0.17	19.31
6	0.01	0.2	±5	±7200	42.62	22.85	0.18	20.16
7	0.01	0.45	±5	±100	42.56	23.02	13.75	21.28
8	0.01	0.72	±5	±7200	41.91	23.35	0.17	20.33
9	0.01	0.87	±5	±7200	42.35	23.35	0.17	20.50
10	0.01	1.04	±5	±7200	0.00	19.15	42.96	22.69
11	0.1	-2.99	±5	±7200	0.00	7.49	37.87	25.03
12	0.1	-0.87	±5	±7200	41.76	40.37	6.21	39.65
13	0.1	-0.5	±5	±7200	0.00	12.73	36.61	23.02
14	0.1	-0.19	±5	±7200	42.84	29.47	4.19	15.12
15	0.1	0.007	±5	±7200	42.84	31.08	5.91	16.46
16	0.1	0.19	±5	±7200	42.84	31.93	0.24	21.67

17	0.1	0.46	±5	±7200	42.84	32.20	6.81	20.33
18	0.1	0.72	±5	±7200	42.84	31.92	6.35	17.81
19	0.1	0.88	±5	±7200	42.84	31.63	7.21	7.22
20	0.1	1.04	±5	±7200	42.84	30.35	4.53	19.32

3.0 Algorithm outputs

In this section the outputs of the algorithm are detailed. This includes the r^2 value of each sigmoidal fit and the constants determined by the curve fit a and b, the selected maximum and minimum cut-off values for interface 1 and 2 and the d2 multiplication factor used to set those maximum and minimum cut-off values.

3.1 Sigmoidal curve fitting outputs

Table S7 shows the constants a and b and the r^2 value determined by the curve fitting process for each interface in each case for experiment 1. The same values are shown in table S8 for experiment 2.

Table S7: Outputs of the sigmoidal curve fit for experiment 1.

Aqueous solution	Phase ratio	Repeat no	Interface 2			Interface 2		
			a (fit)	b (fit)	r2	a (fit)	b (fit)	r2
toluene:acetate	0.25	1	1.64	18.41	0.49	3.33	3.02	0.91
toluene:acetate	0.25	2	1.73	7.97	0.90	5.00	2.44	0.94
toluene:acetate	0.25	3	1.02	6.22	0.84	5.00	2.05	0.97
toluene:deionised water	0.25	1	5.00	14.12	0.94	5.00	4.46	0.94
toluene:deionised water	0.25	2	4.15	10.64	0.99	5.00	2.16	0.98
toluene:deionised water	0.25	3	1.74	7.12	0.93	5.00	1.44	0.96
toluene:glycine	0.25	1	4.10	12.00	0.96	5.00	1.43	0.94
toluene:glycine	0.25	2	5.00	16.75	0.99	5.00	4.03	0.96
toluene:glycine	0.25	3	5.00	13.03	0.98	5.00	2.41	0.94
toluene:acetate	1	1	3.33	15.25	0.99	3.27	25.14	0.95
toluene:acetate	1	2	3.33	15.43	0.98	4.63	13.36	0.99
toluene:acetate	1	3	3.92	17.89	0.97	5.00	13.91	0.98
toluene:deionised water	1	1	2.25	5.62	0.96	2.44	12.03	0.97
toluene:deionised water	1	2	2.27	3.23	0.98	3.13	5.57	0.96
toluene:deionised water	1	3	1.74	2.53	0.96	4.18	8.08	0.98
toluene:glycine	1	1	3.87	16.07	0.96	3.32	17.15	0.97
toluene:glycine	1	2	3.49	6.94	1.00	2.36	6.86	0.94
toluene:glycine	1	3	2.88	7.50	1.00	3.14	12.11	0.96
toluene:acetate	4	1	2.47	14.02	0.97	2.25	23.93	0.88
toluene:acetate	4	2	2.70	15.26	0.97	2.38	31.91	0.95
toluene:acetate	4	3	2.97	9.76	0.98	2.68	34.92	0.94

toluene:deionised water	4	1	1.16	5.43	0.87	5.00	1.47	0.96
toluene:deionised water	4	2	1.65	4.76	0.95	2.48	2.68	0.95
toluene:deionised water	4	3	1.46	3.55	0.89	3.11	5.79	0.98
toluene:glycine	4	1	1.48	16.57	0.93	3.48	94.79	0.97
toluene:glycine	4	2	1.95	20.40	0.97	3.21	77.22	0.96
toluene:glycine	4	3	1.93	15.54	0.95	3.21	58.49	0.97

Table S8: Outputs of the sigmoidal curve fit for experiment 2.

			**Interface 1**			**Interface 2**		
**Vial Number**	**SDBS Concentration (M)**	**HLD value**	**a (fit)**	**b (fit)**	**r^2**	**a (fit)**	**b (fit)**	**r^2**
1	0.01	-3.38	1.89	855.86	0.99	0.00	0.00	-1.21
2	0.01	-0.91	3.14	2576.42	0.62	1.16	983.11	0.21
3	0.01	-0.49	1.42	100.80	0.34	5.00	13.71	0.16
4	0.01	-0.23	3.60	2796.22	0.98	5.00	13.05	0.11
5	0.01	0.006	2.24	51.84	1.00	5.00	1353.27	1.00
6	0.01	0.2	3.24	39.90	0.97	2.85	141.92	0.94
7	0.01	0.45	3.36	35.53	0.95	0.62	4.07	0.33
8	0.01	0.72	3.08	26.44	0.98	3.70	178.59	0.97
9	0.01	0.87	2.87	25.29	0.99	3.23	237.43	0.97
10	0.01	1.04	5.00	653.09	0.99	1.75	78.94	1.00
11	0.1	-2.99	2.69	2743.66	0.99	5.00	12.17	0.01
12	0.1	-0.87	0.67	15.27	0.00	5.00	14.08	0.78
13	0.1	-0.5	2.16	1736.40	0.99	5.00	11.75	0.00
14	0.1	-0.19	2.10	1811.27	0.99	5.00	12.22	0.00
15	0.1	0.007	1.95	1843.20	0.98	5.00	11.29	-0.48
16	0.1	0.19	2.23	1908.48	0.99	5.00	30.96	0.46
17	0.1	0.46	2.13	2085.45	0.99	5.00	11.53	-0.52
18	0.1	0.72	2.00	2104.10	0.98	5.00	11.31	-0.46
19	0.1	0.88	2.17	2281.81	0.99	4.81	2.66	-3.77
20	0.1	1.04	1.82	1584.09	0.99	5.00	12.77	-0.40

3.2 Maximum and minimum thresholds

Table S9 and S10 list the maximum and minimum cut-off values selected by the algorithm during steps 13 – 20 in the interface detection process for experiment 1 and experiment 2.

Table S9: Maximum and minimum cut-off thresholds for interface 1 and 2 in experiment 1.

			**Interface 1**		**Interface 2**	
**Aqueous solution**	**Phase ratio**	**Repeat no**	**Max Cut-off**	**Min Cut-off**	**Max Cut-off**	**Min Cut-off**

toluene:acetate	0.25	1	7.58	-7.03	0.63	-0.52
toluene:acetate	0.25	2	6.93	-5.50	0.77	-0.72
toluene:acetate	0.25	3	8.99	-4.32	0.87	-1.03
toluene:deionised water	0.25	1	2.01	-5.77	0.90	-0.82
toluene:deionised water	0.25	2	9.82	-13.10	0.83	-1.34
toluene:deionised water	0.25	3	11.09	-14.78	1.04	-1.50
toluene:glycine	0.25	1	7.69	-11.68	0.82	-1.21
toluene:glycine	0.25	2	8.56	-13.06	0.43	-0.89
toluene:glycine	0.25	3	9.26	-14.04	0.64	-1.13
toluene:acetate	1	1	4.25	-2.69	1.08	-2.26
toluene:acetate	1	2	4.43	-3.46	1.65	-2.87
toluene:acetate	1	3	3.12	-2.43	0.96	-1.41
toluene:deionised water	1	1	4.67	-2.72	1.04	-2.27
toluene:deionised water	1	2	4.80	-3.91	0.83	-1.59
toluene:deionised water	1	3	5.29	-4.25	1.46	-2.73
toluene:glycine	1	1	5.24	-3.55	0.71	-1.86
toluene:glycine	1	2	5.85	-4.54	0.63	-1.65
toluene:glycine	1	3	5.71	-4.31	0.91	-2.12
toluene:acetate	4	1	17.83	-14.02	2.29	-2.40
toluene:acetate	4	2	11.07	-10.73	2.16	-2.39
toluene:acetate	4	3	11.05	-7.28	2.84	-2.82
toluene:deionised water	4	1	19.08	-9.81	3.68	-6.34
toluene:deionised water	4	2	13.31	-10.58	2.25	-4.83
toluene:deionised water	4	3	16.28	-11.81	2.25	-4.12
toluene:glycine	4	1	17.36	-17.32	2.26	-2.18
toluene:glycine	4	2	17.25	-11.82	2.26	-4.10
toluene:glycine	4	3	23.80	-11.95	2.54	-4.89

Table S10: Maximum and minimum cut-off thresholds for interface 1 and 2 in experiment 2.

			**Interface 1**		**Interface 2**	
**Vial Number**	**SDBS Concentration (M)**	**HLD value**	**Max Cut-off**	**Min Cut-off**	**Max Cut-off**	**Min Cut-off**
1	0.01	-3.38	0.60	-0.45	0.03	-0.03
2	0.01	-0.91	0.06	-0.04	0.03	-0.04
3	0.01	-0.49	0.04	-0.04	0.06	-0.03
4	0.01	-0.23	0.11	-0.07	0.05	-0.03
5	0.01	0.006	10.49	-10.10	1.26	-6.14
6	0.01	0.2	8.85	-9.28	0.49	-1.67
7	0.01	0.45	5.80	-5.05	0.22	-0.99
8	0.01	0.72	7.00	-5.25	0.25	-1.70

9	0.01	0.87	7.41	-4.59	1.60	-2.97
10	0.01	1.04	10.91	-11.84	1.69	-10.64
11	0.1	-2.99	3.46	-2.86	0.04	-0.03
12	0.1	-0.87	0.12	-0.17	0.14	-0.13
13	0.1	-0.5	2.06	-2.29	0.05	-0.02
14	0.1	-0.19	18.11	-24.67	0.05	-0.02
15	0.1	0.007	20.02	-25.09	0.07	-0.01
16	0.1	0.19	19.66	-28.99	0.11	-0.04
17	0.1	0.46	22.07	-29.39	0.10	-0.02
18	0.1	0.72	25.69	-35.73	0.07	-0.01
19	0.1	0.88	26.57	-22.56	0.07	0.00
20	0.1	1.04	21.54	-35.80	0.07	-0.02

4.0 Case figures and images

4.1 Experiment 1

Toluene-acetate – Phase ratio = 0.25, Repeat 1

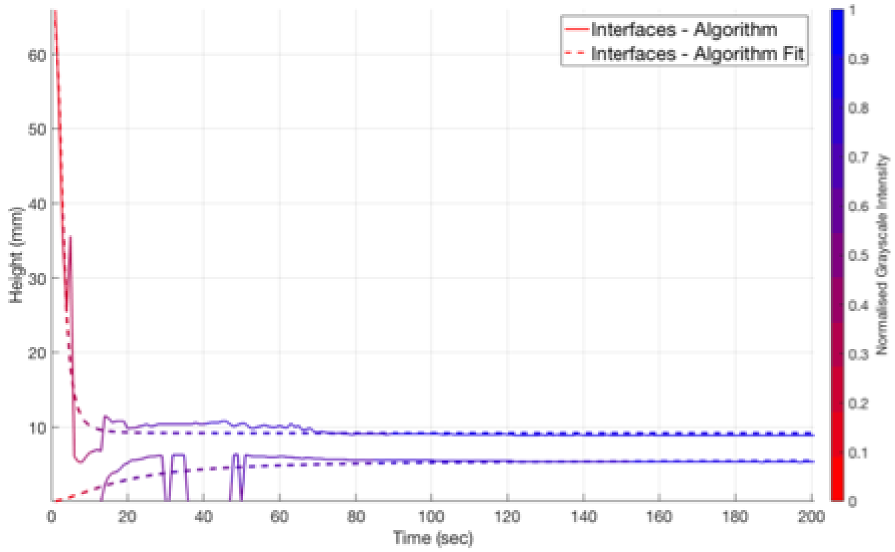

Figure S2: Detected interfaces and normalised grayscale intensity over time for repeat 1 of the toluene-acetate time series at a phase ratio of 0.25.

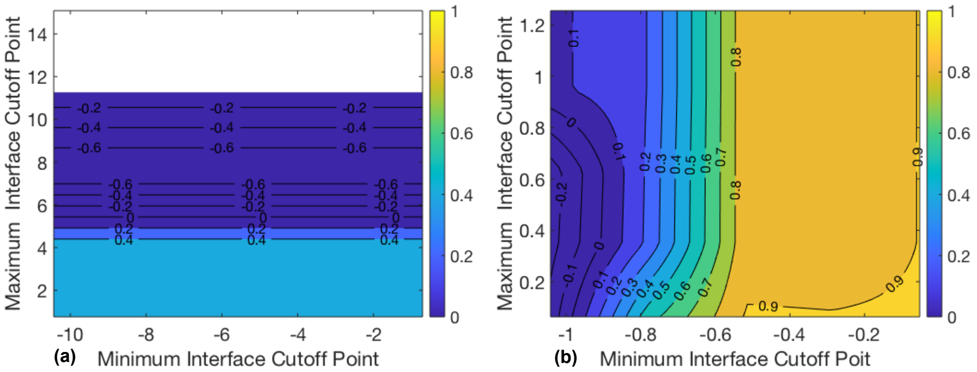

Figure S3: Contour plot of r^2 values for the sigmoidal curve fit for interface 1 data (a) and interface 2 data (b) depending on the maximum and minimum cut-off point combination – Toluene-acetate, phase ratio = 0.25, repeat 1.

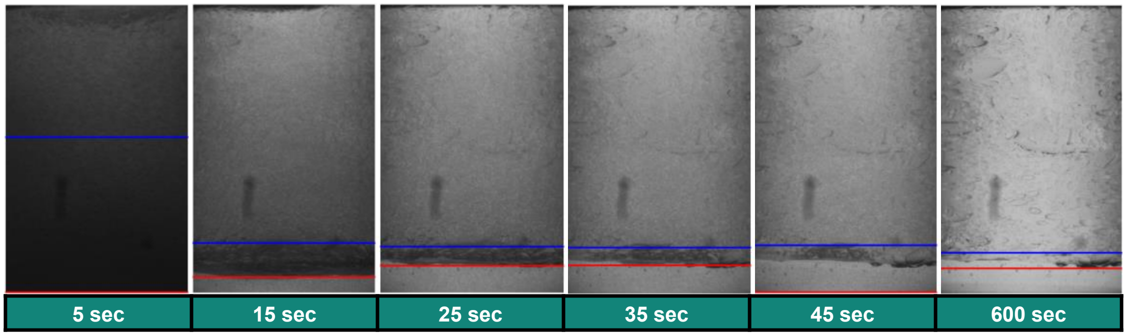

Figure S4: Sample images from toluene-acetate, phase ratio = 0.25, repeat 1 case with location of detected interfaces.

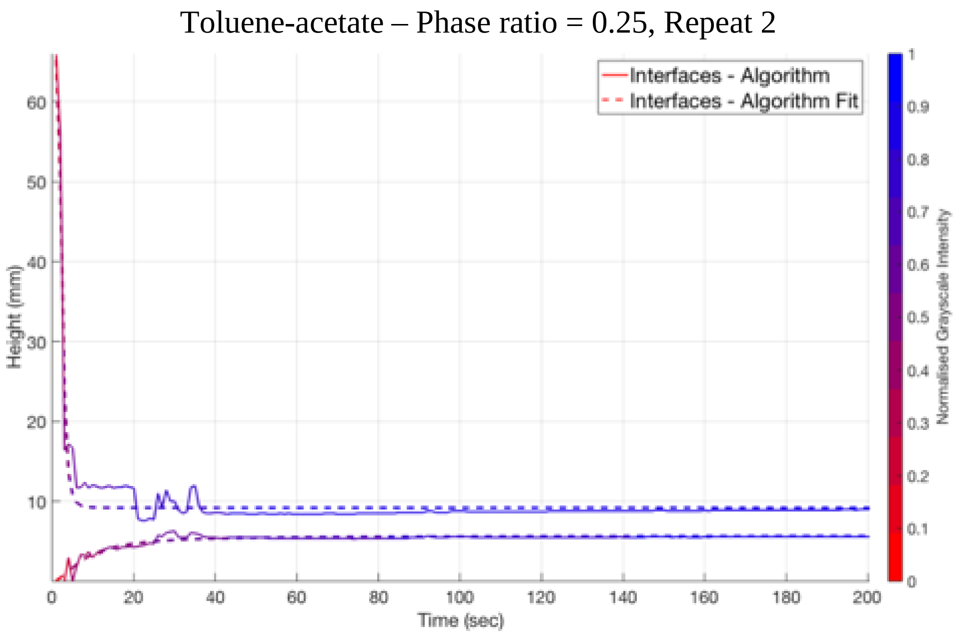

Figure S5: Detected interfaces and normalised grayscale intensity over time for repeat 2 of the toluene-acetate time series at a phase ratio of 0.25.

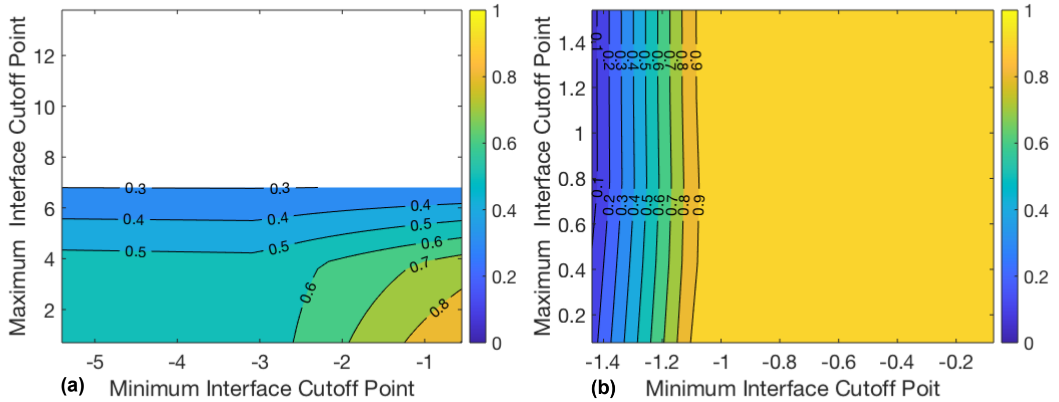

Figure S6: Contour plot of r^2 values for the sigmoidal curve fit for interface 1 data (a) and interface 2 data (b) depending on the maximum and minimum cut-off point combination – Toluene-acetate, phase ratio = 0.25, repeat 2.

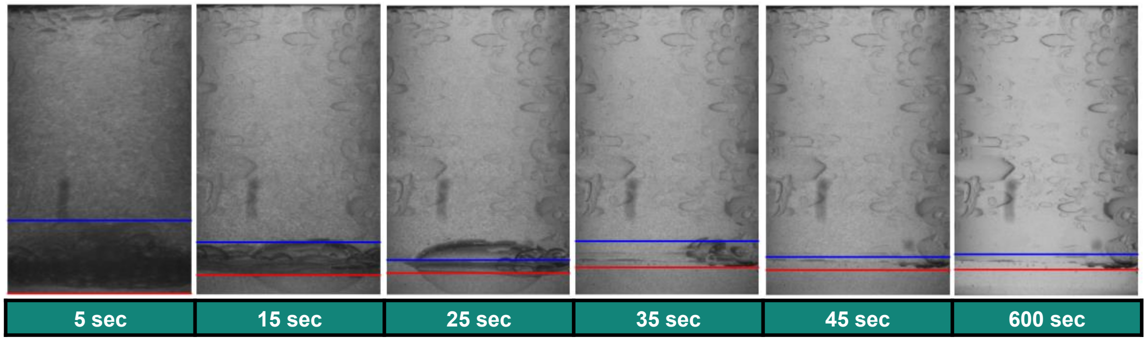

Figure S7: Sample images from toluene-acetate, phase ratio = 0.25, repeat 2 case with location of detected interfaces.

Toluene-acetate – Phase ratio = 0.25, Repeat 3

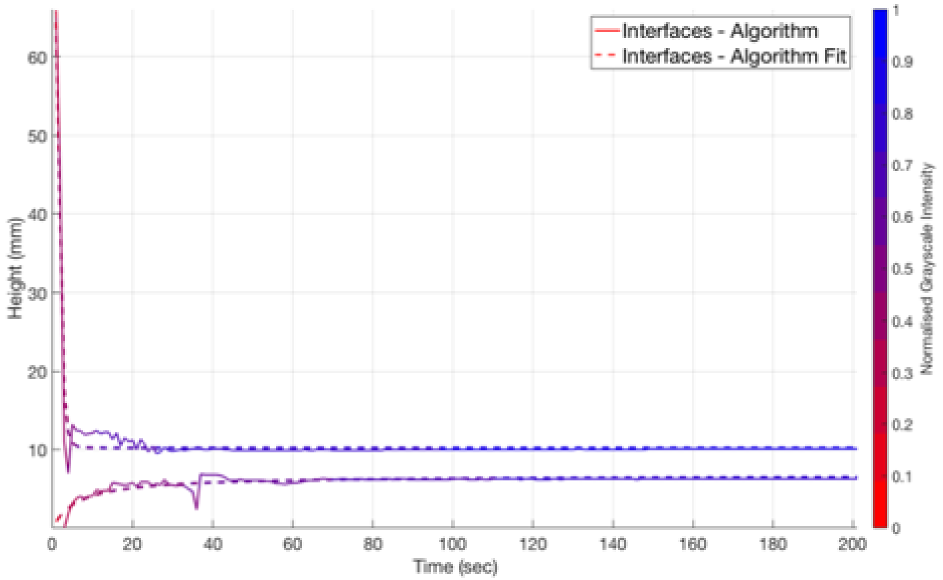

Figure S8: Detected interfaces and normalised grayscale intensity over time for repeat 3 of the toluene-acetate time series at a phase ratio of 0.25.

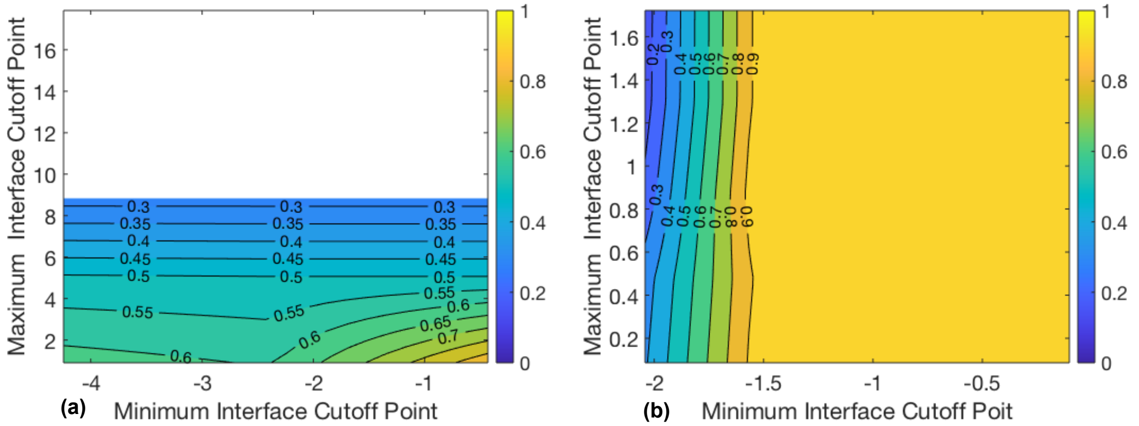

Figure S9: Contour plot of r^2 values for the sigmoidal curve fit for interface 1 data (a) and interface 2 data (b) depending on the maximum and minimum cut-off point combination – Toluene-acetate, phase ratio = 0.25, repeat 3.

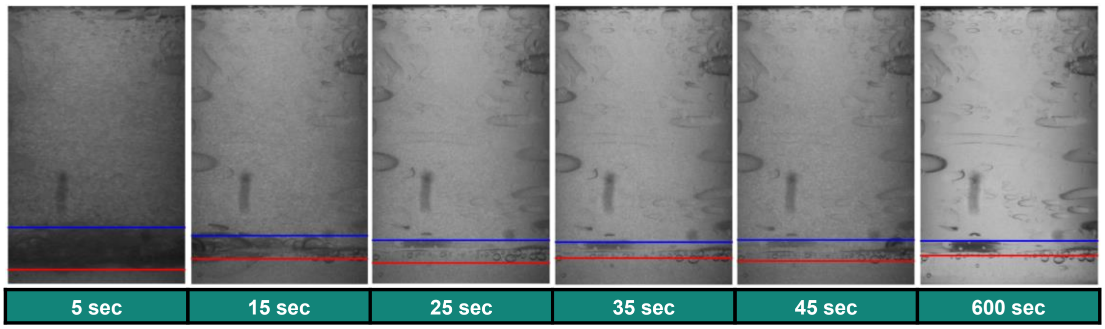

Figure S10: Sample images from toluene-acetate, phase ratio = 0.25, repeat 3 case with location of detected interfaces.

Toluene-acetate – Phase ratio = 0.25, Average

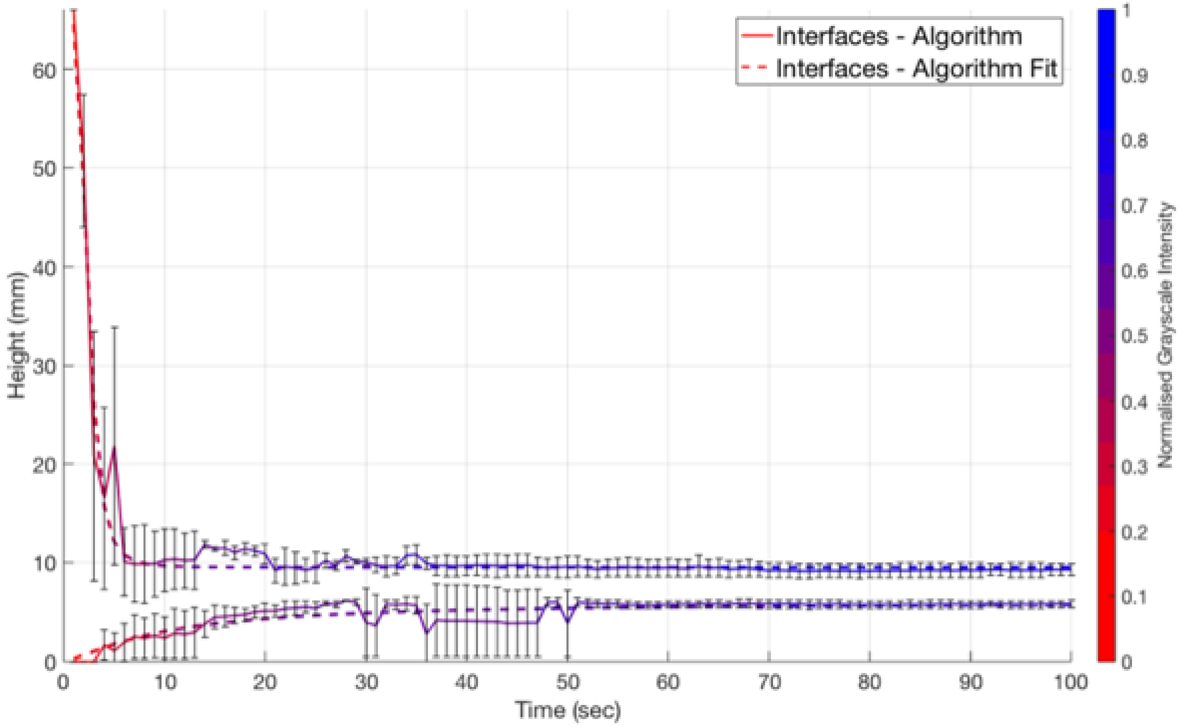

Figure S11: Averaged interface locations and normalised grayscale intensity over time for the toluene-acetate time series at a phase ratio of 0.25.

Toluene-deionised water – Phase ratio = 0.25, Repeat 1

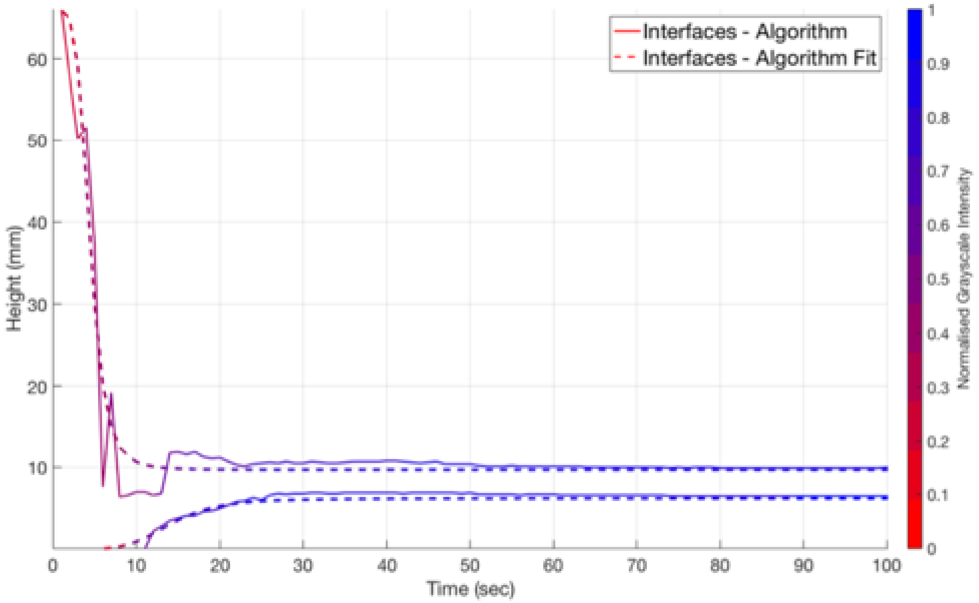

Figure S12: Detected interfaces and normalised grayscale intensity over time for repeat 1 of the toluene-deionised water time series at a phase ratio of 0.25.

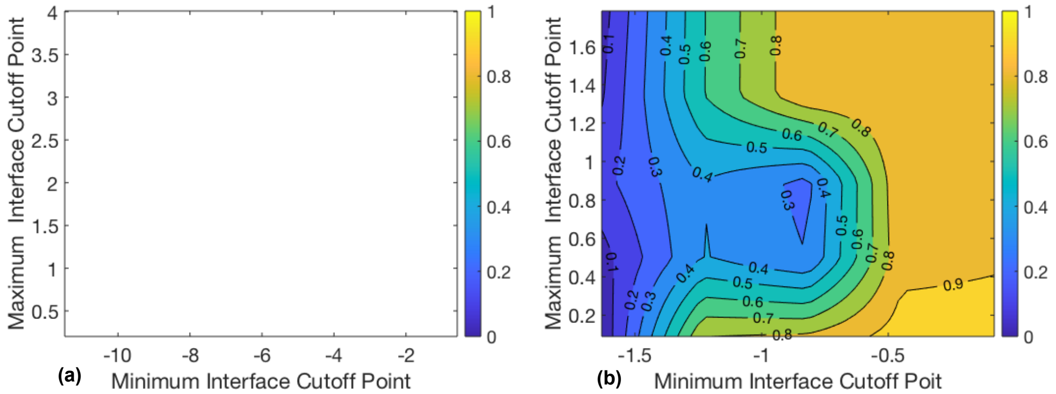

Figure S13: Contour plot of r^2 values for the sigmoidal curve fit for interface 1 data (a) and interface 2 data (b) depending on the maximum and minimum cut-off point combination – Toluene-deionised water, phase ratio = 0.25, repeat 1.

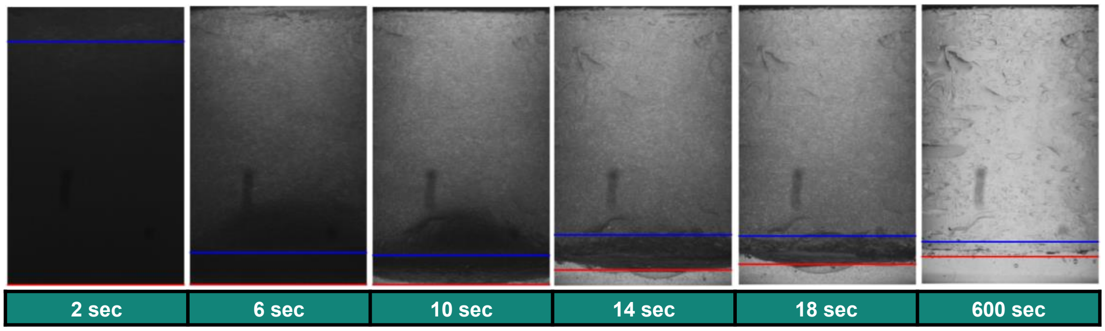

Figure S14: Sample images from toluene-deionised water, phase ratio = 0.25, repeat 1 case with location of detected interfaces.

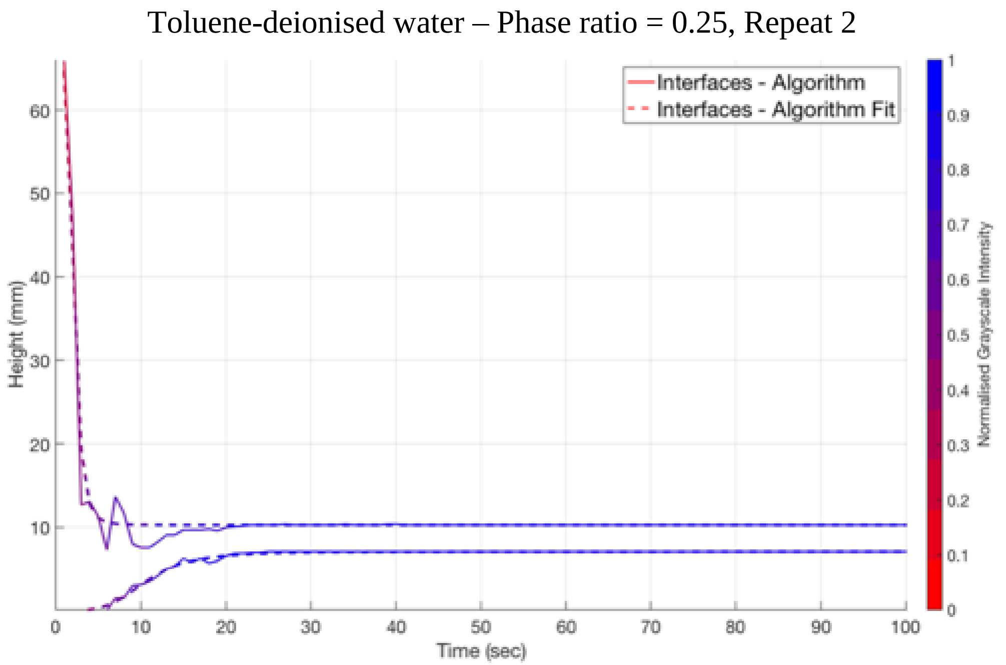

Figure S15: Detected interfaces and normalised grayscale intensity over time for repeat 2 of the toluene-deionised water time series at a phase ratio of 0.25.

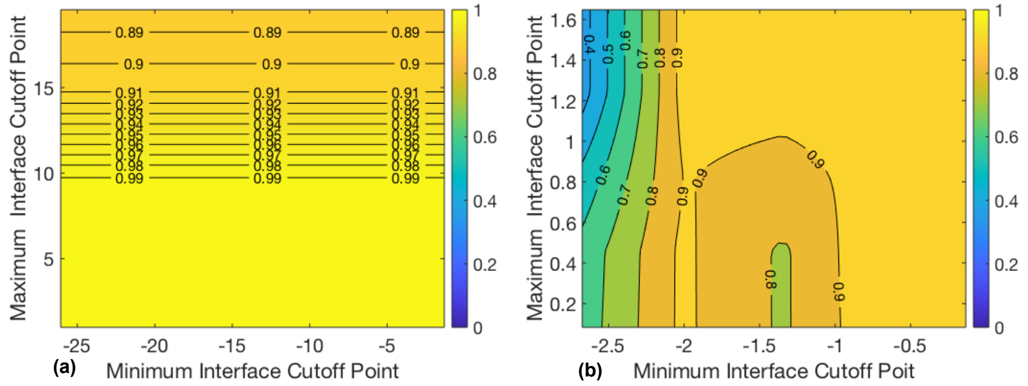

Figure S16: Contour plot of r^2 values for the sigmoidal curve fit for interface 1 data (a) and interface 2 data (b) depending on the maximum and minimum cut-off point combination – Toluene-deionised water, phase ratio = 0.25, repeat 2.

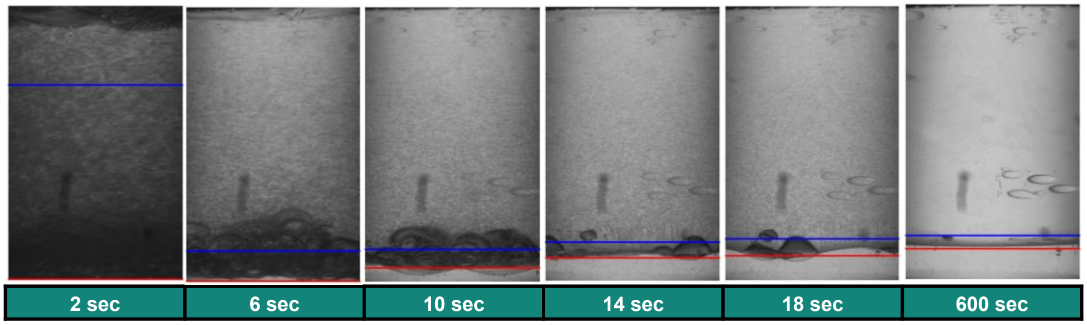

Figure S17: Sample images from toluene-deionised water, phase ratio = 0.25, repeat 2 case with location of detected interfaces.

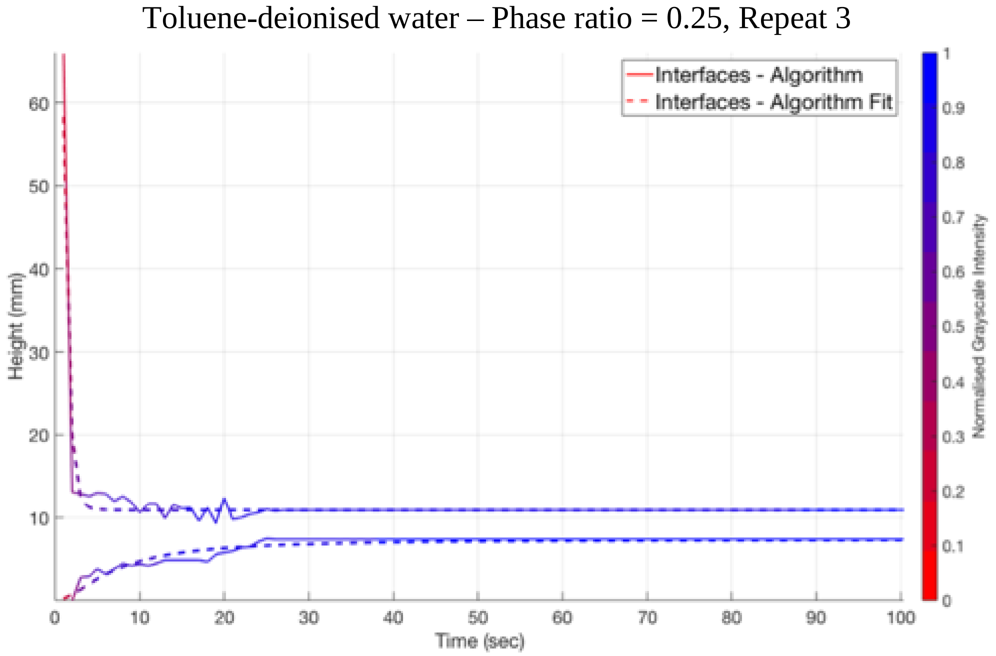

Figure S18: Detected interfaces and normalised grayscale intensity over time for repeat 3 of the toluene-deionised water time series at a phase ratio of 0.25.

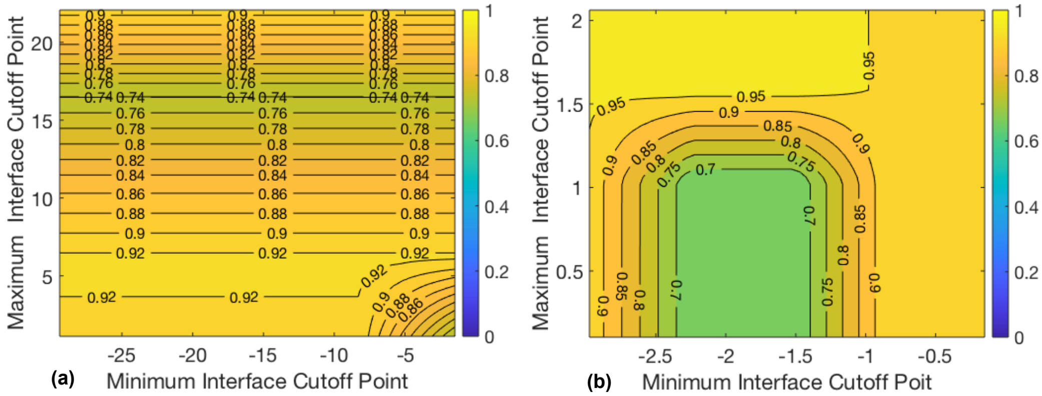

Figure S19: Contour plot of r^2 values for the sigmoidal curve fit for interface 1 data (a) and interface 2 data (b) depending on the maximum and minimum cut-off point combination – Toluene-deionised water, phase ratio = 0.25, repeat 3.

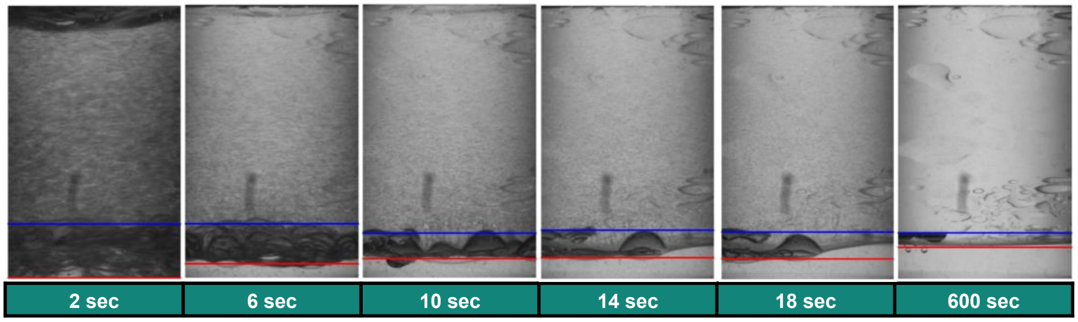

Figure S20: Sample images from toluene-deionised water, phase ratio = 0.25, repeat 3 case with location of detected interfaces.

Toluene-deionised water – Phase ratio = 0.25, Average

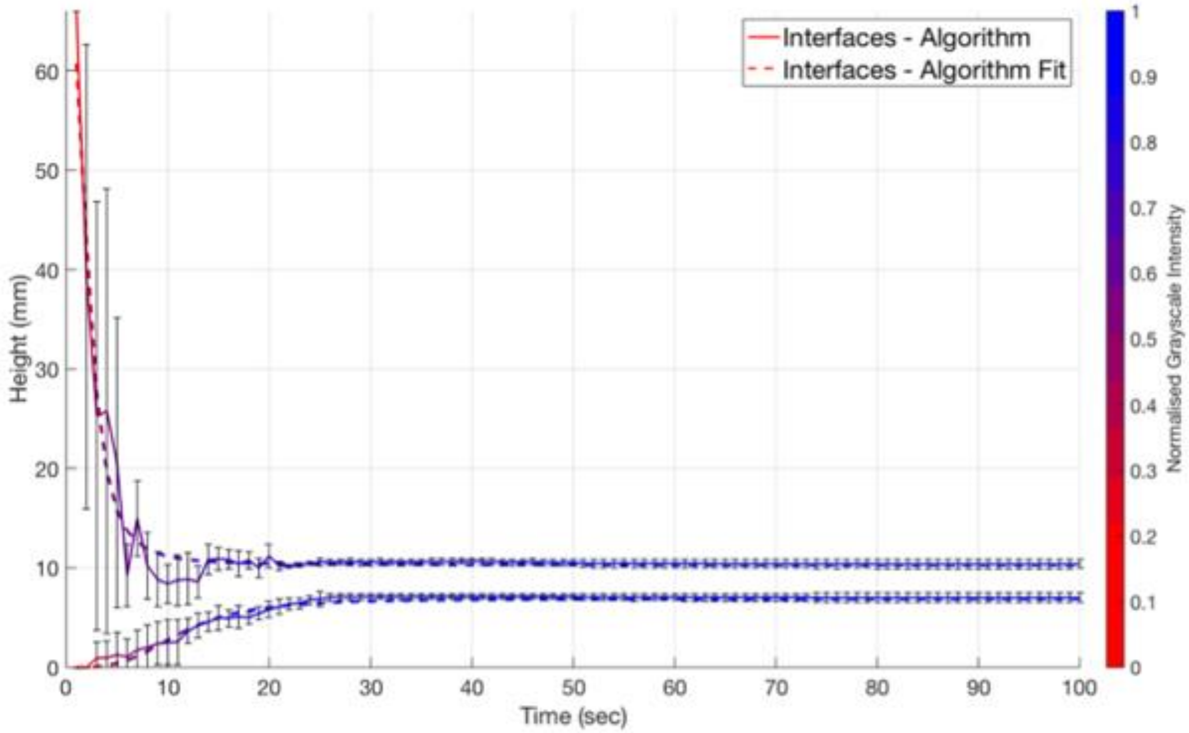

Figure S21: Averaged interface locations and normalised grayscale intensity over time for the toluene-deionised water time series at a phase ratio of 0.25.

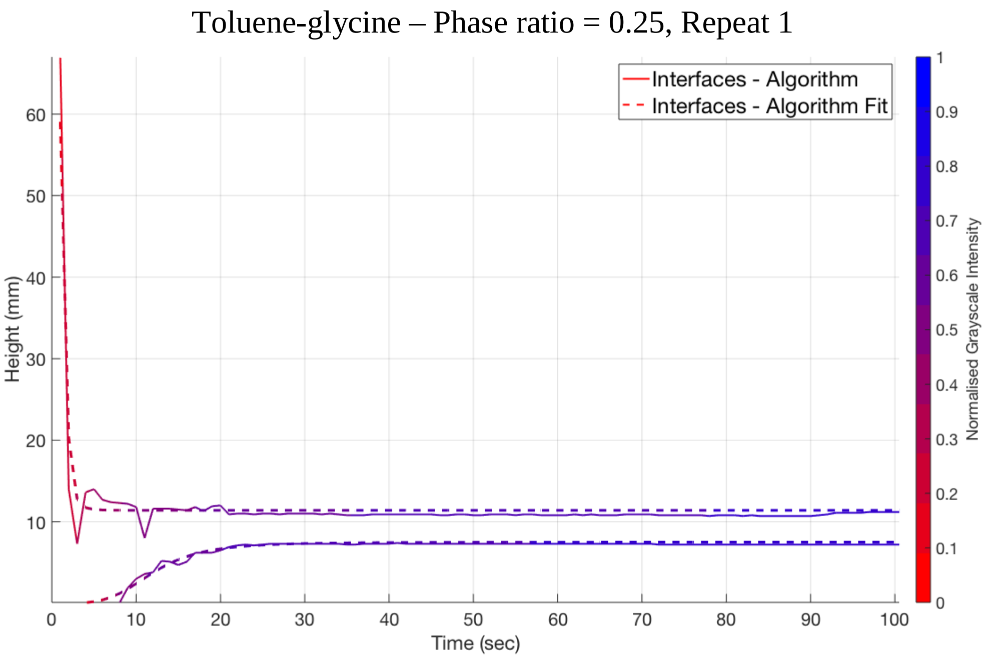

Figure S22: Detected interfaces and normalised grayscale intensity over time for repeat 1 of the toluene-glycine time series at a phase ratio of 0.25.

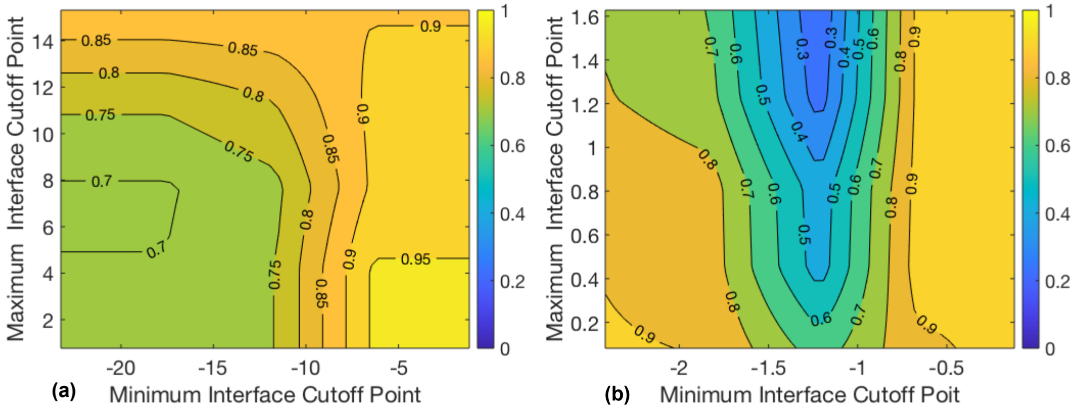

Figure S23: Contour plot of r^2 values for the sigmoidal curve fit for interface 1 data (a) and interface 2 data (b) depending on the maximum and minimum cut-off point combination – Toluene-glycine, phase ratio = 0.25, repeat 1.

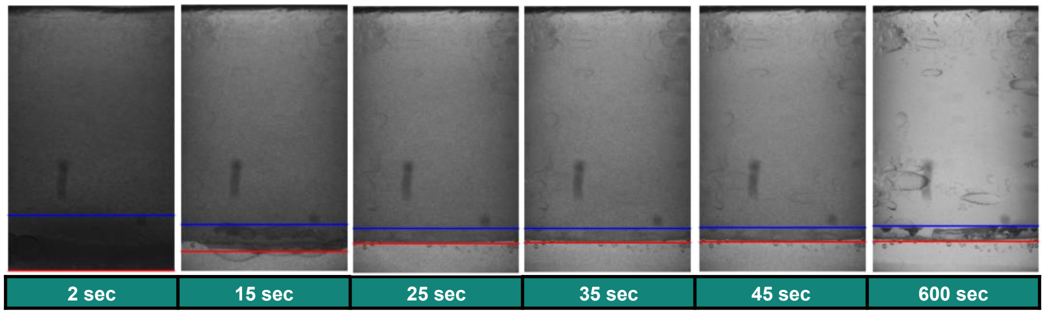

Figure S24: Sample images from toluene-glycine, phase ratio = 0.25, repeat 1 case with location of detected interfaces.

Toluene-glycine – Phase ratio = 0.25, Repeat 2

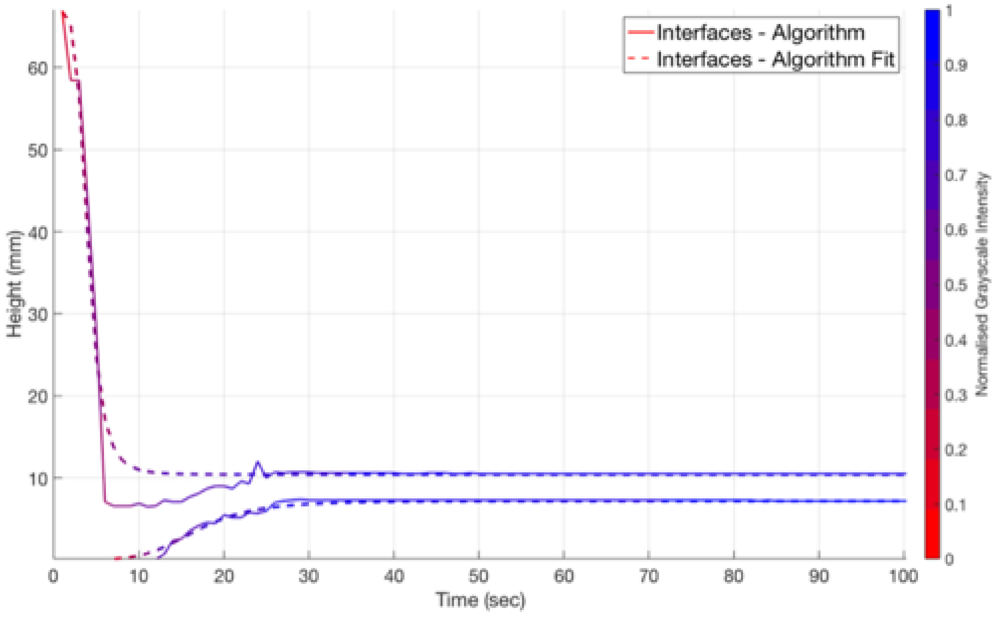

Figure S25: Detected interfaces and normalised grayscale intensity over time for repeat 2 of the toluene-glycine time series at a phase ratio of 0.25.

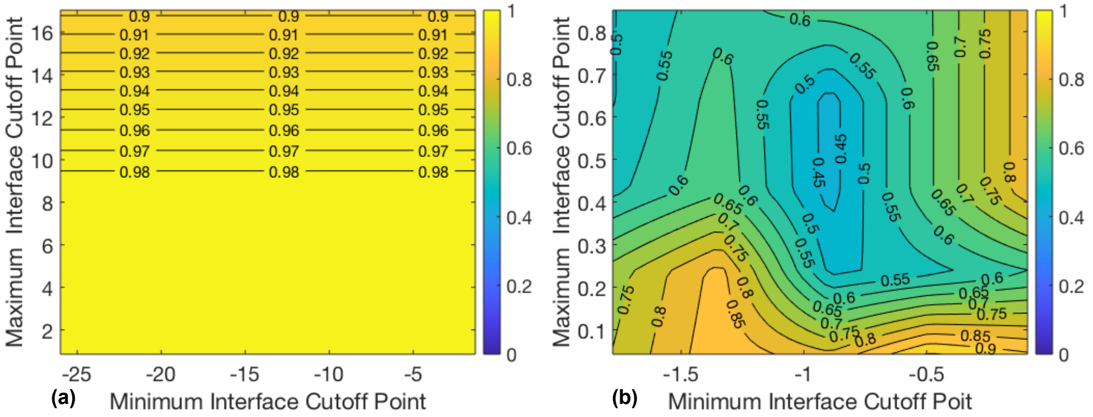

Figure S26: Contour plot of r^2 values for the sigmoidal curve fit for interface 1 data (a) and interface 2 data (b) depending on the maximum and minimum cut-off point combination – Toluene-glycine, phase ratio = 0.25, repeat 2.

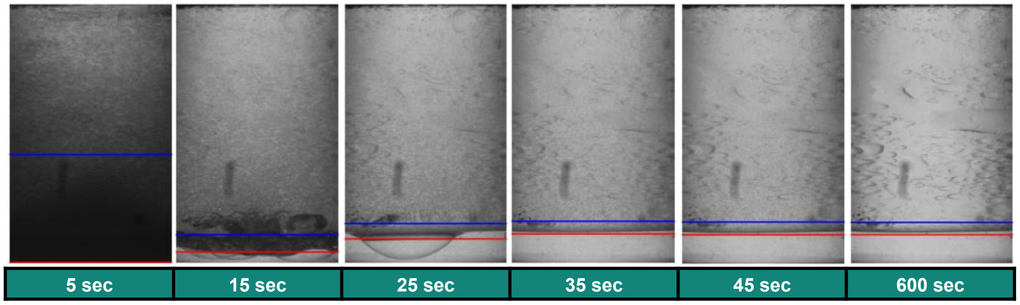

Figure S27: Sample images from toluene-glycine, phase ratio = 0.25, repeat 2 case with location of detected interfaces.

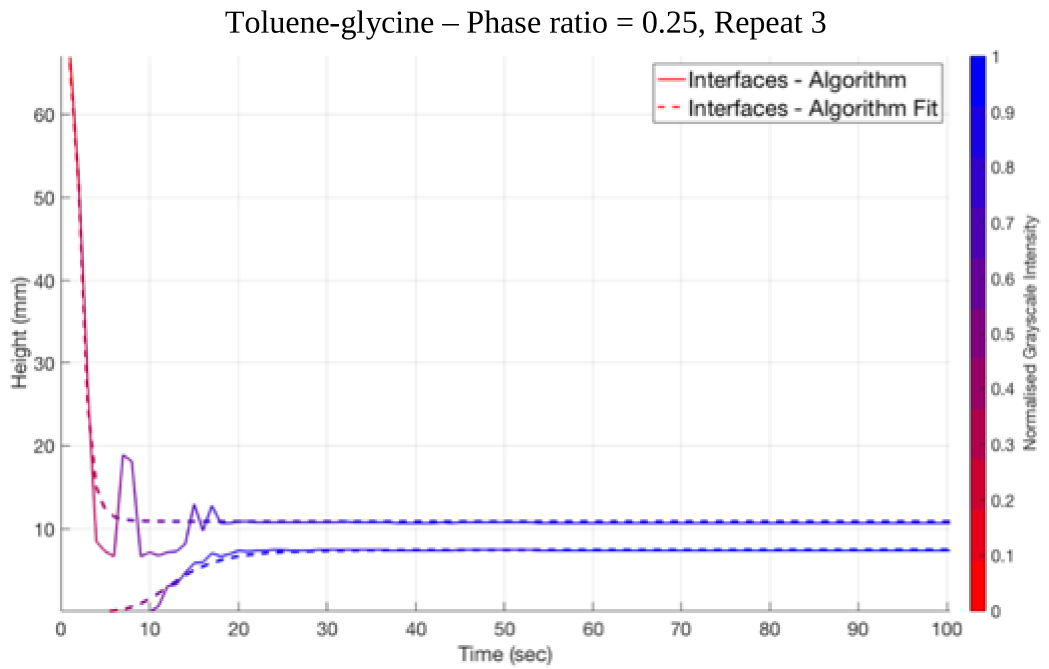

Figure S28: Detected interfaces and normalised grayscale intensity over time for repeat 3 of the toluene-glycine time series at a phase ratio of 0.25.

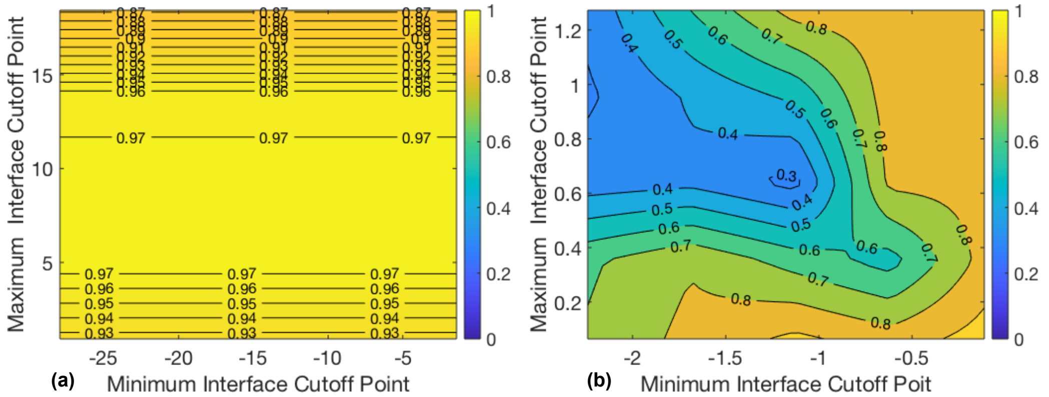

Figure S29: Contour plot of r^2 values for the sigmoidal curve fit for interface 1 data (a) and interface 2 data (b) depending on the maximum and minimum cut-off point combination – Toluene-glycine, phase ratio = 0.25, repeat 3.

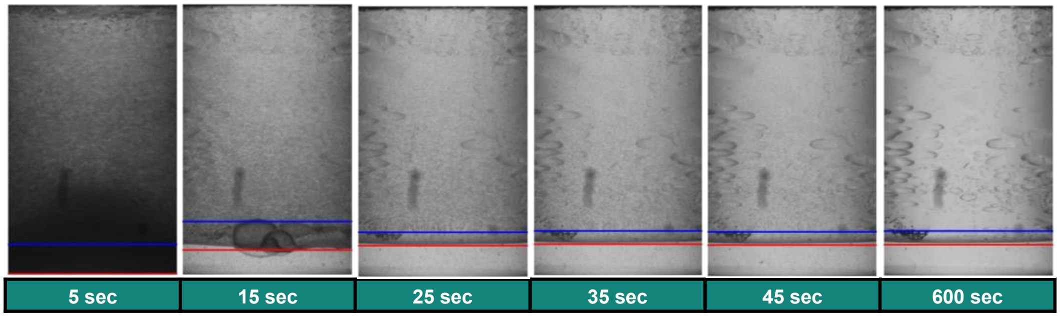

Figure S30: Sample images from toluene-glycine, phase ratio = 0.25, repeat 3 case with location of detected interfaces.

Toluene-glycine – Phase ratio = 0.25, Average

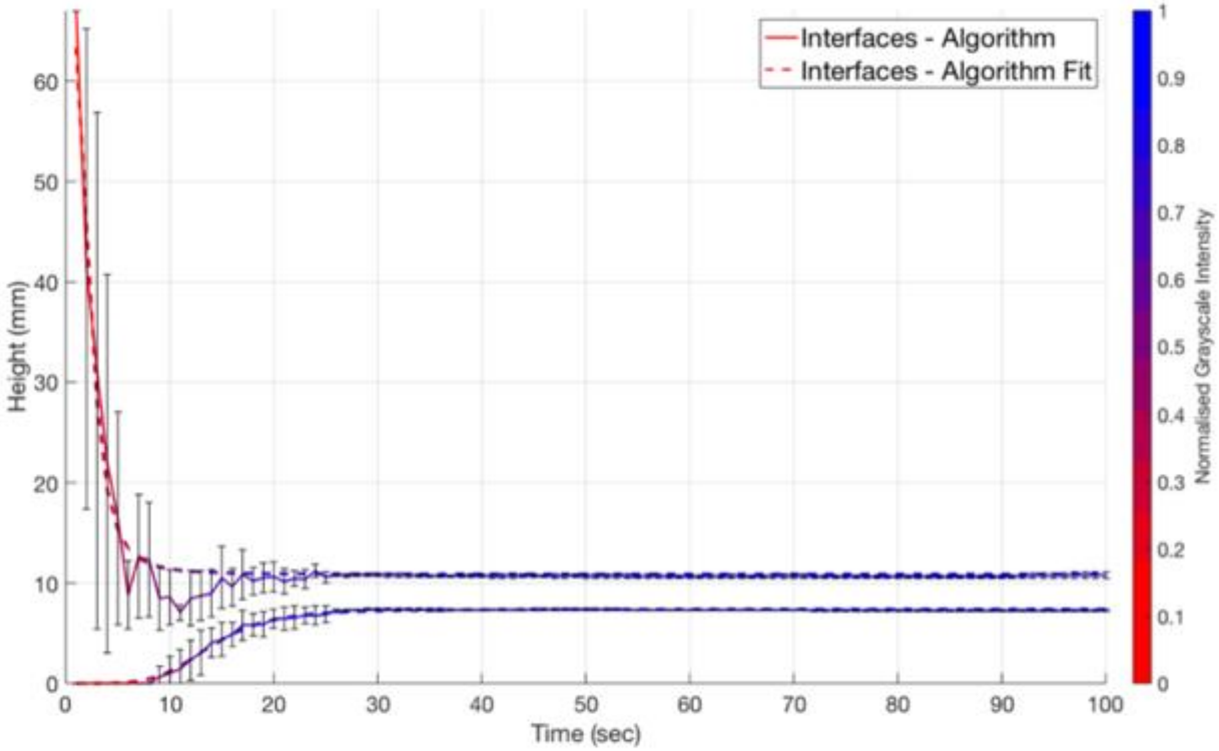

Figure S31: Averaged interface locations and normalised grayscale intensity over time for the toluene-glycine time series at a phase ratio of 0.25.

Toluene-acetate – Phase ratio = 1, Repeat 1

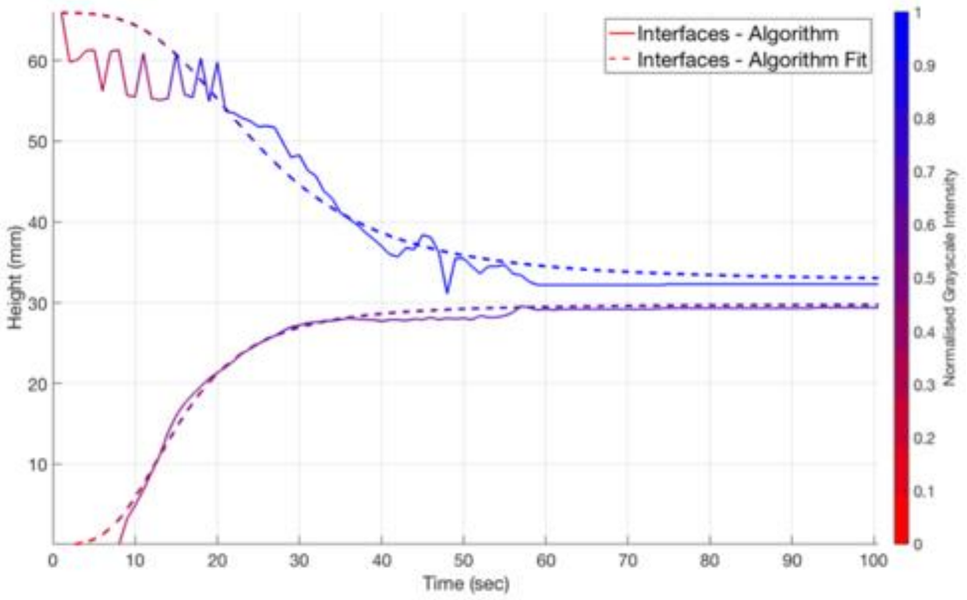

Figure S32: Detected interfaces and normalised grayscale intensity over time for repeat 1 of the toluene-acetate time series at a phase ratio of 1.

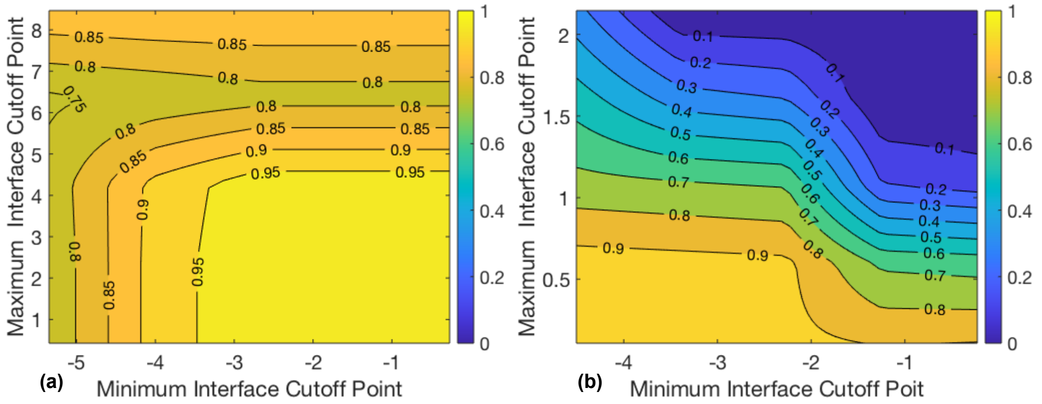

Figure S33: Contour plot of r^2 values for the sigmoidal curve fit for interface 1 data (a) and interface 2 data (b) depending on the maximum and minimum cut-off point combination – Toluene-acetate, phase ratio = 1, repeat 1.

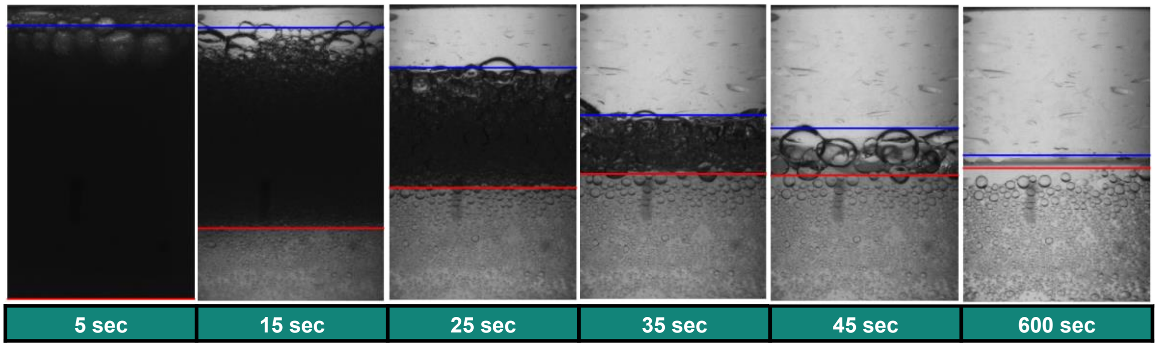

Figure S34: Sample images from toluene-acetate, phase ratio = 1, repeat 1 case with location of detected interfaces.

Toluene-acetate – Phase ratio = 1, Repeat 2

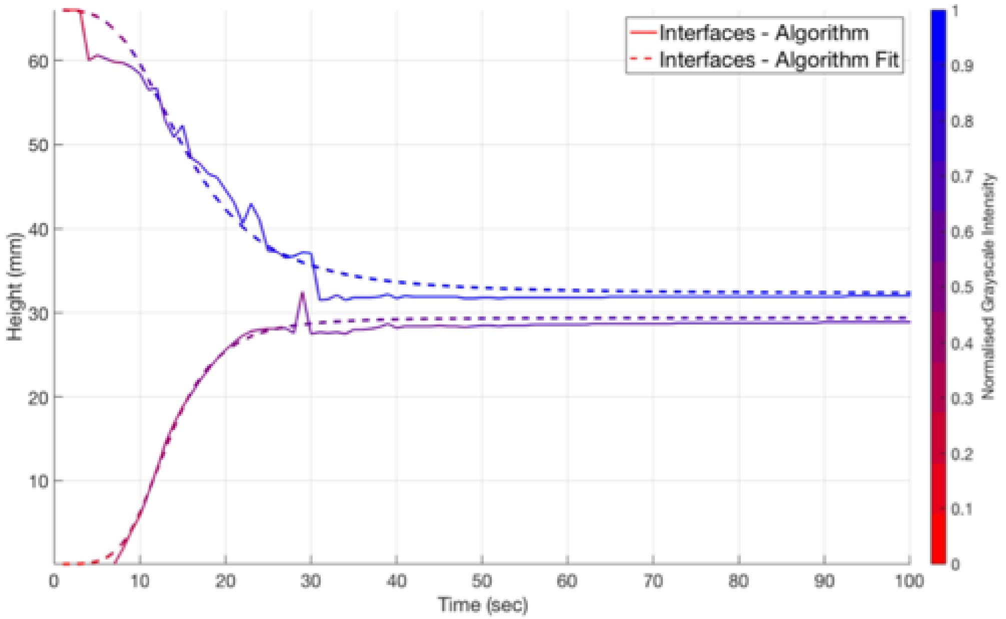

Figure S35: Detected interfaces and normalised grayscale intensity over time for repeat 2 of the toluene-acetate time series at a phase ratio of 1.

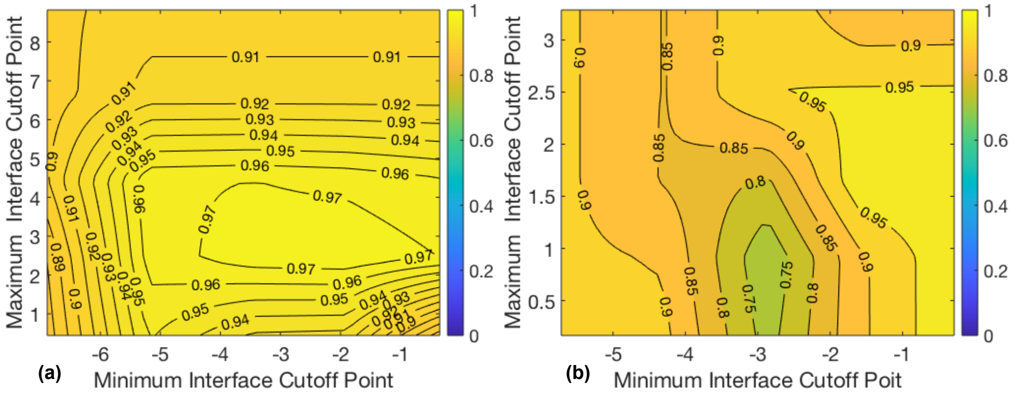

Figure S36: Contour plot of r^2 values for the sigmoidal curve fit for interface 1 data (a) and interface 2 data (b) depending on the maximum and minimum cut-off point combination – Toluene-acetate, phase ratio = 1, repeat 2.

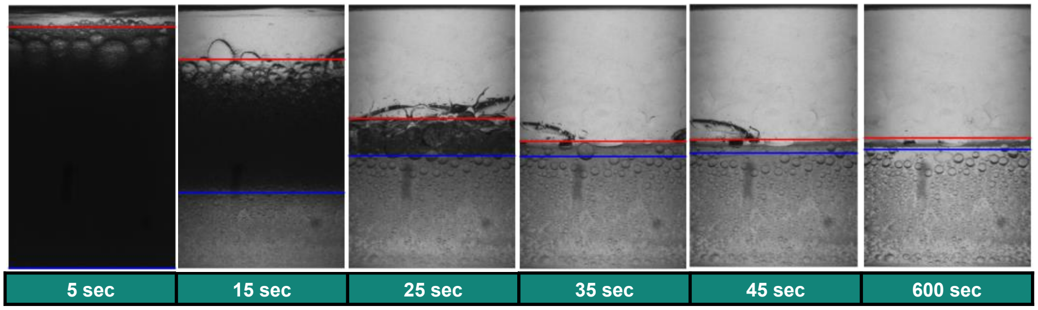

Figure S37: Sample images from toluene-acetate, phase ratio = 1, repeat 2 case with location of detected interfaces.

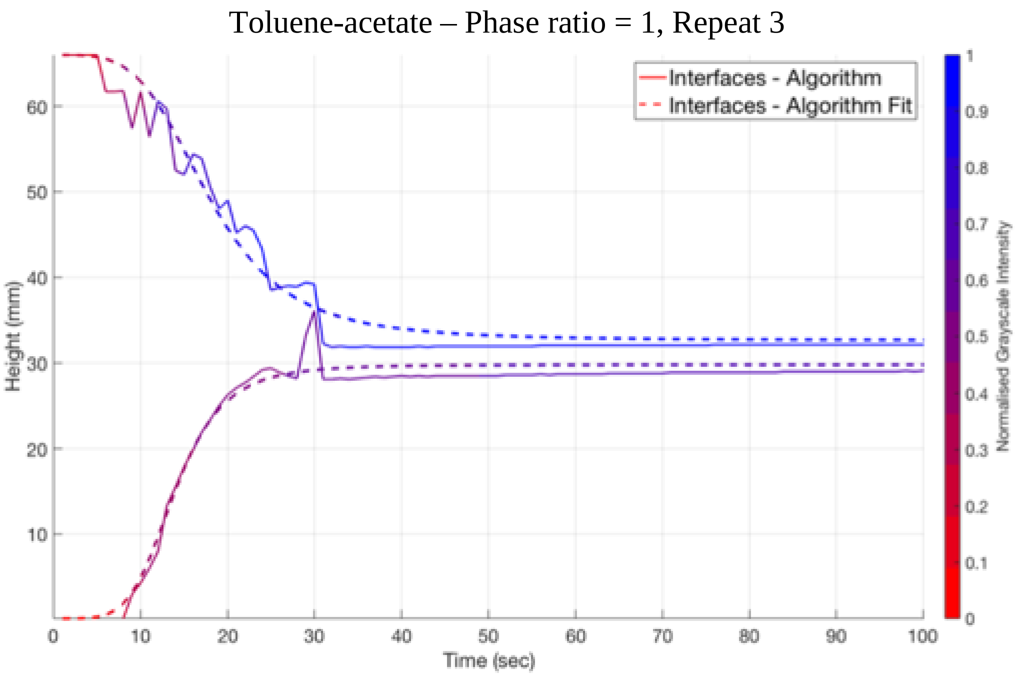

Figure S38: Detected interfaces and normalised grayscale intensity over time for repeat 3 of the toluene-acetate time series at a phase ratio of 1.

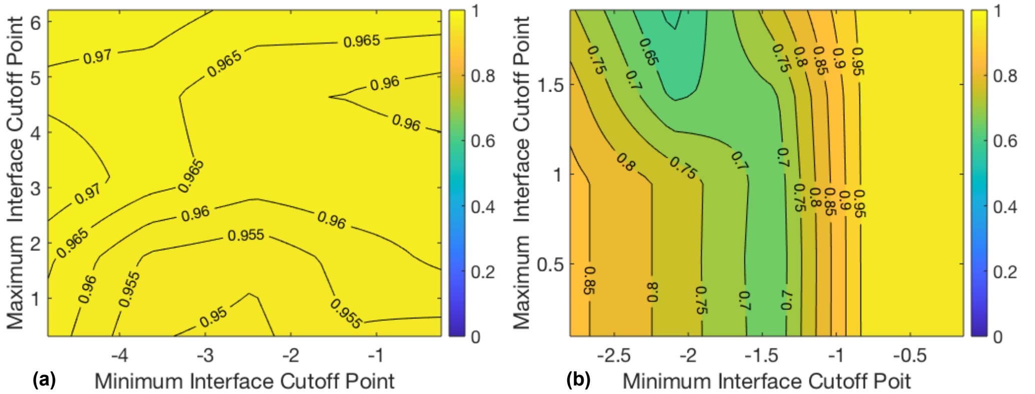

Figure S39: Contour plot of r^2 values for the sigmoidal curve fit for interface 1 data (a) and interface 2 data (b) depending on the maximum and minimum cut-off point combination – Toluene-acetate, phase ratio = 1, repeat 3.

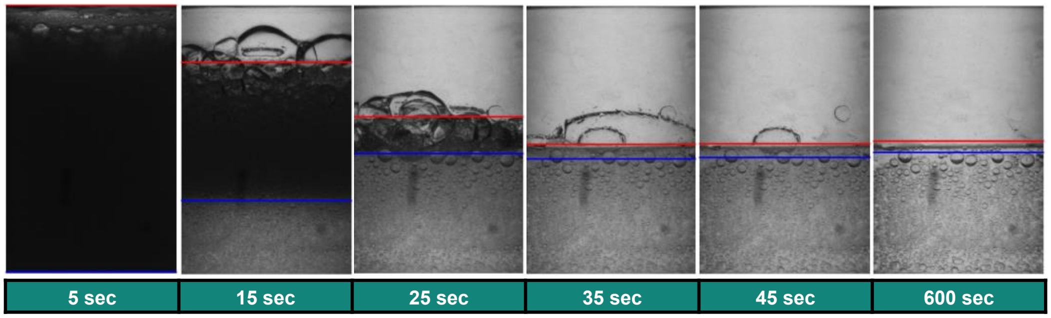

Figure S40: Sample images from toluene-acetate, phase ratio = 1, repeat 3 case with location of detected interfaces.

Toluene-acetate – Phase ratio = 1, Average

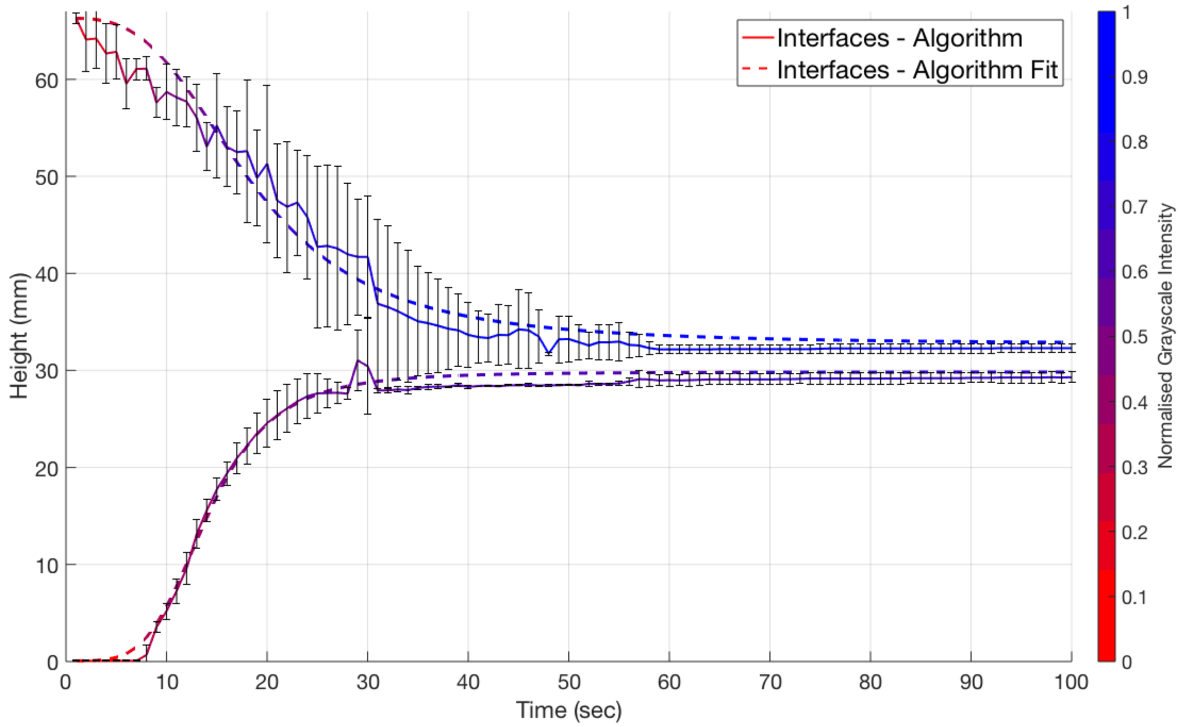

Figure S41: Averaged interface locations and normalised grayscale intensity over time for the toluene-acetate time series at a phase ratio of 1.

Toluene-deionised water – Phase ratio = 1, Repeat 1

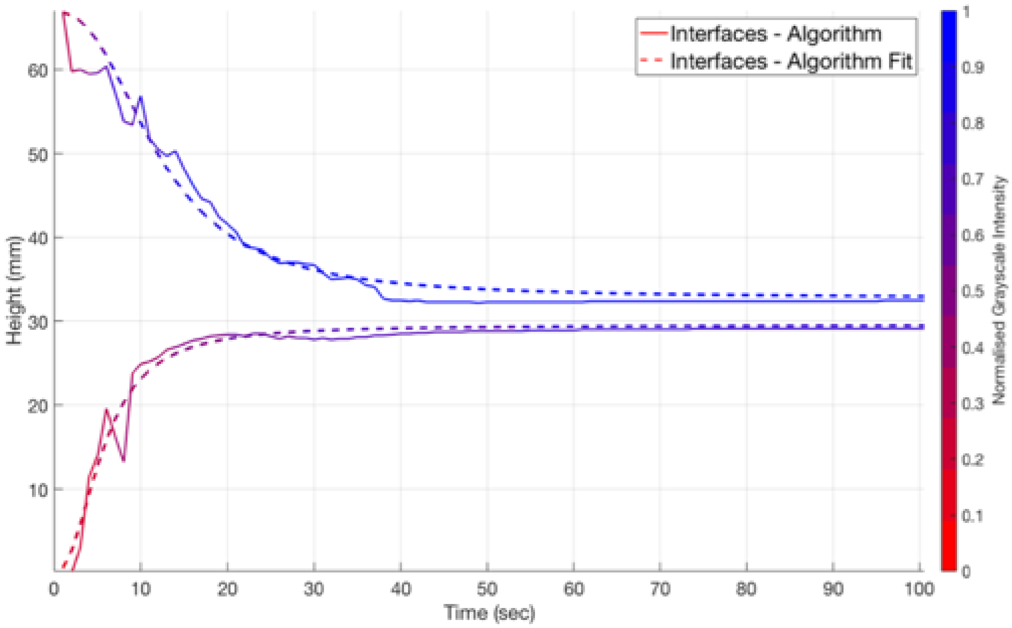

Figure S42: Detected interfaces and normalised grayscale intensity over time for repeat 1 of the toluene-deionised water time series at a phase ratio of 1.

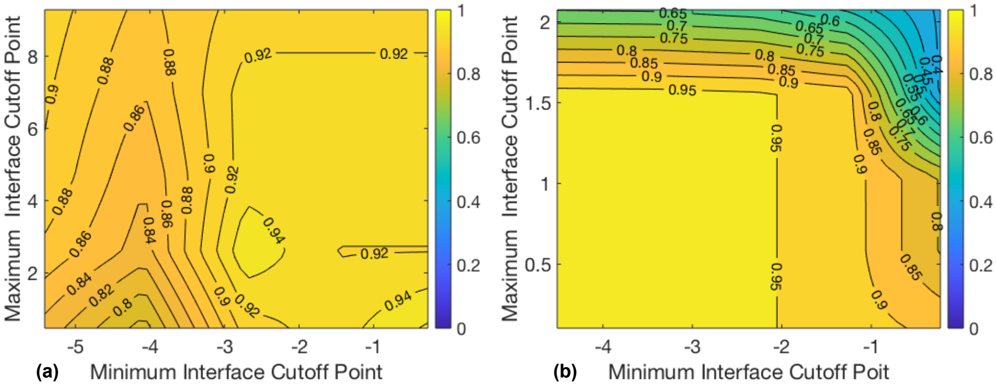

Figure S43: Contour plot of r^2 values for the sigmoidal curve fit for interface 1 data (a) and interface 2 data (b) depending on the maximum and minimum cut-off point combination – Toluene-deionised water, phase ratio = 1, repeat 1.

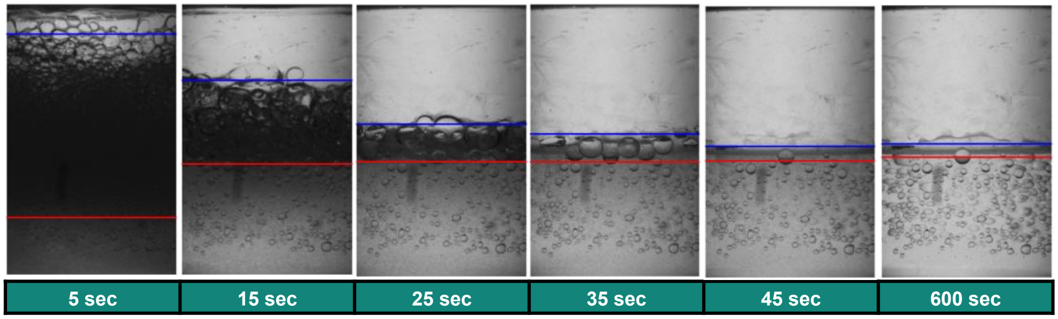

Figure S44: Sample images from toluene-deionised water, phase ratio = 1, repeat 1 case with location of detected interfaces.

Toluene-deionised water – Phase ratio = 1, Repeat 2

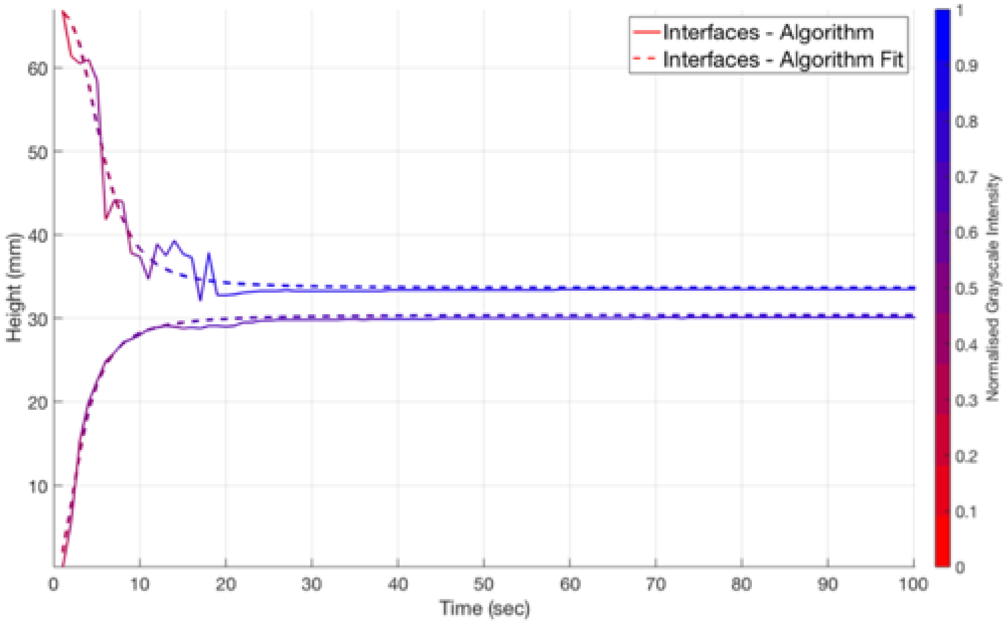

Figure S45: Detected interfaces and normalised grayscale intensity over time for repeat 1 of the toluene-deionised water time series at a phase ratio of 2.

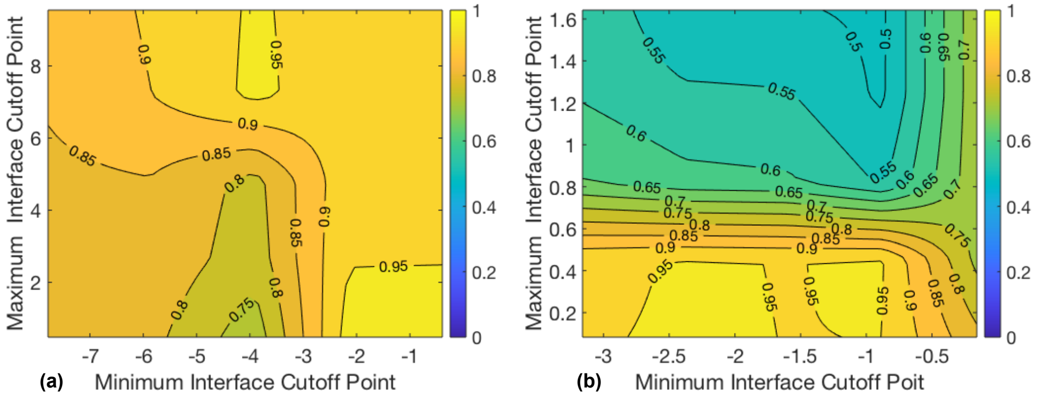

Figure S46: Contour plot of r^2 values for the sigmoidal curve fit for interface 1 data (a) and interface 2 data (b) depending on the maximum and minimum cut-off point combination – Toluene-deionised water, phase ratio = 1, repeat 2.

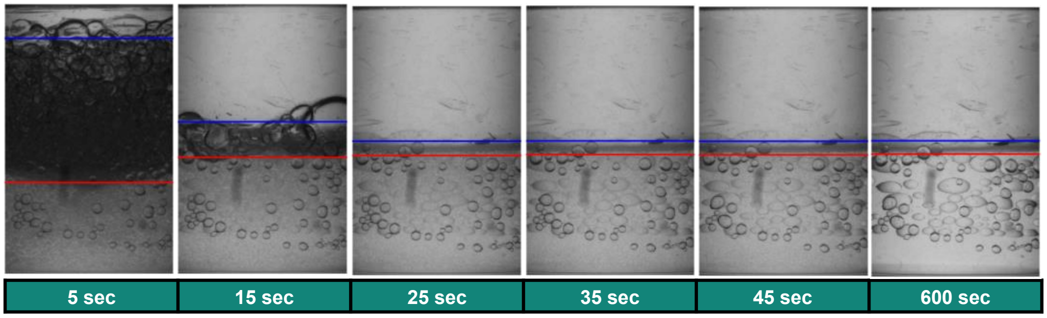

Figure S47: Sample images from toluene-deionised water, phase ratio = 1, repeat 2 case with location of detected interfaces.

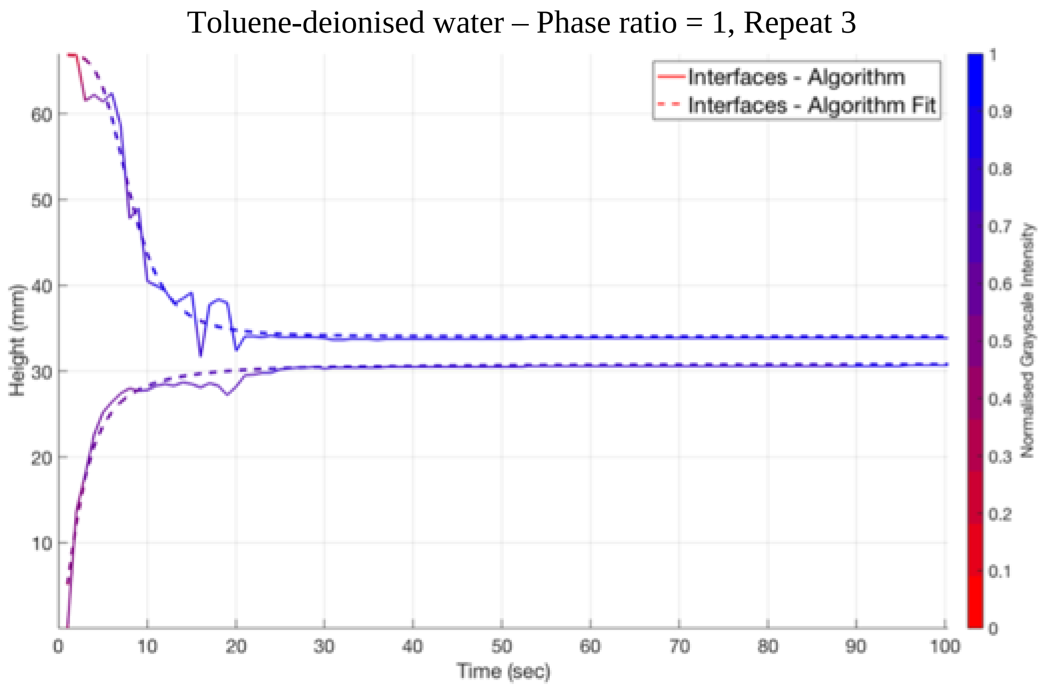

Figure S48: Detected interfaces and normalised grayscale intensity over time for repeat 3 of the toluene-deionised water time series at a phase ratio of 1.

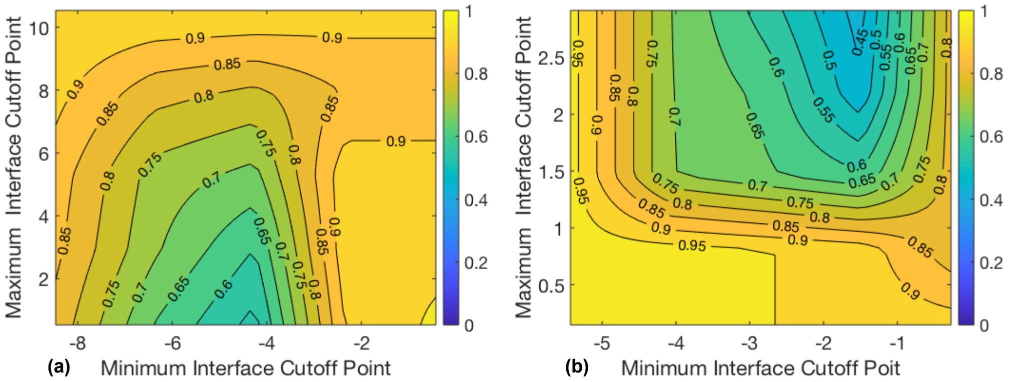

Figure S49: Contour plot of r^2 values for the sigmoidal curve fit for interface 1 data (a) and interface 2 data (b) depending on the maximum and minimum cut-off point combination – Toluene-deionised water, phase ratio = 1, repeat 3.

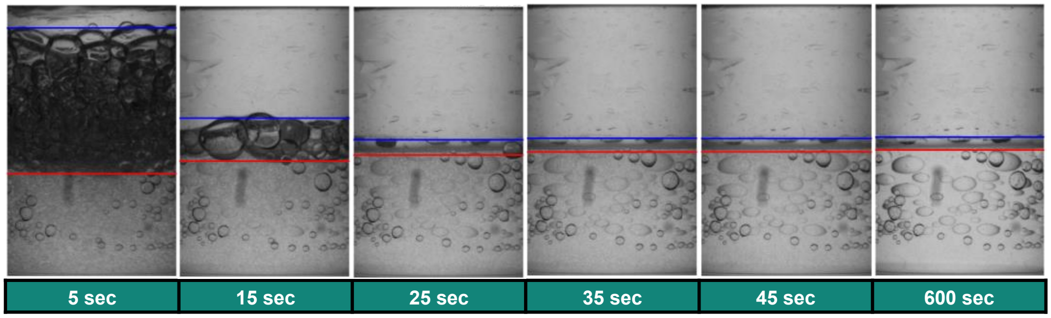

Figure S50: Sample images from toluene-deionised water, phase ratio = 1, repeat 3 case with location of detected interfaces.

Toluene-deionised water– Phase ratio = 1, Average

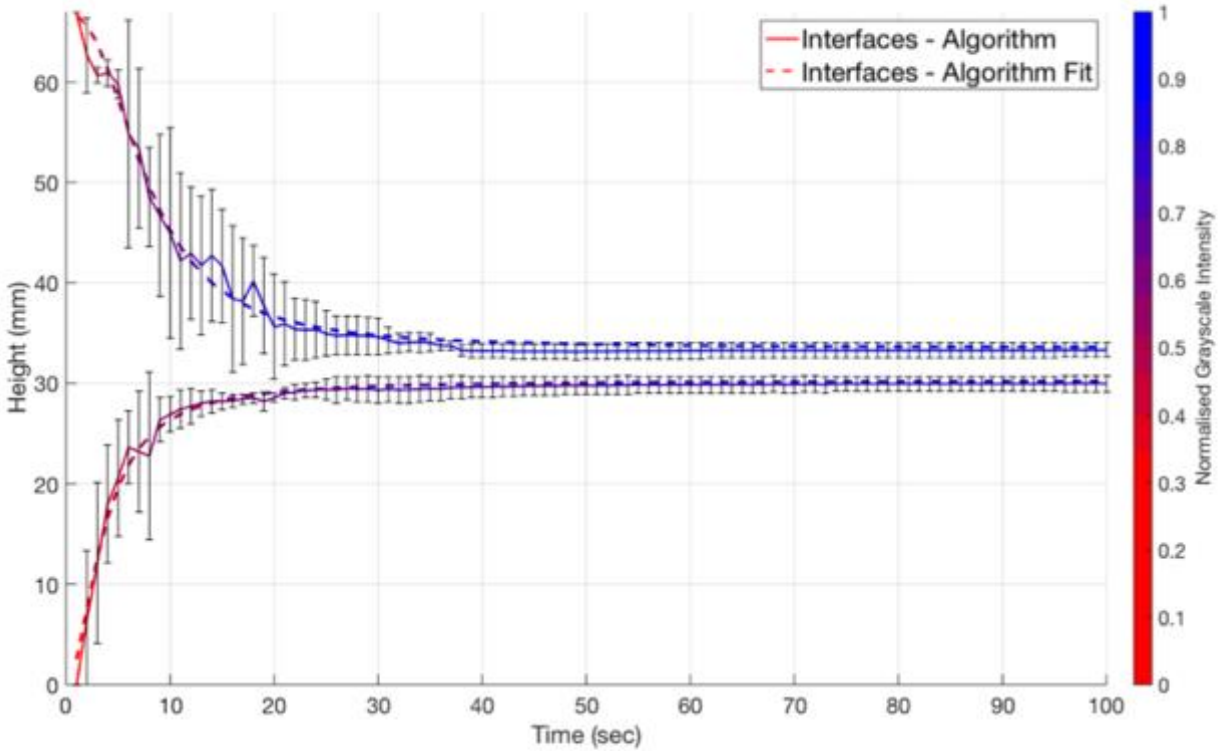

Figure S51: Averaged interface locations and normalised grayscale intensity over time for the toluene-deionised water time series at a phase ratio of 1.

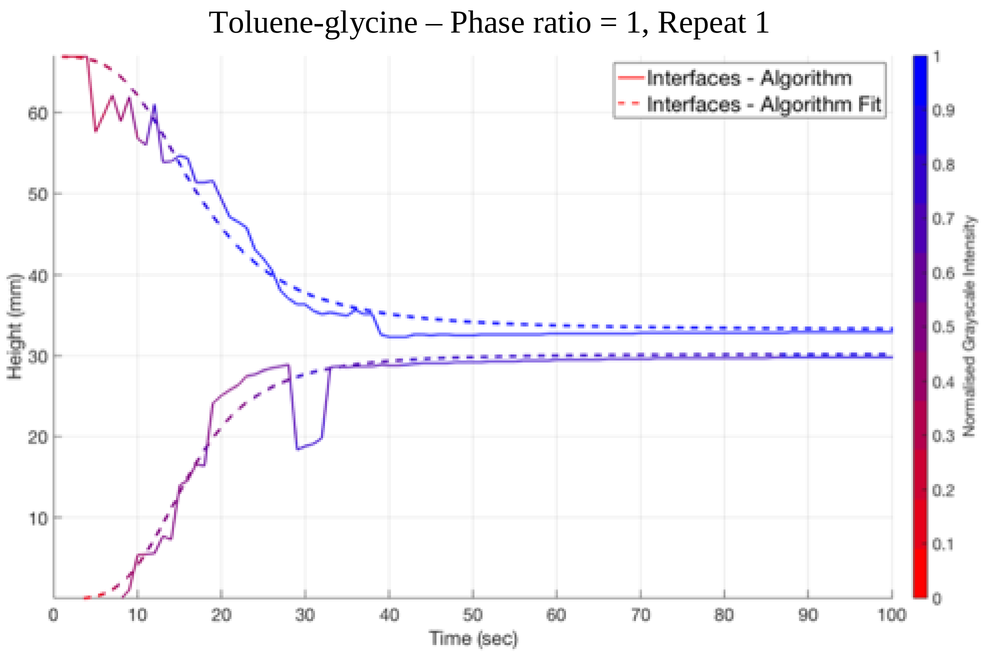

Figure S52: Detected interfaces and normalised grayscale intensity over time for repeat 1 of the toluene-glycine time series at a phase ratio of 1.

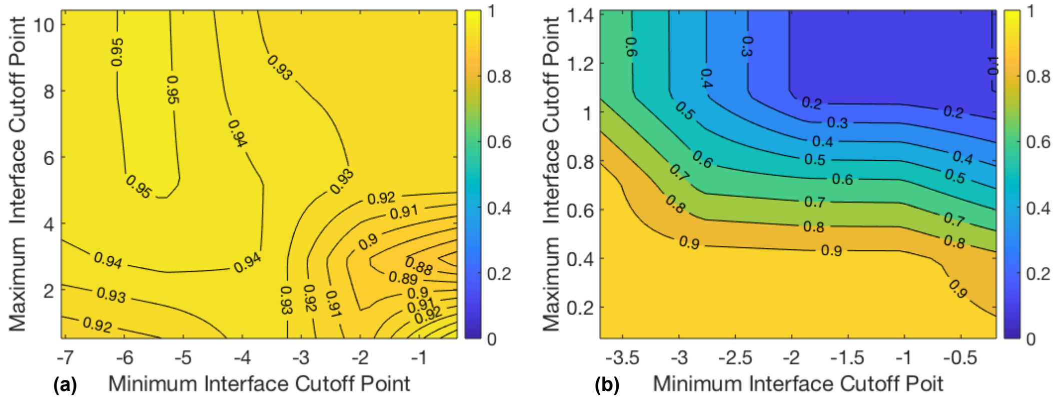

Figure S53: Contour plot of r^2 values for the sigmoidal curve fit for interface 1 data (a) and interface 2 data (b) depending on the maximum and minimum cut-off point combination – Toluene-glycine, phase ratio = 1, repeat 1.

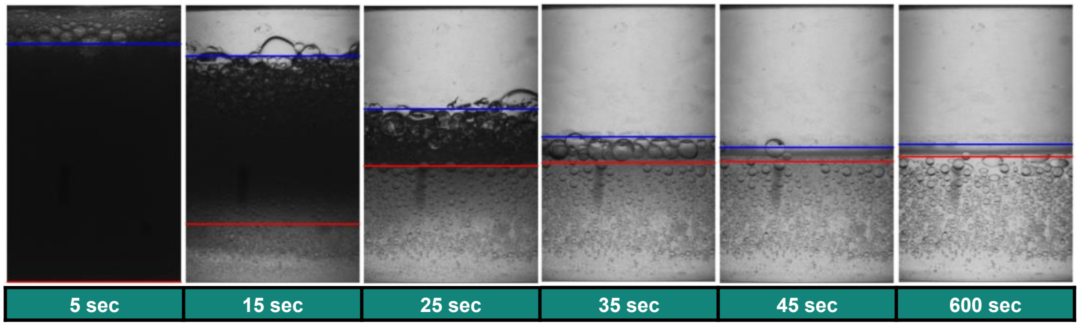

Figure S54: Sample images from toluene-glycine, phase ratio = 1, repeat 1 case with location of detected interfaces.

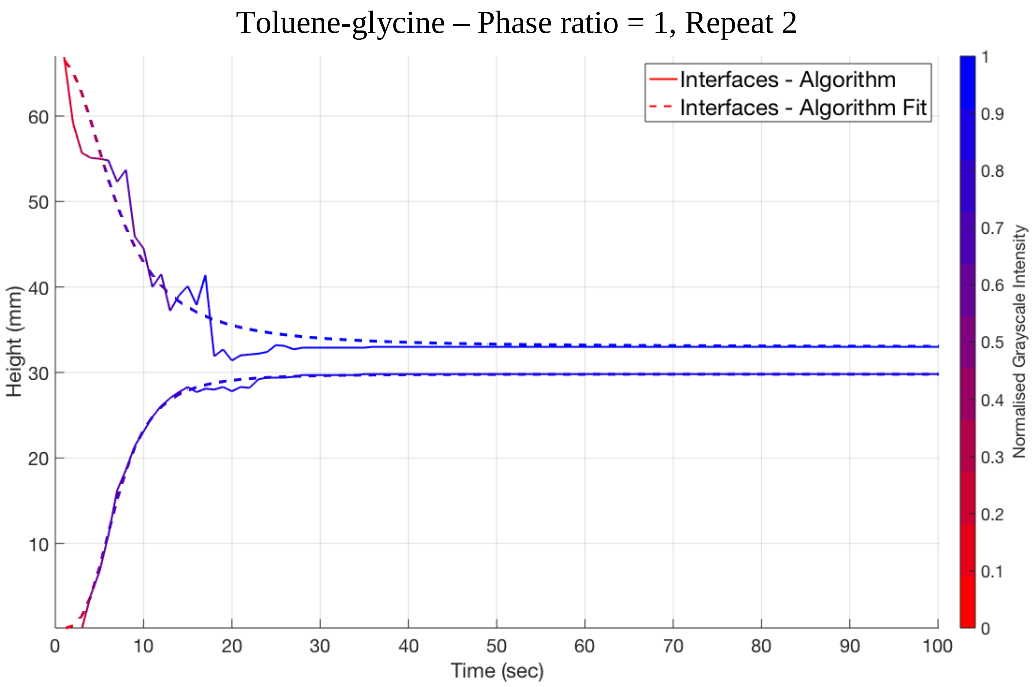

Figure S55: Detected interfaces and normalised grayscale intensity over time for repeat 2 of the toluene-glycine time series at a phase ratio of 1.

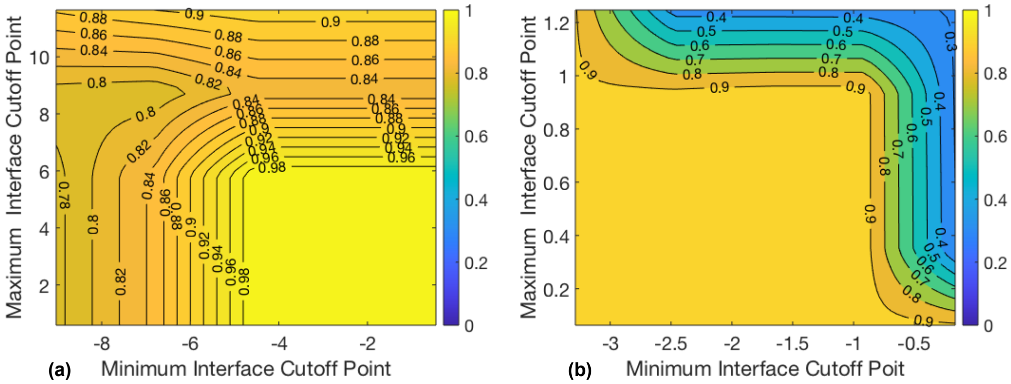

Figure S56: Contour plot of r^2 values for the sigmoidal curve fit for interface 1 data (a) and interface 2 data (b) depending on the maximum and minimum cut-off point combination – Toluene-glycine, phase ratio = 1, repeat 2.

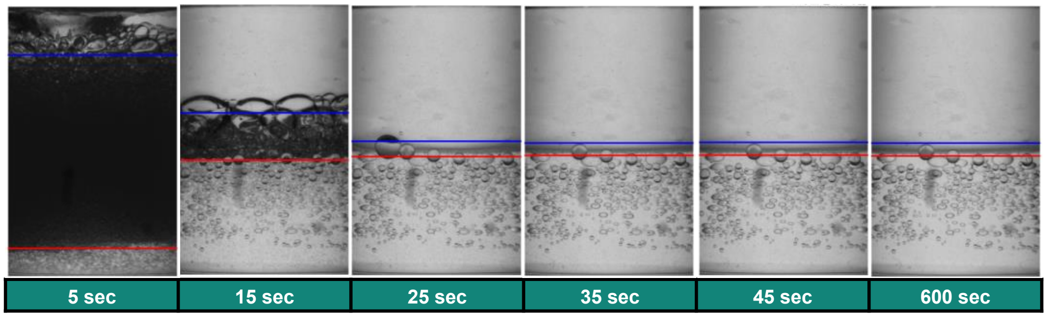

Figure S57: Sample images from toluene-glycine, phase ratio = 1, repeat 2 case with location of detected interfaces.

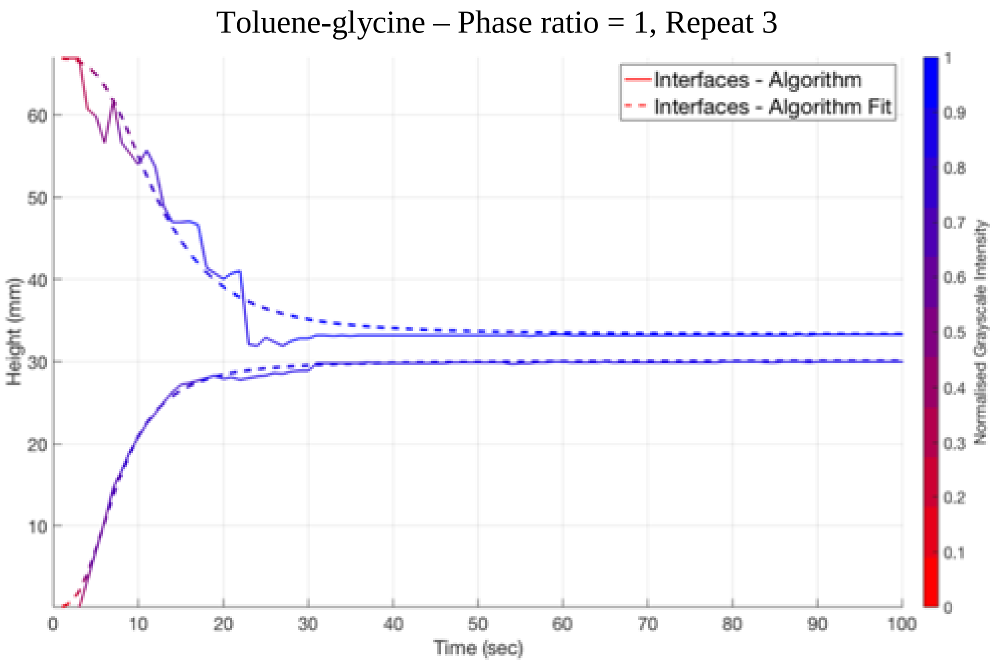

Figure S58: Detected interfaces and normalised grayscale intensity over time for repeat 3 of the toluene-glycine time series at a phase ratio of 1.

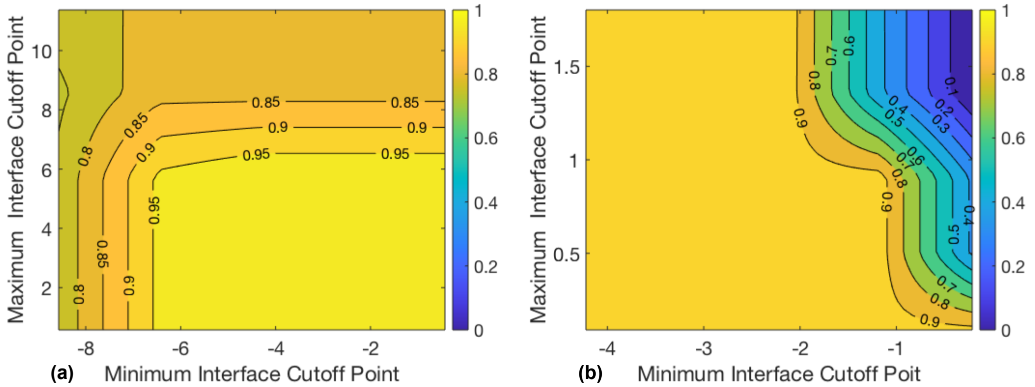

Figure S59: Contour plot of r^2 values for the sigmoidal curve fit for interface 1 data (a) and interface 2 data (b) depending on the maximum and minimum cut-off point combination – Toluene-glycine, phase ratio = 1, repeat 3.

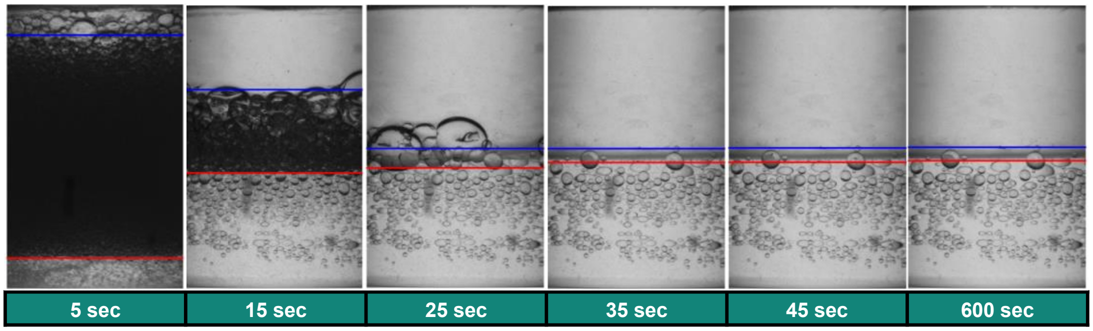

Figure S60: Sample images from toluene-glycine, phase ratio = 1, repeat 3 case with location of detected interfaces.

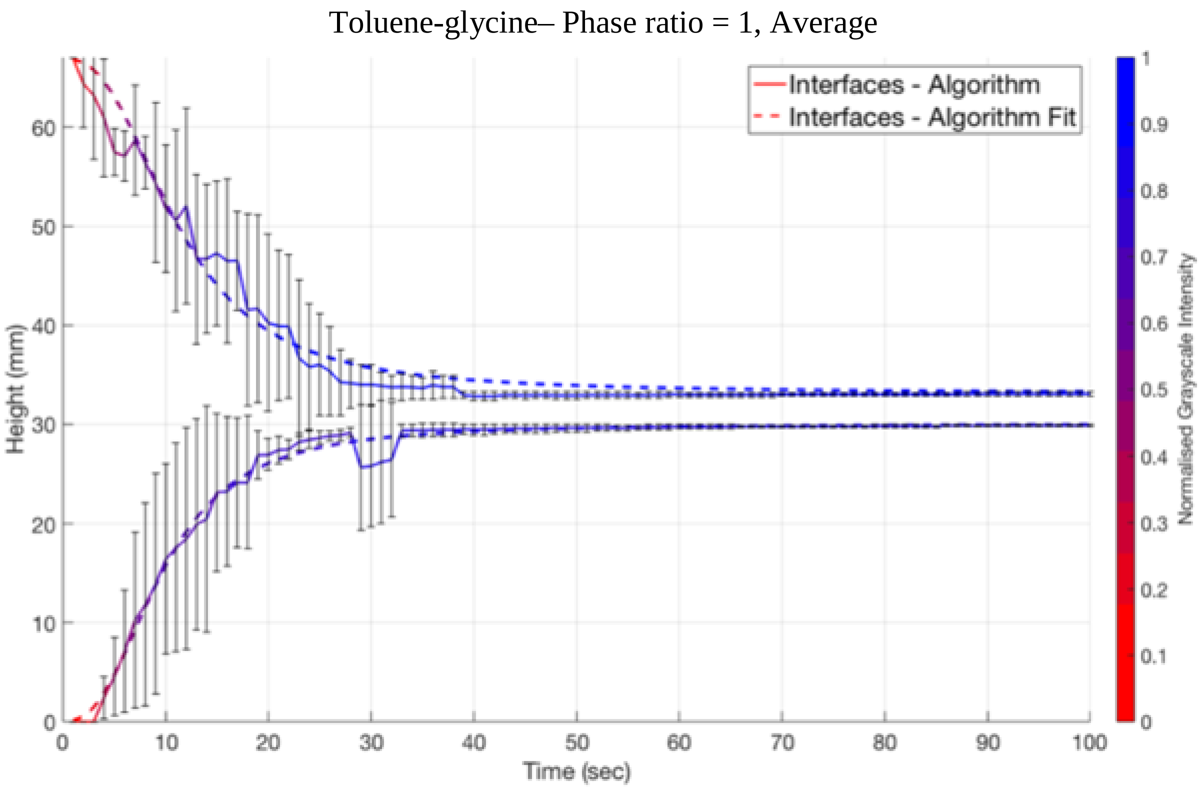

Figure S61: Averaged interface locations and normalised grayscale intensity over time for the toluene-glycine time series at a phase ratio of 1.

Toluene-acetate – Phase ratio = 4, Repeat 1

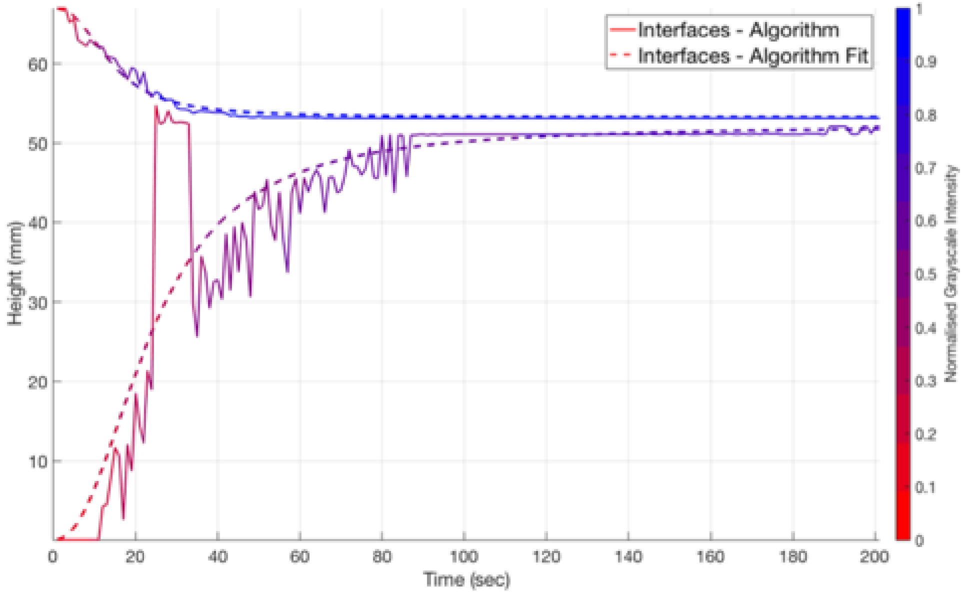

Figure S62: Detected interfaces and normalised grayscale intensity over time for repeat 1 of the toluene-acetate time series at a phase ratio of 4.

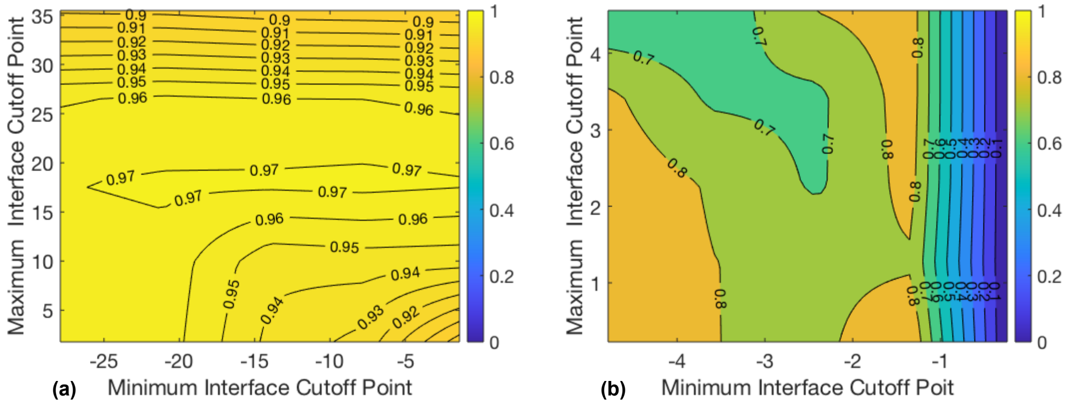

Figure S63: Contour plot of r^2 values for the sigmoidal curve fit for interface 1 data (a) and interface 2 data (b) depending on the maximum and minimum cut-off point combination – Toluene-acetate, phase ratio = 4, repeat 1.

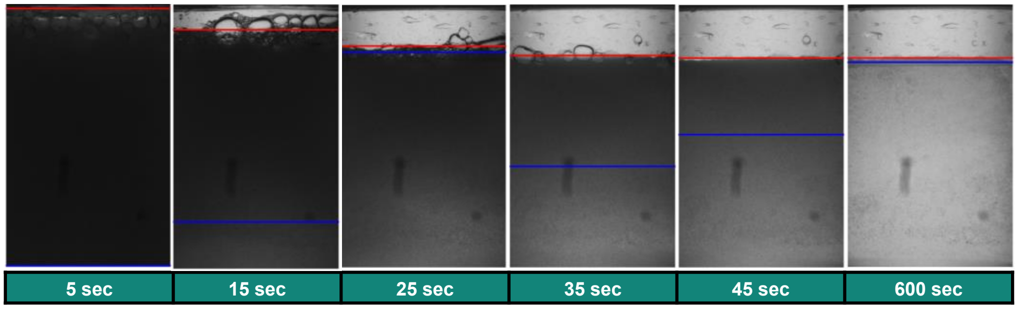

Figure S64: Sample images from toluene-acetate, phase ratio = 4, repeat 1 case with location of detected interfaces.

Toluene-acetate – Phase ratio = 4, Repeat 2

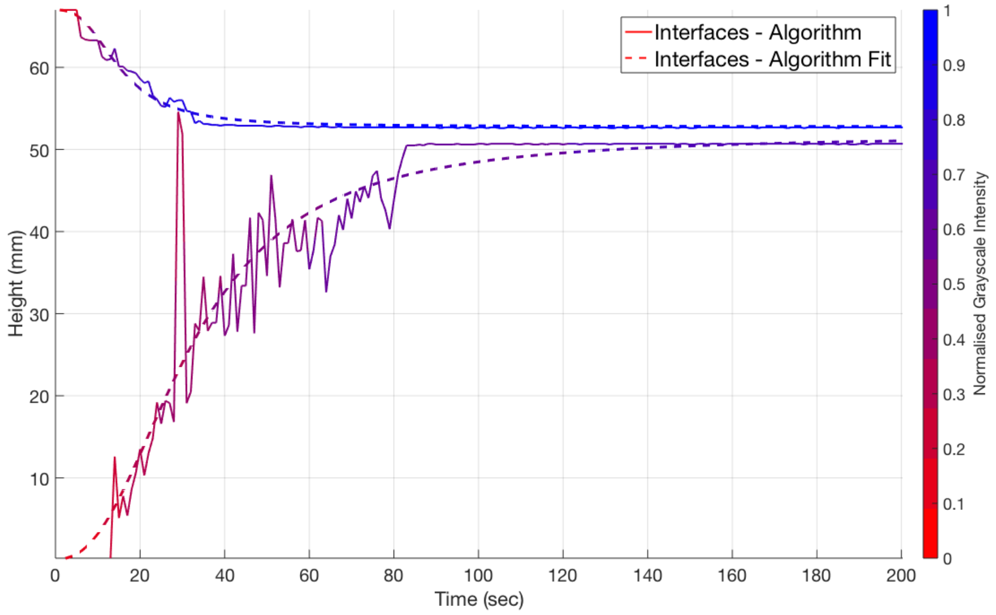

Figure S65: Detected interfaces and normalised grayscale intensity over time for repeat 2 of the toluene-acetate time series at a phase ratio of 4.

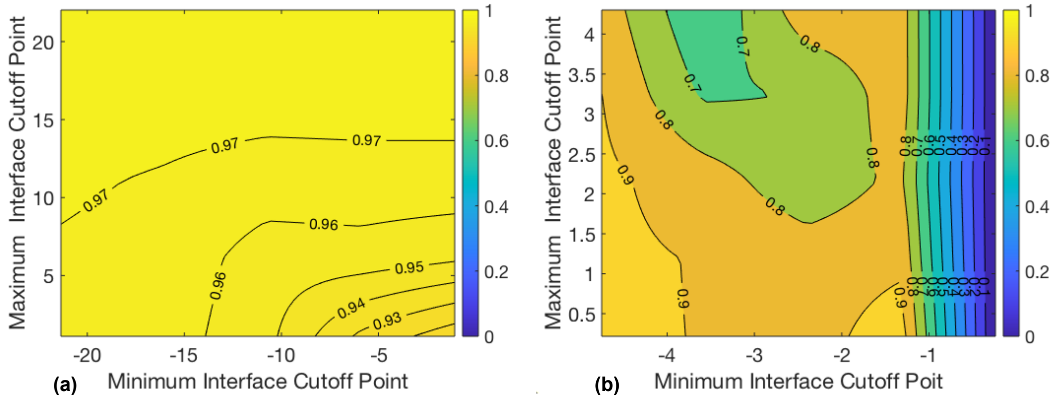

Figure S66: Contour plot of r^2 values for the sigmoidal curve fit for interface 1 data (a) and interface 2 data (b) depending on the maximum and minimum cut-off point combination – Toluene-acetate, phase ratio = 4, repeat 2.

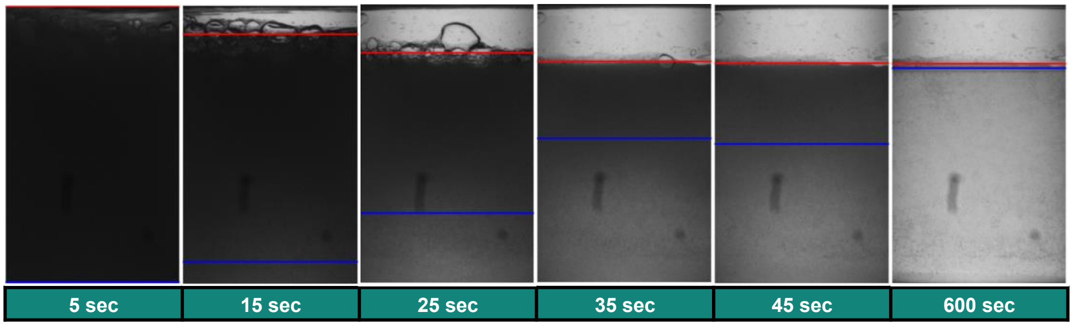

Figure S67: Sample images from toluene-acetate, phase ratio = 4, repeat 2 case with location of detected interfaces.

Toluene-acetate – Phase ratio = 4, Repeat 3

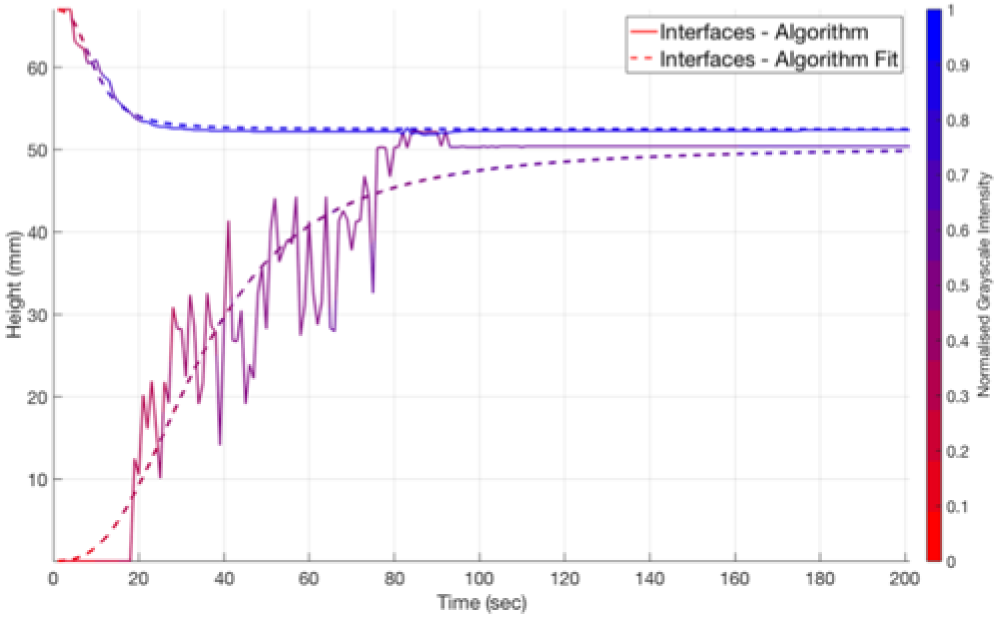

Figure S68: Detected interfaces and normalised grayscale intensity over time for repeat 3 of the toluene-acetate time series at a phase ratio of 4.

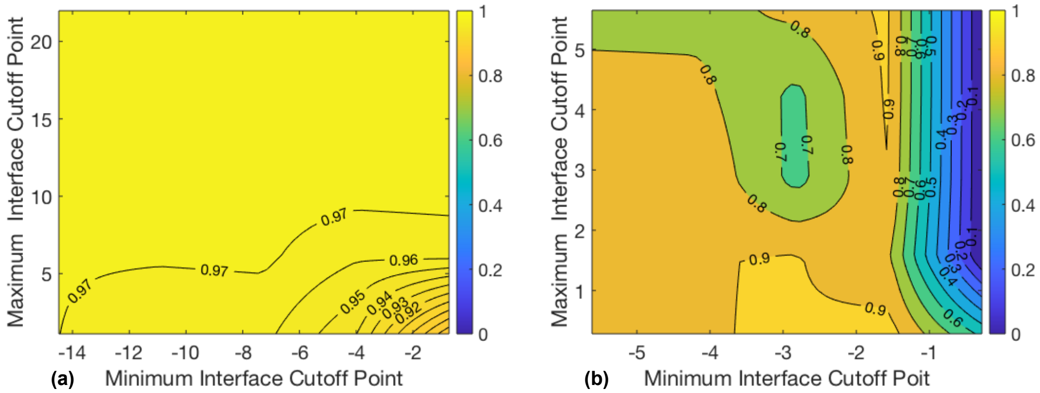

Figure S69: Contour plot of r^2 values for the sigmoidal curve fit for interface 1 data (a) and interface 2 data (b) depending on the maximum and minimum cut-off point combination – Toluene-acetate, phase ratio = 4, repeat 3.

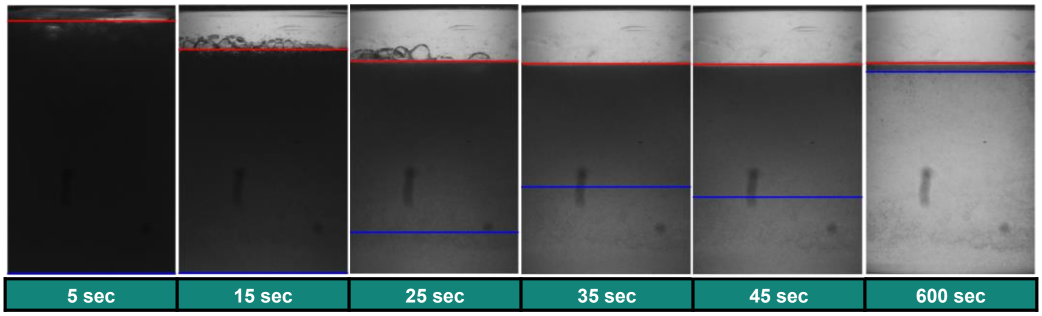

Figure S70: Sample images from toluene-acetate, phase ratio = 4, repeat 3 case with location of detected interfaces.

Toluene-acetate – Phase ratio = 4, Average

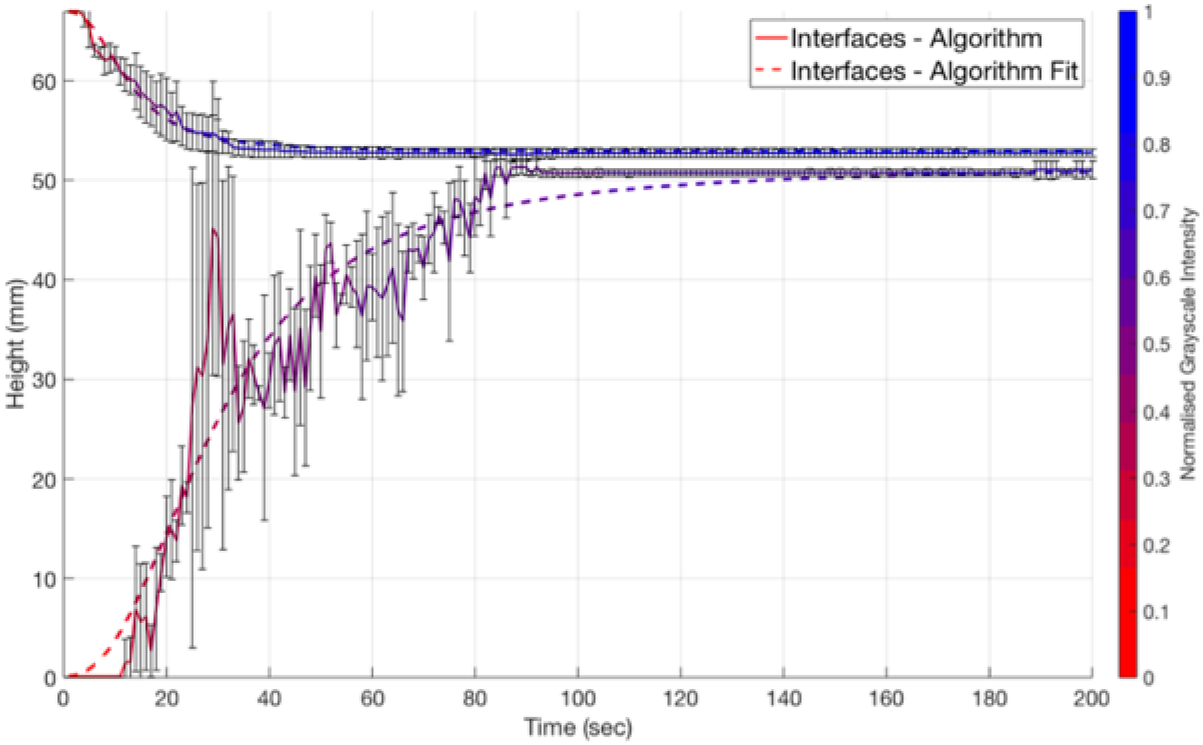

Figure S71: Averaged interface locations and normalised grayscale intensity over time for the toluene-acetate time series at a phase ratio of 4.

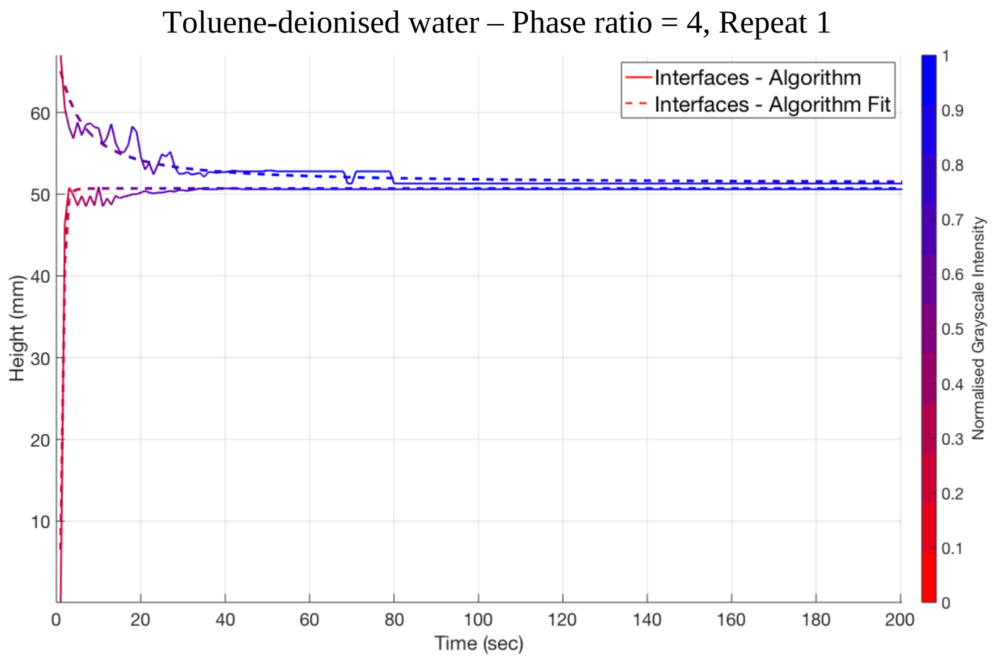

Figure S72: Detected interfaces and normalised grayscale intensity over time for repeat 1 of the toluene-deionised water time series at a phase ratio of 4.

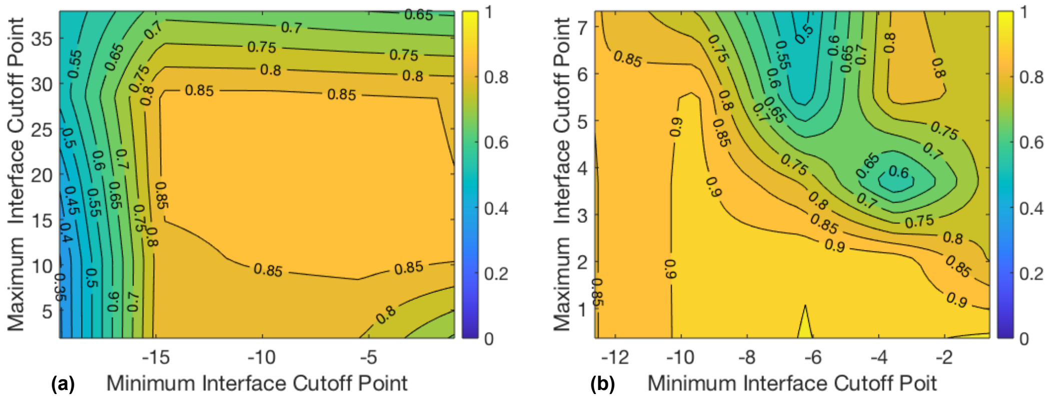

Figure S73: Contour plot of r^2 values for the sigmoidal curve fit for interface 1 data (a) and interface 2 data (b) depending on the maximum and minimum cut-off point combination – Toluene-deionised water, phase ratio = 4, repeat 1.

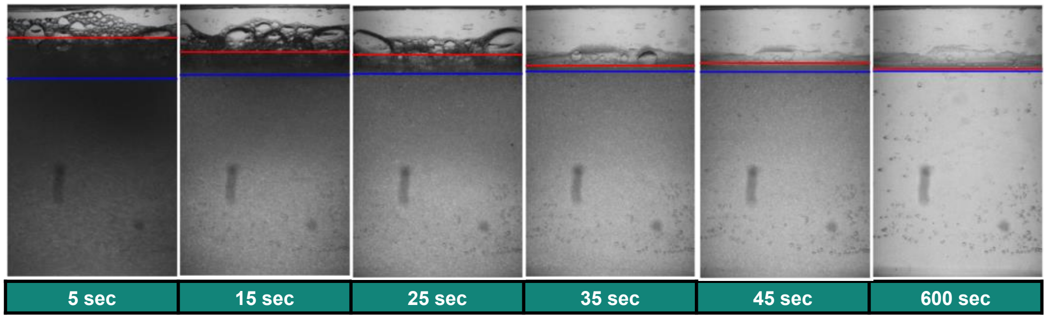

Figure S74: Sample images from toluene-deionised water, phase ratio = 4, repeat 1 case with location of detected interfaces.

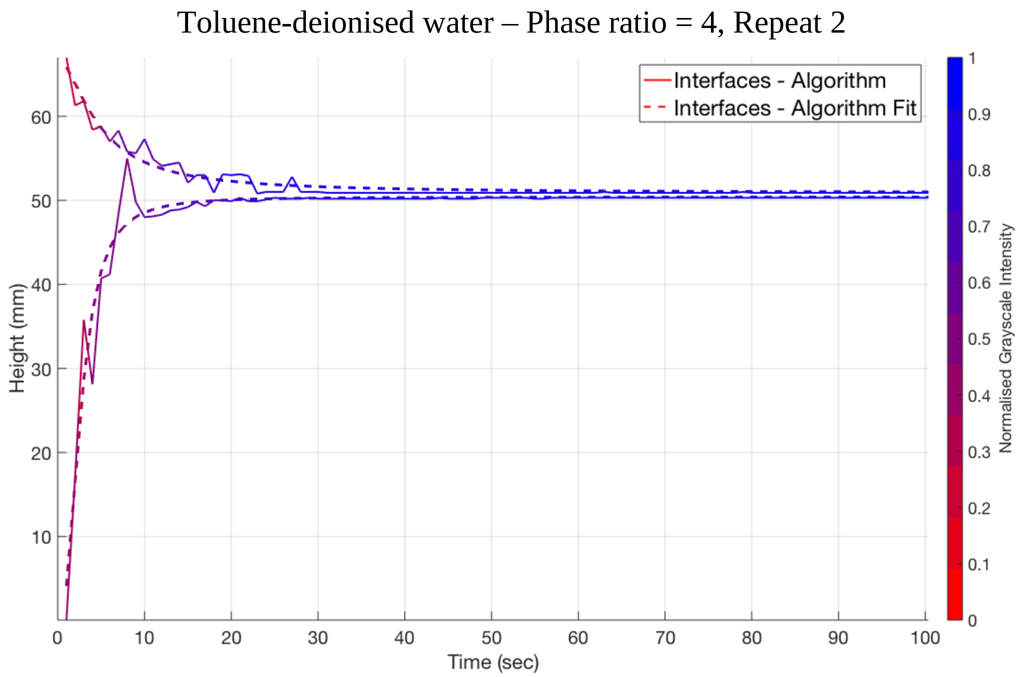

Figure S75: Detected interfaces and normalised grayscale intensity over time for repeat 2 of the toluene-deionised water time series at a phase ratio of 4.

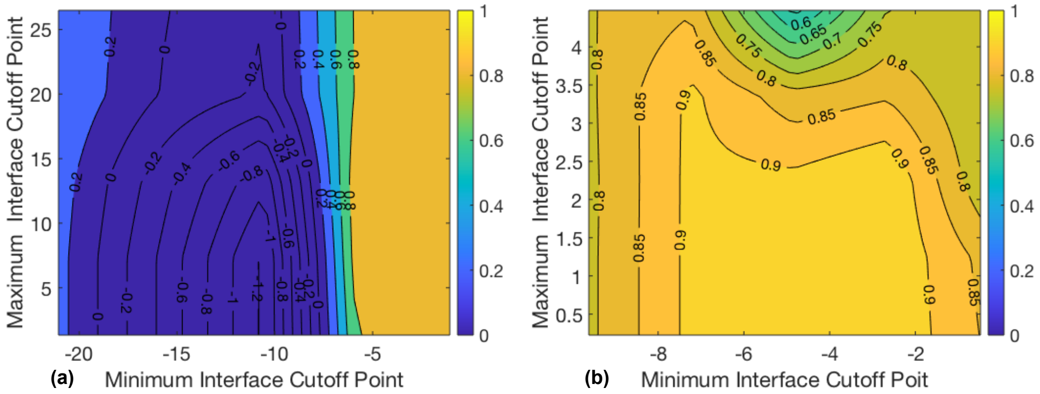

Figure S76: Contour plot of r^2 values for the sigmoidal curve fit for interface 1 data (a) and interface 2 data (b) depending on the maximum and minimum cut-off point combination – Toluene-deionised water, phase ratio = 4, repeat 2.

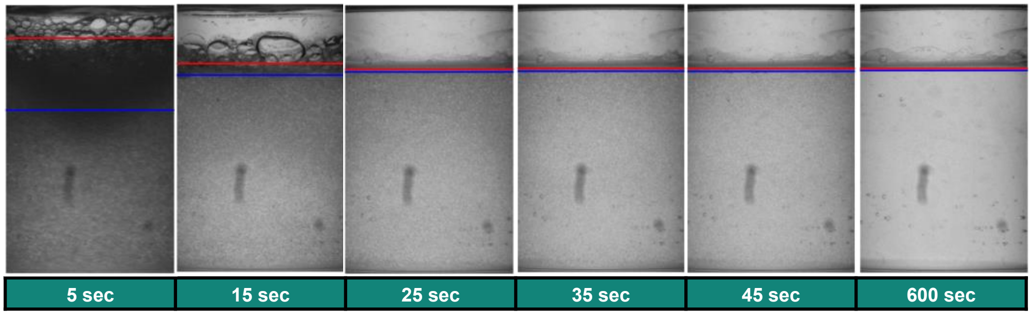

Figure S77: Sample images from toluene-deionised water, phase ratio = 4, repeat 2 case with location of detected interfaces.

Toluene-deionised water – Phase ratio = 4, Repeat 3

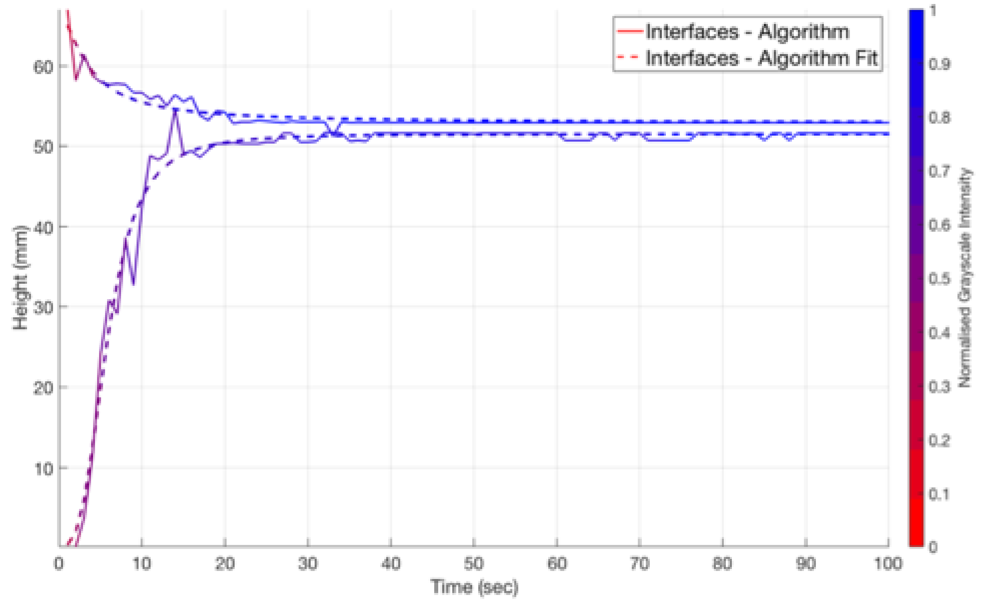

Figure S78: Detected interfaces and normalised grayscale intensity over time for repeat 3 of the toluene-deionised water time series at a phase ratio of 4.

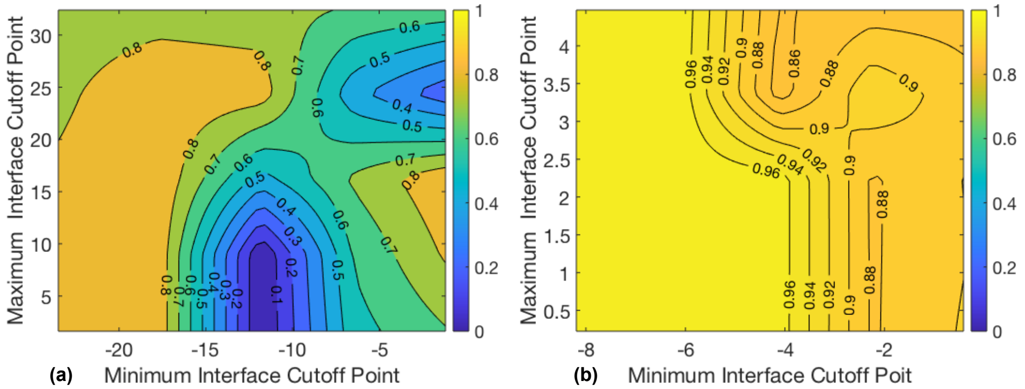

Figure S79: Contour plot of r^2 values for the sigmoidal curve fit for interface 1 data (a) and interface 2 data (b) depending on the maximum and minimum cut-off point combination – Toluene-deionised water, phase ratio = 4, repeat 3.

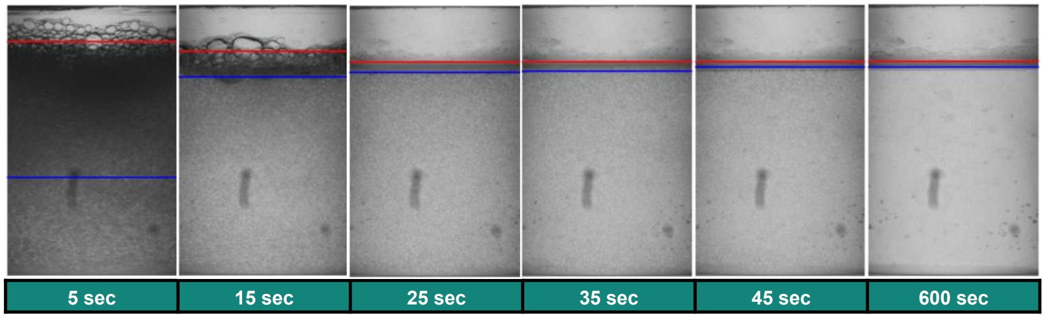

Figure S80: Sample images from toluene-deionised water, phase ratio = 4, repeat 3 case with location of detected interfaces.

Toluene-deionised water – Phase ratio = 4, Average

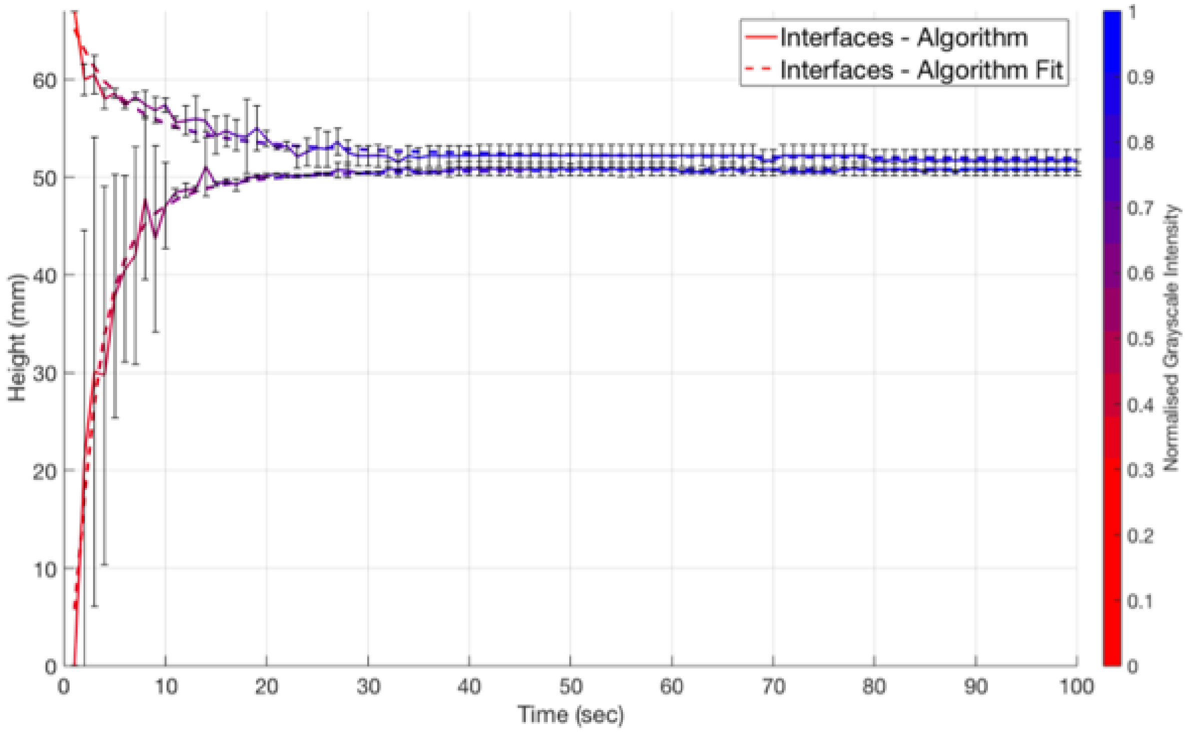

Figure S81: Averaged interface locations and normalised grayscale intensity over time for the toluene-deionised water time series at a phase ratio of 4.

Toluene-glycine – Phase ratio = 4, Repeat 1

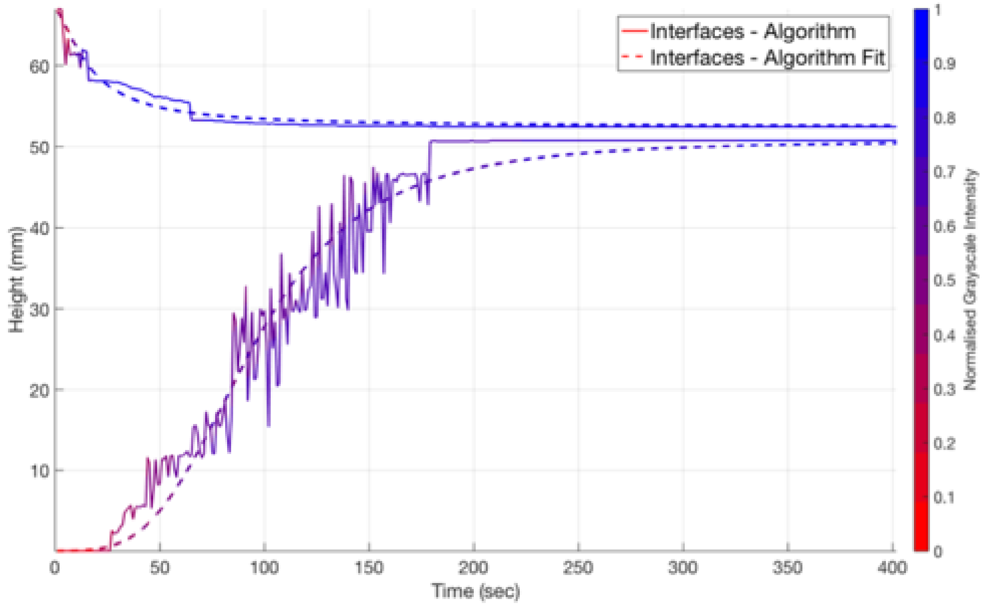

Figure S82: Detected interfaces and normalised grayscale intensity over time for repeat 1 of the toluene-glycine time series at a phase ratio of 4.

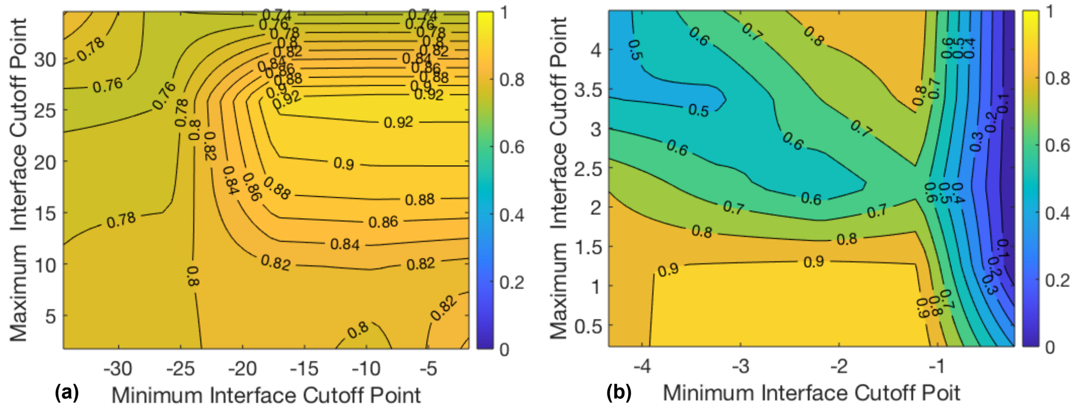

Figure S83: Contour plot of r^2 values for the sigmoidal curve fit for interface 1 data (a) and interface 2 data (b) depending on the maximum and minimum cut-off point combination – Toluene-glycine, phase ratio = 4, repeat 1.

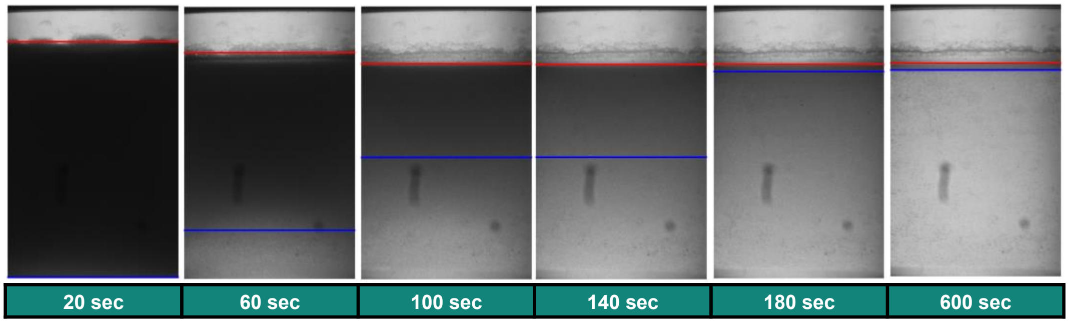

Figure S84: Sample images from toluene-glycine, phase ratio = 4, repeat 1 case with location of detected interfaces.

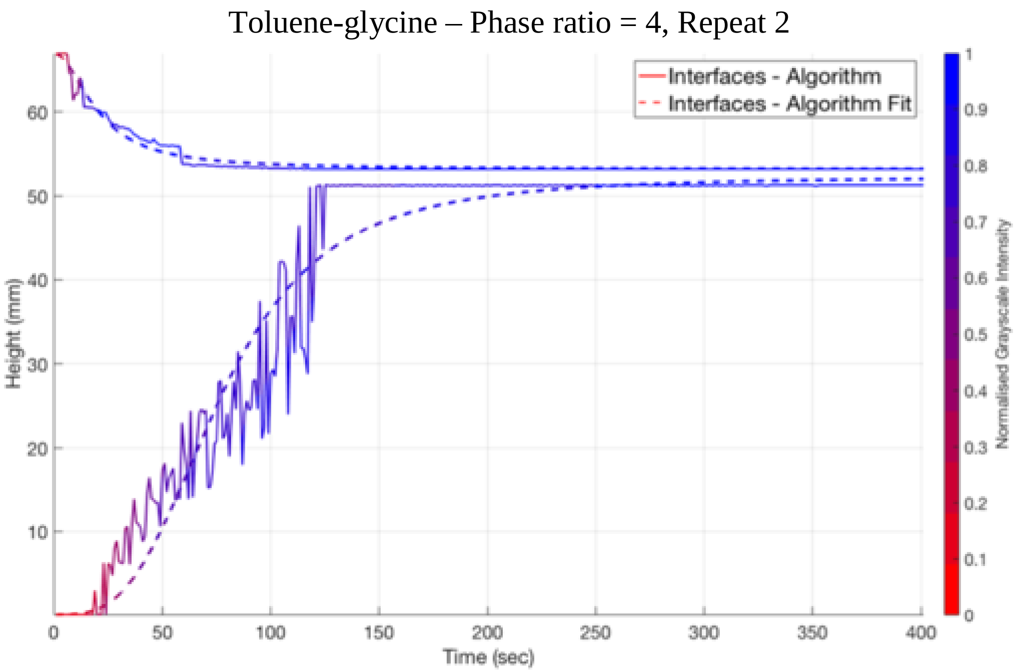

Figure S85: Detected interfaces and normalised grayscale intensity over time for repeat 2 of the toluene-glycine time series at a phase ratio of 4.

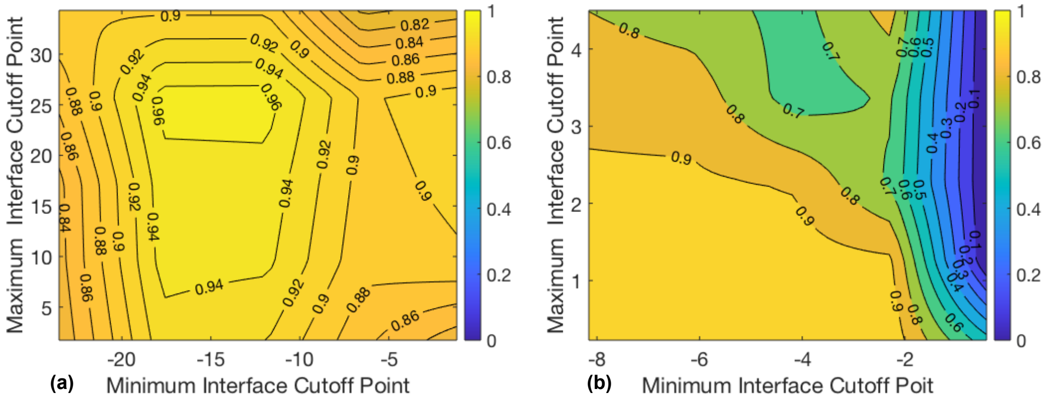

Figure S86: Contour plot of r^2 values for the sigmoidal curve fit for interface 1 data (a) and interface 2 data (b) depending on the maximum and minimum cut-off point combination – Toluene-glycine, phase ratio = 4, repeat 2.

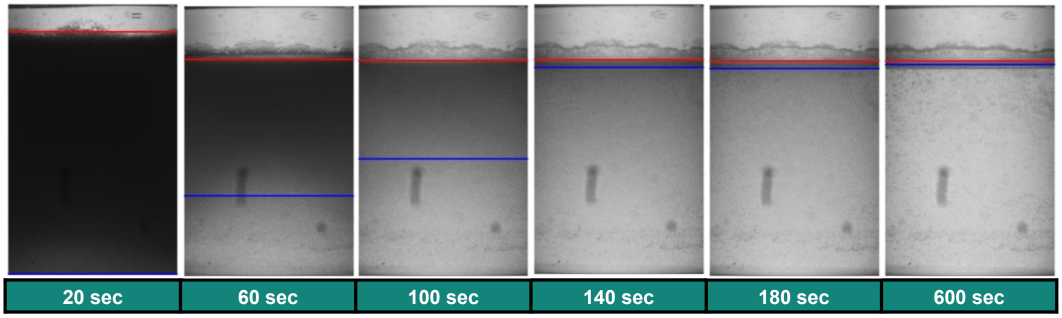

Figure S87: Sample images from toluene-glycine, phase ratio = 4, repeat 2 case with location of detected interfaces.

Toluene-glycine – Phase ratio = 4, Repeat 3

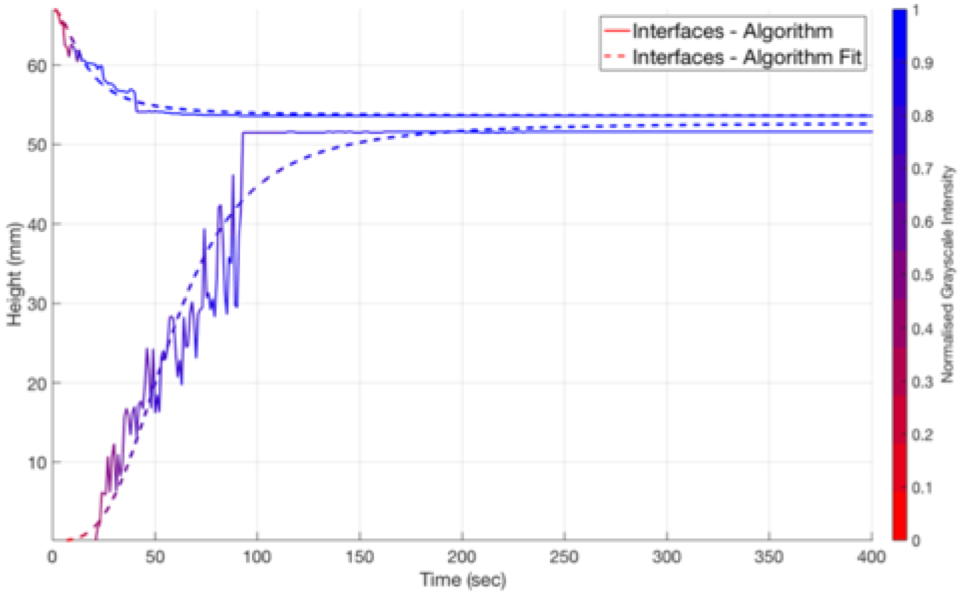

Figure S88: Detected interfaces and normalised grayscale intensity over time for repeat 3 of the toluene-glycine time series at a phase ratio of 4.

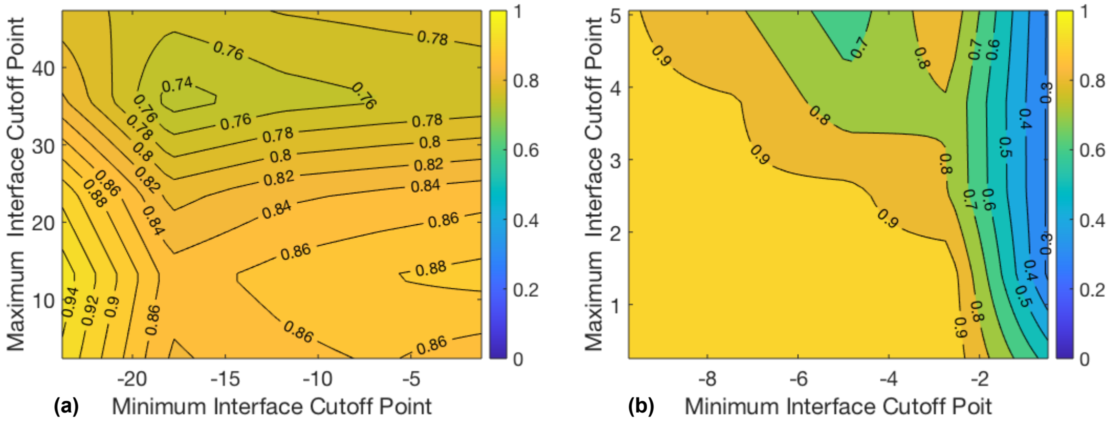

Figure S89: Contour plot of r^2 values for the sigmoidal curve fit for interface 1 data (a) and interface 2 data (b) depending on the maximum and minimum cut-off point combination – Toluene-glycine, phase ratio = 4, repeat 3.

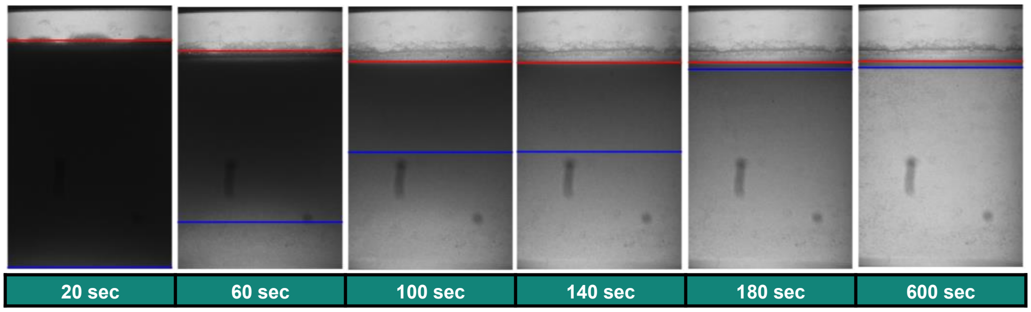

Figure S90: Sample images from toluene-glycine, phase ratio = 4, repeat 3 case with location of detected interfaces.

Toluene-glycine – Phase ratio = 4, Average

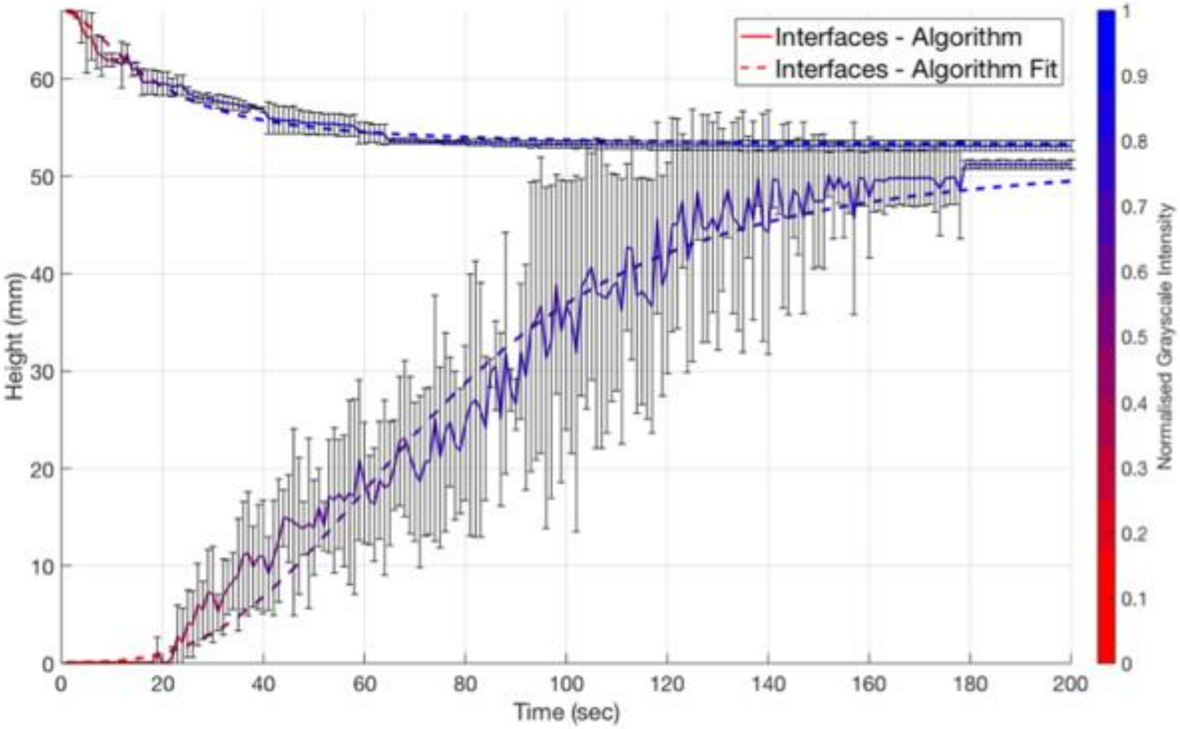

Figure S91: Averaged interface locations and normalised grayscale intensity over time for the toluene-glycine time series at a phase ratio of 4.

4.2 Experiment 2

Vial 1 - Toluene-water, 0.01M SDBS, 0.0453g/100ml NaCl

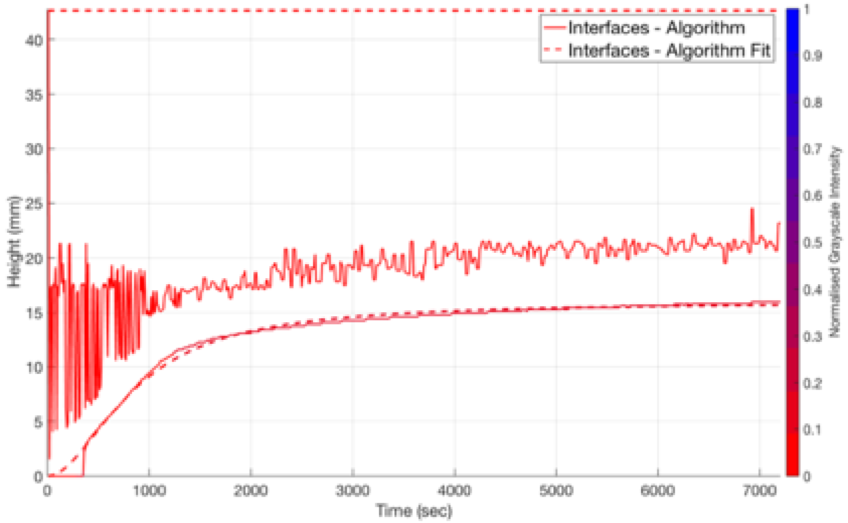

Figure S92: Detected interfaces and normalised grayscale intensity over time of the 0.01M SDBS solution (0.453g/100ml NaCl).

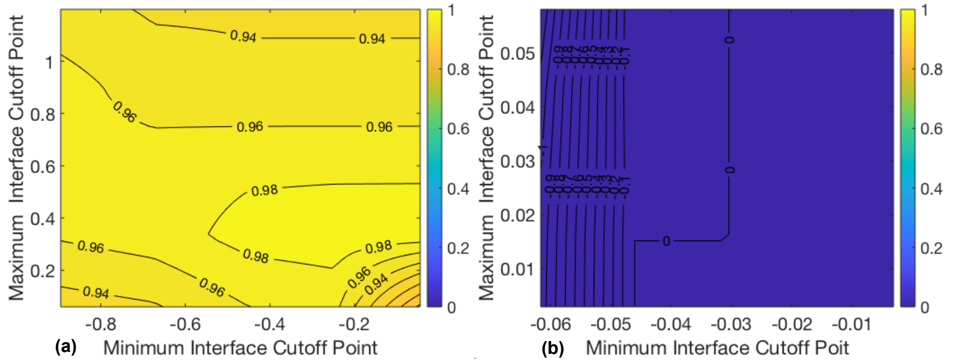

Figure S93: Contour plot of r^2 values for the sigmoidal curve fit for interface 1 data (a) and interface 2 data (b) depending on the maximum and minimum cut-off point combination – 0.01M SDBS solution (0.453g/100ml NaCl).

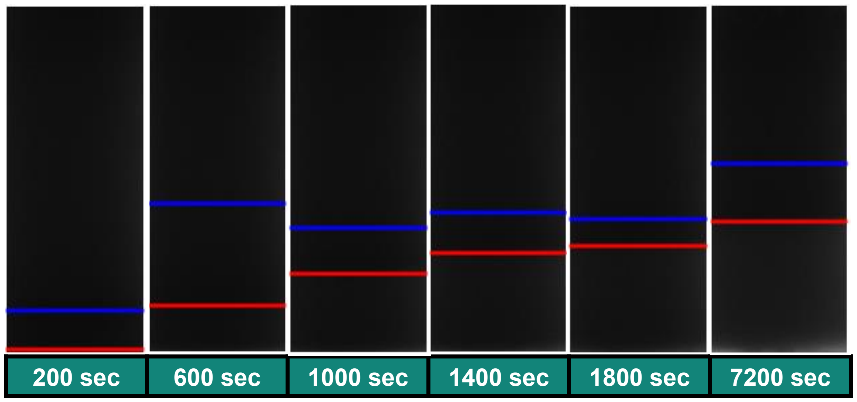

Figure S94: Sample images from 0.01M SDBS solution (0.453g/100ml NaCl) with location of detected interfaces.

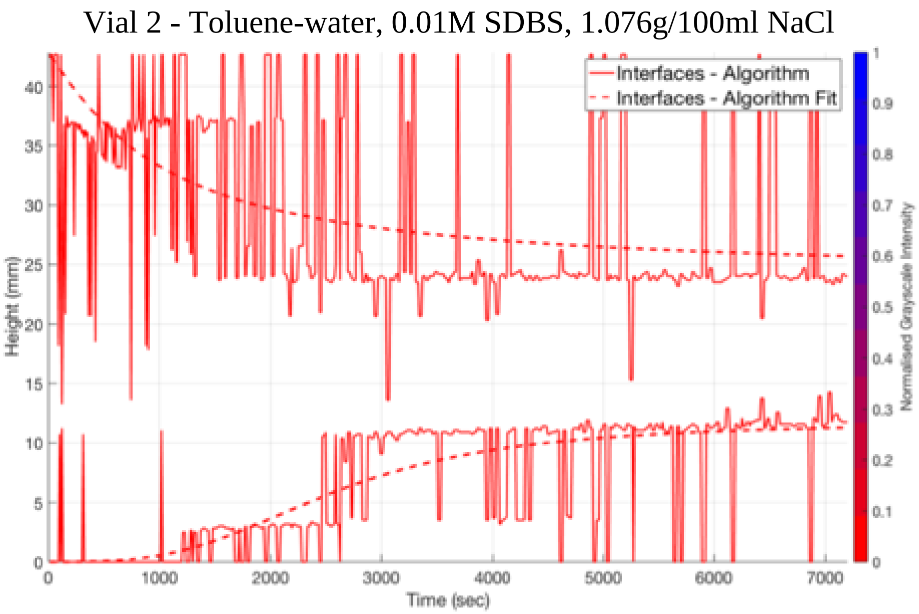

Figure S95: Detected interfaces and normalised grayscale intensity over time of the 0.01M SDBS solution (1.076g/100ml NaCl).

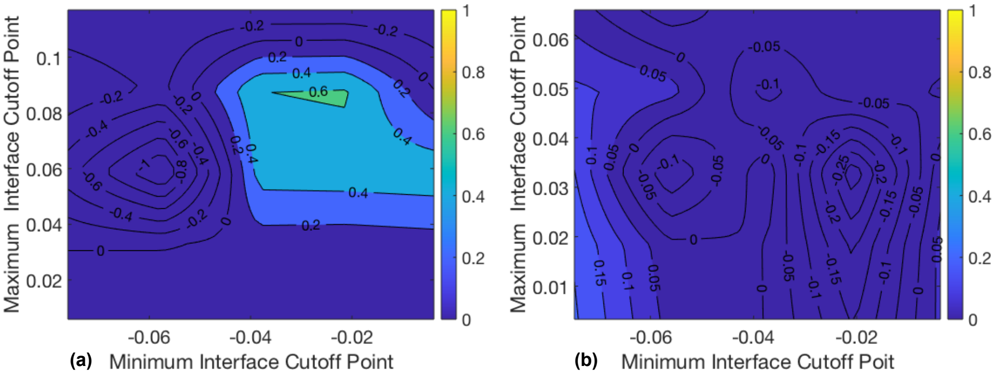

Figure S96: Contour plot of r^2 values for the sigmoidal curve fit for interface 1 data (a) and interface 2 data (b) depending on the maximum and minimum cut-off point combination – 0.01M SDBS solution (1.076g/100ml NaCl).

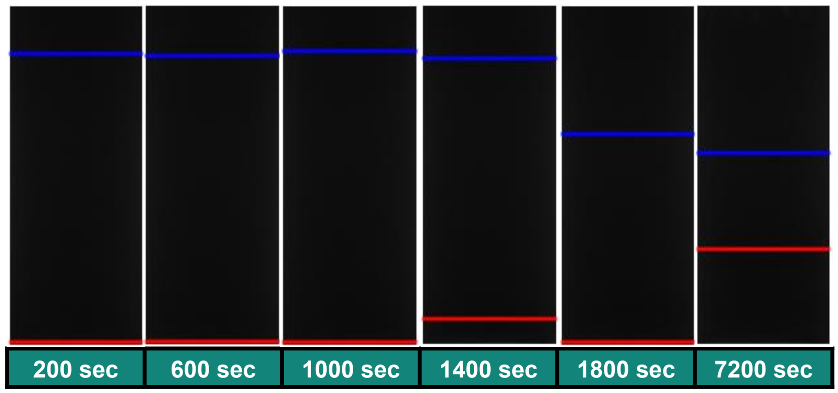

Figure S97: Sample images from 0.01M SDBS solution (1.076g/100ml NaCl) with location of detected interfaces.

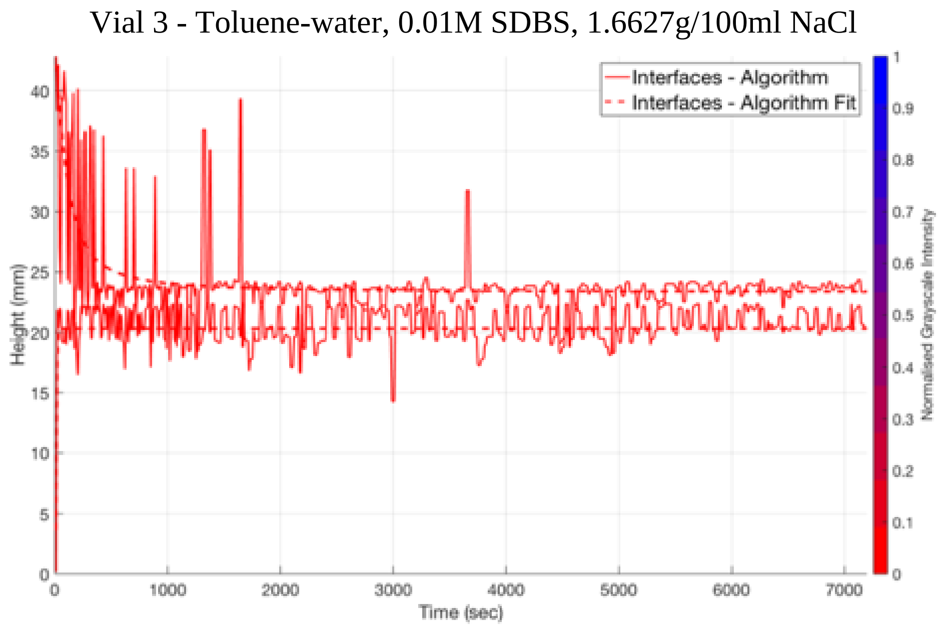

Figure S98: Detected interfaces and normalised grayscale intensity over time of the 0.01M SDBS solution (1.6627g/100ml NaCl).

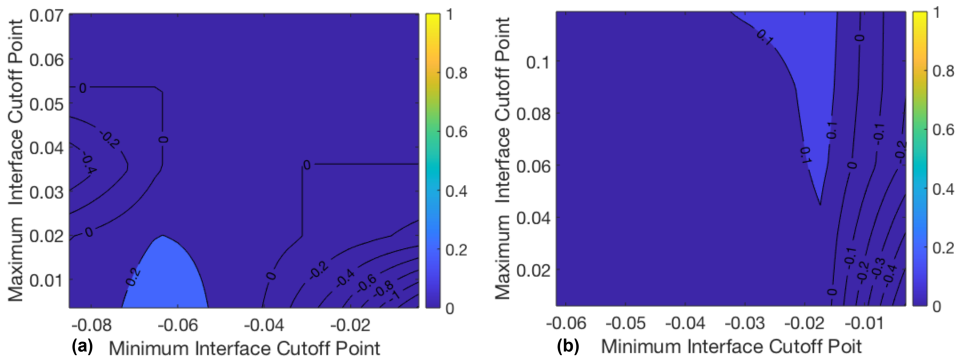

Figure S99: Contour plot of r^2 values for the sigmoidal curve fit for interface 1 data (a) and interface 2 data (b) depending on the maximum and minimum cut-off point combination – 0.01M SDBS solution (1.6627g/100ml NaCl).

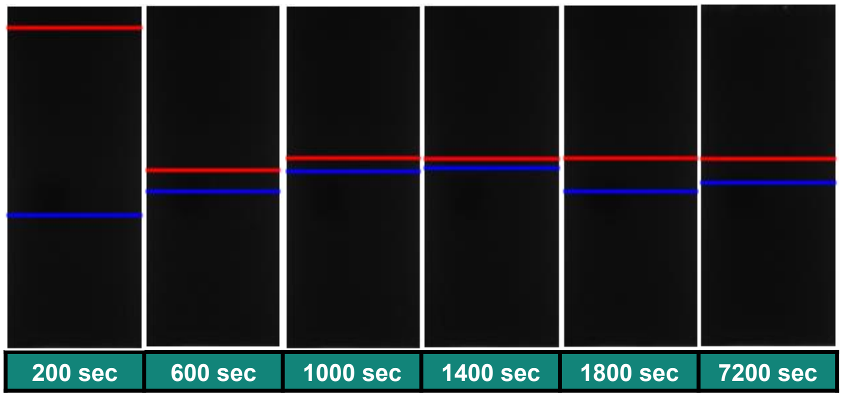

Figure S100: Sample images from 0.01M SDBS solution (1.6627g/100ml NaCl) with location of detected interfaces.

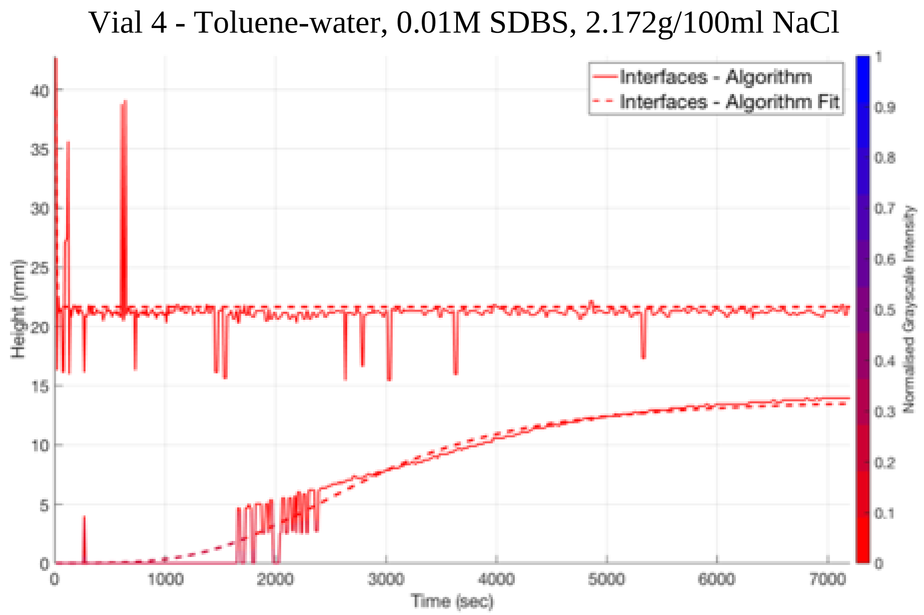

Figure S101: Detected interfaces and normalised grayscale intensity over time of the 0.01M SDBS solution (2.172g/100ml NaCl).

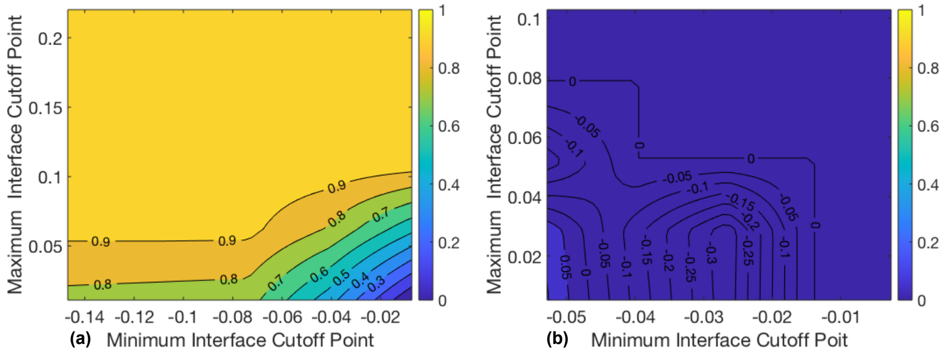

Figure S102: Contour plot of r^2 values for the sigmoidal curve fit for interface 1 data (a) and interface 2 data (b) depending on the maximum and minimum cut-off point combination – 0.01M SDBS solution (2.172g/100ml NaCl).

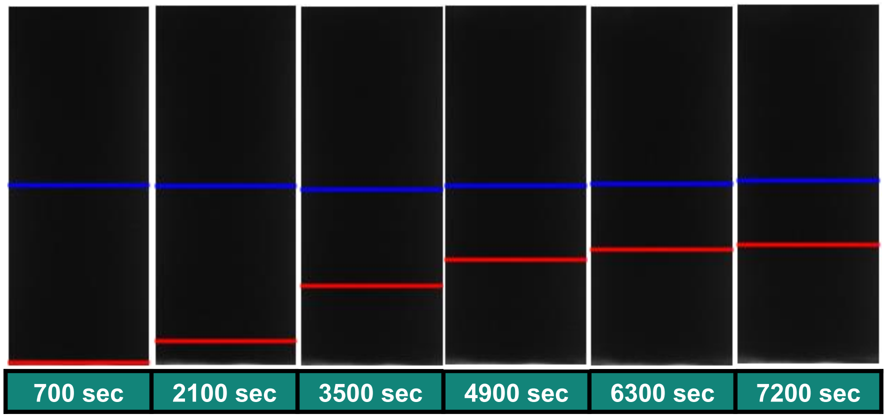

Figure S103: Sample images from 0.01M SDBS solution (2.172g/100ml NaCl) with location of detected interfaces.

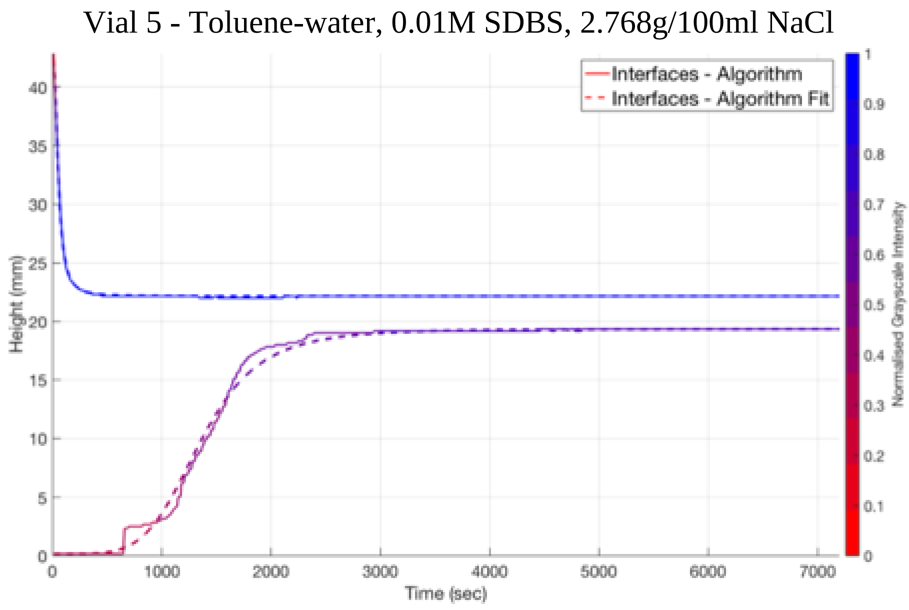

Figure S104: Detected interfaces and normalised grayscale intensity over time of the 0.01M SDBS solution (2.768g/100ml NaCl).

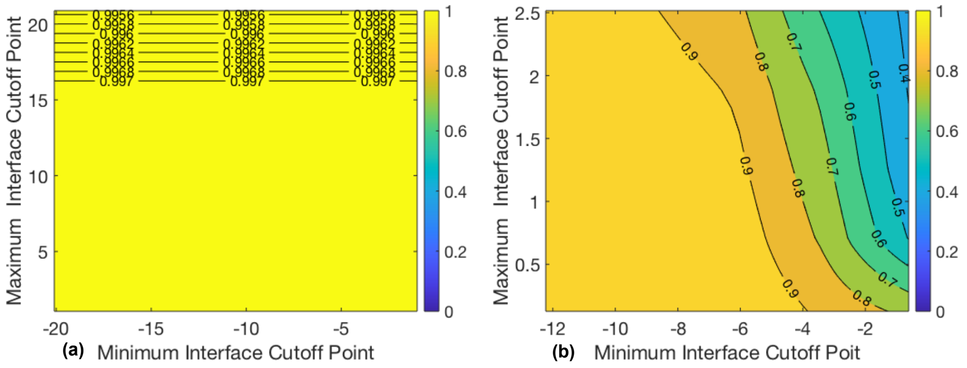

Figure S105: Contour plot of r^2 values for the sigmoidal curve fit for interface 1 data (a) and interface 2 data (b) depending on the maximum and minimum cut-off point combination – 0.01M SDBS solution (2.768g/100ml NaCl).

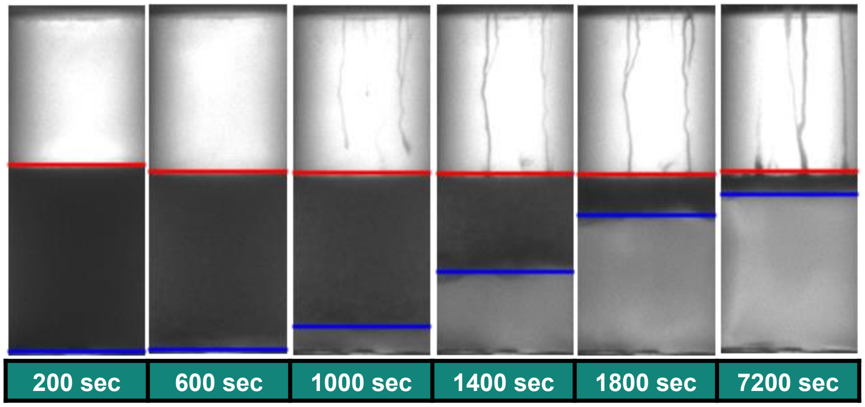

Figure S106: Sample images from 0.01M SDBS solution (2.768g/100ml NaCl) with location of detected interfaces.

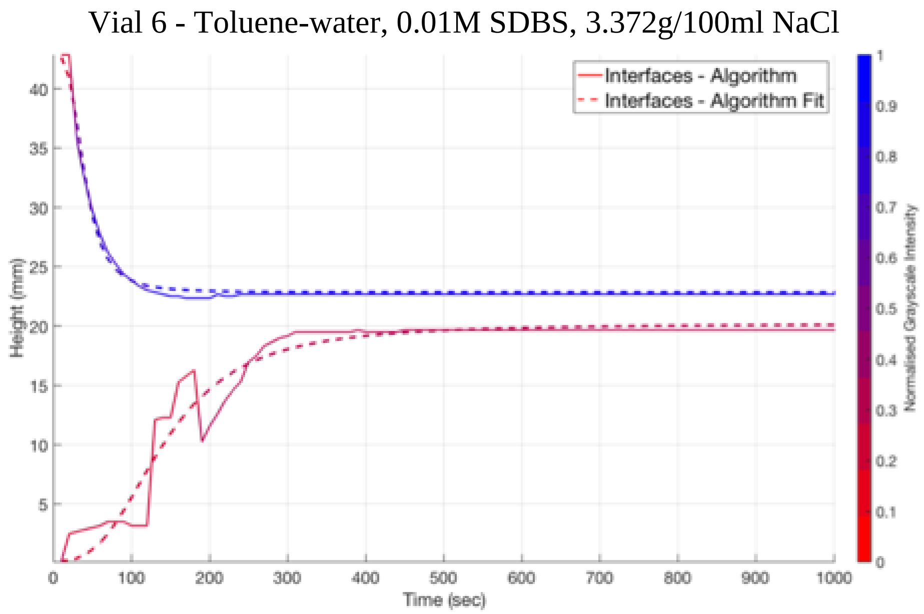

Figure S107: Detected interfaces and normalised grayscale intensity over time of the 0.01M SDBS solution (3.372g/100ml NaCl).

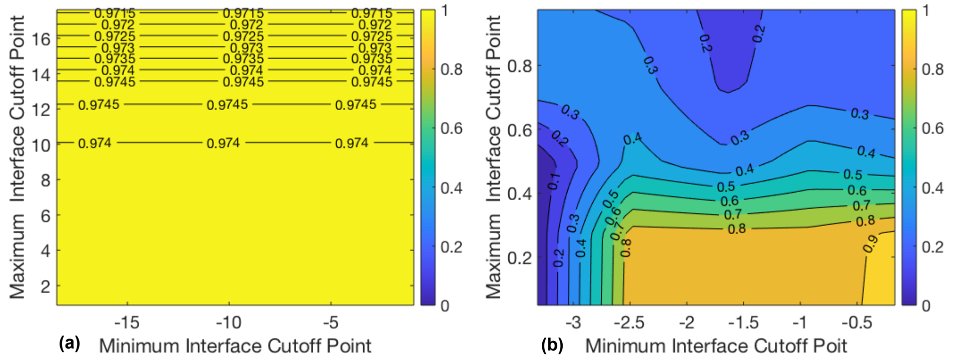

Figure S108: Contour plot of r^2 values for the sigmoidal curve fit for interface 1 data (a) and interface 2 data (b) depending on the maximum and minimum cut-off point combination – 0.01M SDBS solution (3.372g/100ml NaCl).

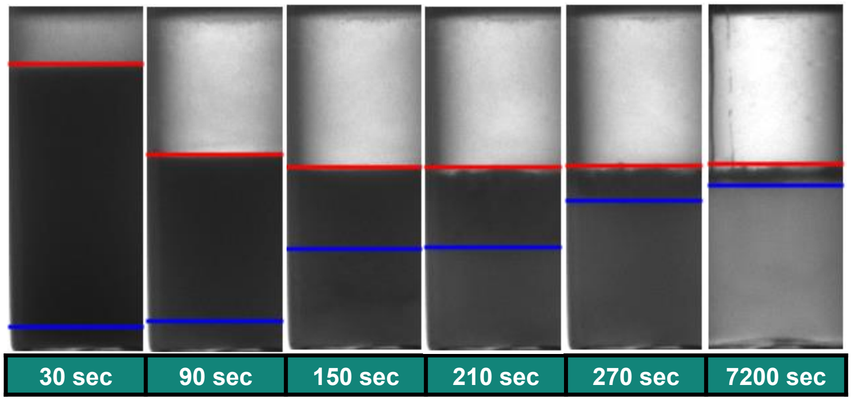

Figure S109: Sample images from 0.01M SDBS solution (3.372g/100ml NaCl) with location of detected interfaces.

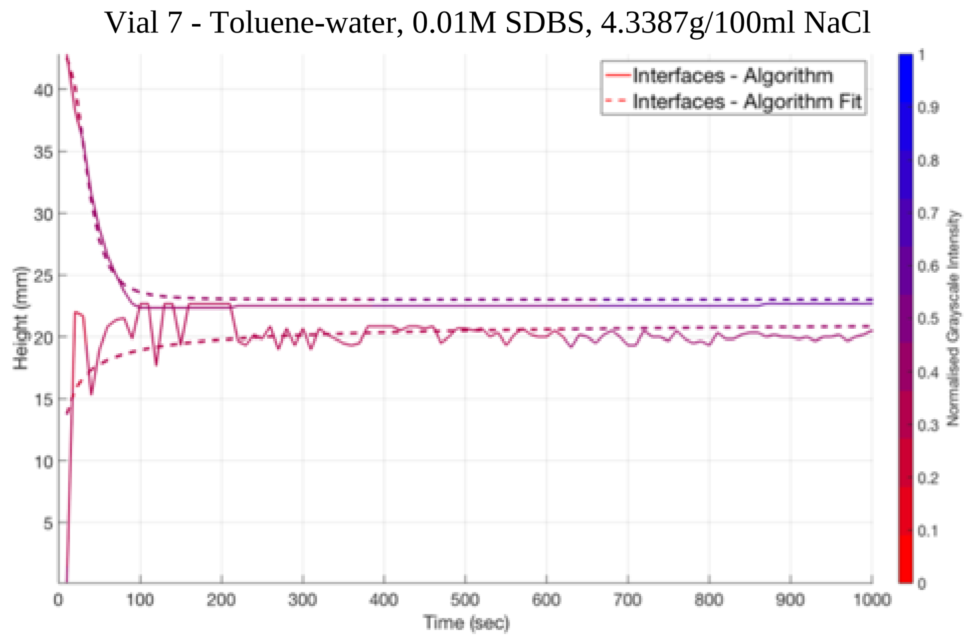

Figure S110: Detected interfaces and normalised grayscale intensity over time of the 0.01M SDBS solution (4.3387g/100ml NaCl).

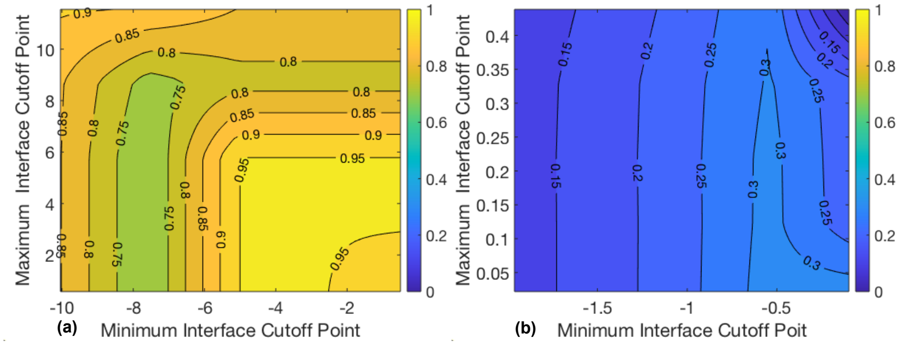

Figure S111: Contour plot of r^2 values for the sigmoidal curve fit for interface 1 data (a) and interface 2 data (b) depending on the maximum and minimum cut-off point combination – 0.01M SDBS solution (4.3387g/100ml NaCl).

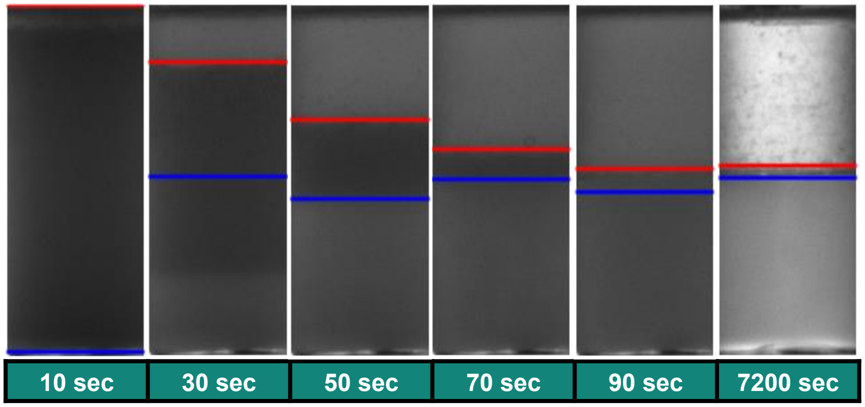

Figure S112: Sample images from 0.01M SDBS solution (4.3387g/100ml NaCl) with location of detected interfaces.

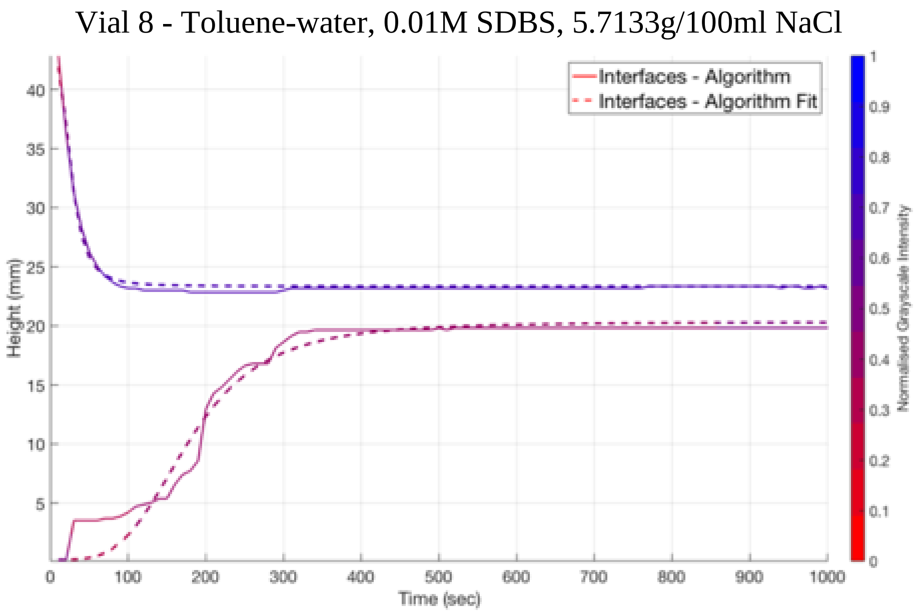

Figure S113: Detected interfaces and normalised grayscale intensity over time of the 0.01M SDBS solution (5.7133g/100ml NaCl).

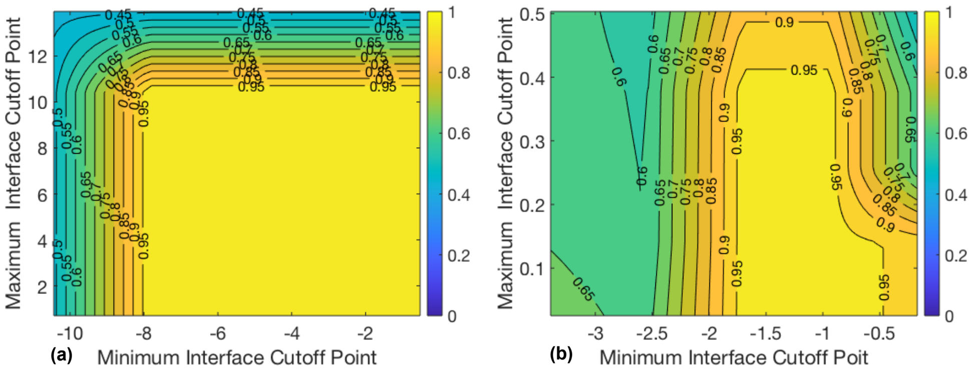

Figure S114: Contour plot of r^2 values for the sigmoidal curve fit for interface 1 data (a) and interface 2 data (b) depending on the maximum and minimum cut-off point combination – 0.01M SDBS solution (5.7133g/100ml NaCl).

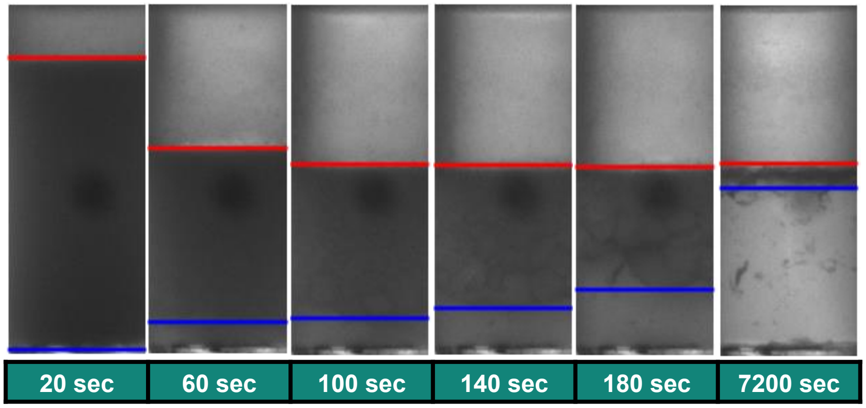

Figure S115: Sample images from 0.01M SDBS solution (5.7133g/100ml NaCl) with location of detected interfaces.

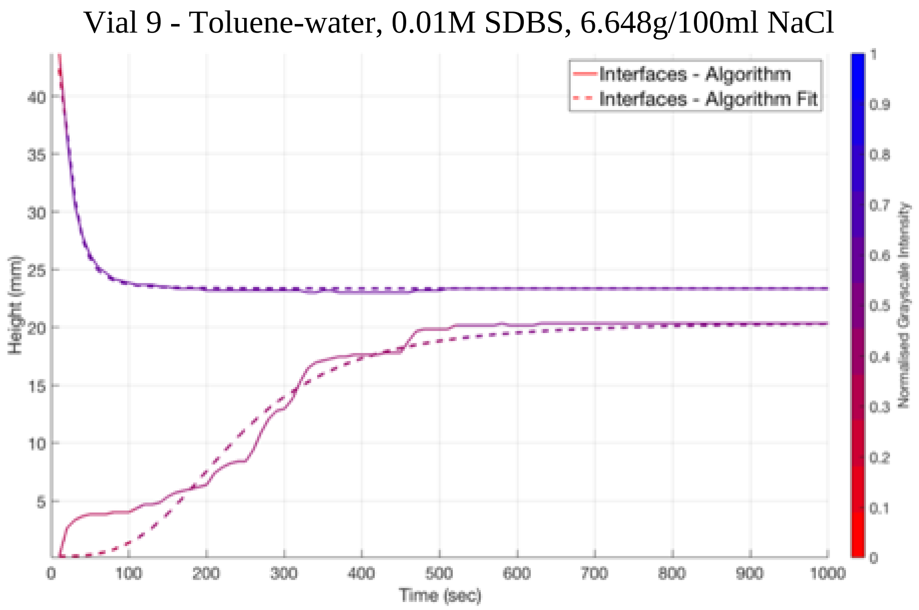

Figure S116: Detected interfaces and normalised grayscale intensity over time of the 0.01M SDBS solution (6.648g/100ml NaCl).

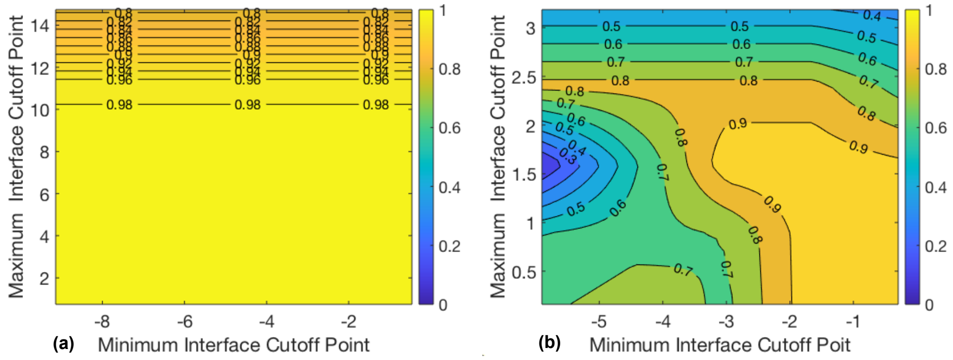

Figure S117: Contour plot of r^2 values for the sigmoidal curve fit for interface 1 data (a) and interface 2 data (b) depending on the maximum and minimum cut-off point combination – 0.01M SDBS solution (6.648g/100ml NaCl).

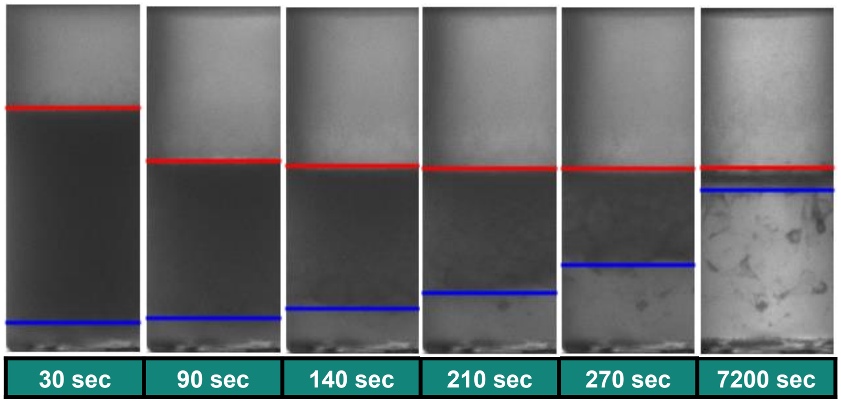

Figure S118: Sample images from 0.01M SDBS solution (6.648g/100ml NaCl) with location of detected interfaces.

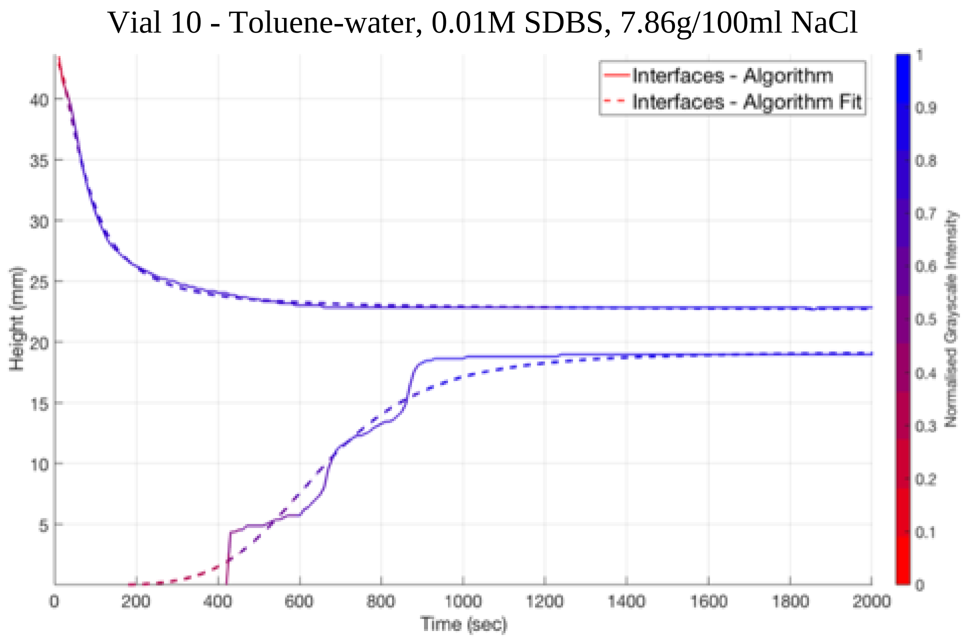

Figure S119: Detected interfaces and normalised grayscale intensity over time of the 0.01M SDBS solution (7.86g/100ml NaCl).

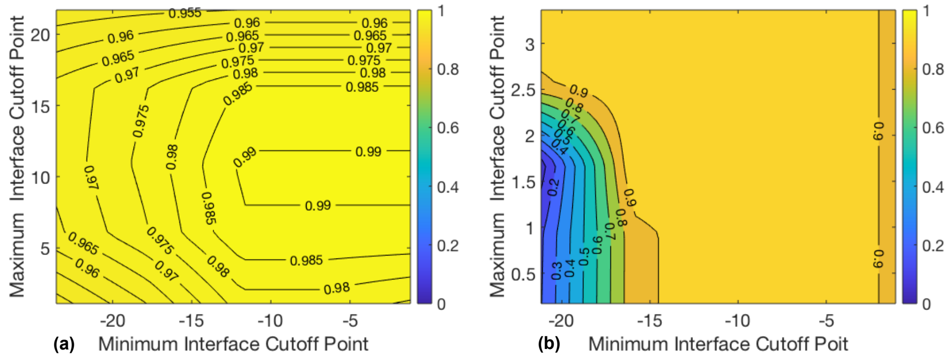

Figure S120: Contour plot of r^2 values for the sigmoidal curve fit for interface 1 data (a) and interface 2 data (b) depending on the maximum and minimum cut-off point combination – 0.01M SDBS solution (7.86g/100ml NaCl).

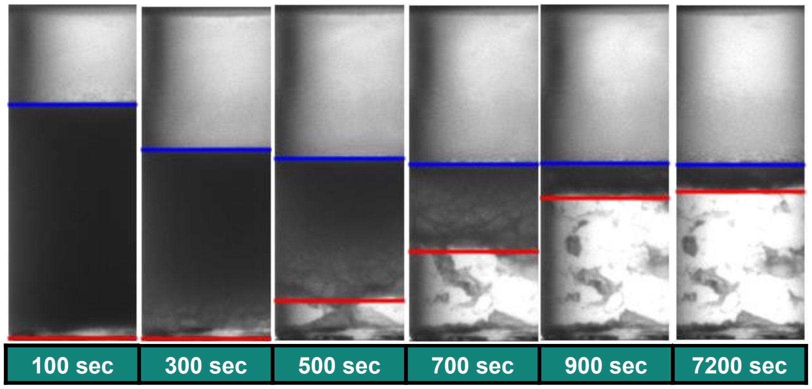

Figure S121: Sample images from 0.01M SDBS solution (7.86g/100ml NaCl) with location of detected interfaces.

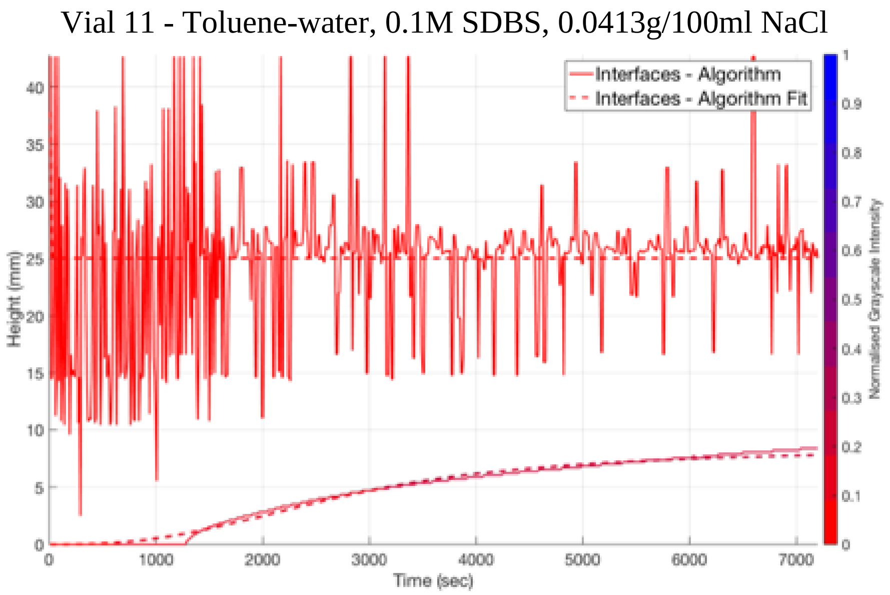

Figure S122: Detected interfaces and normalised grayscale intensity over time of the 0.1M SDBS solution (0.0413g/100ml NaCl).

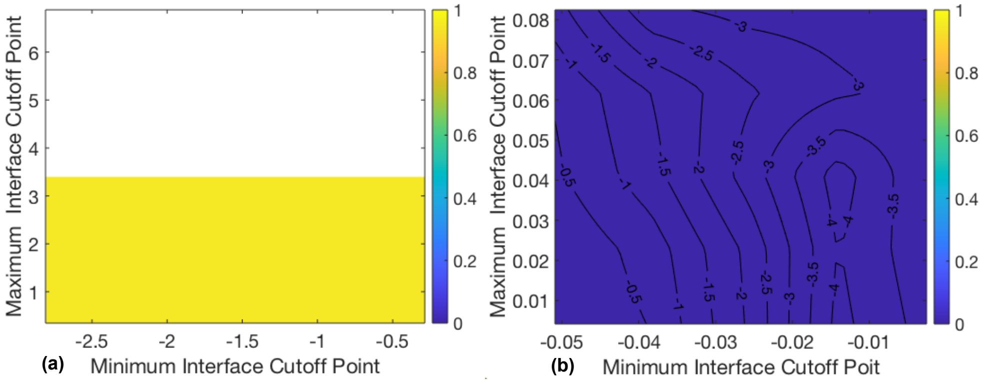

Figure S123: Contour plot of r^2 values for the sigmoidal curve fit for interface 1 data (a) and interface 2 data (b) depending on the maximum and minimum cut-off point combination – 0.1M SDBS solution (0.0413g/100ml NaCl).

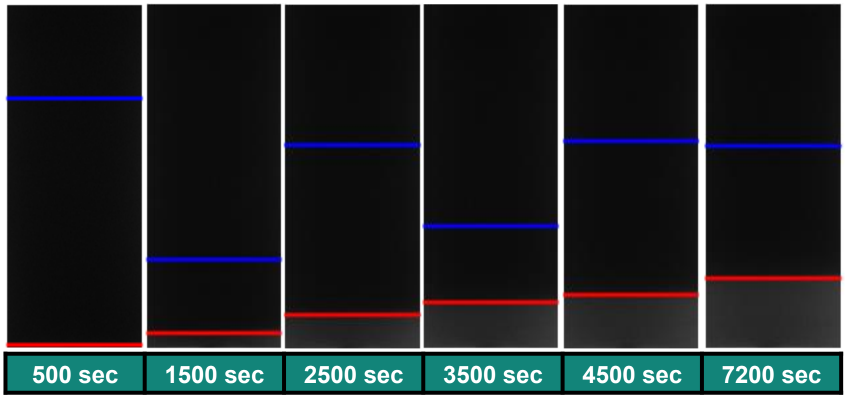

Figure S124: Sample images from 0.1M SDBS solution (0.0413g/100ml NaCl) with location of detected interfaces.

Vial 12 - Toluene-water, 0.1M SDBS, 1.076g/100ml NaCl

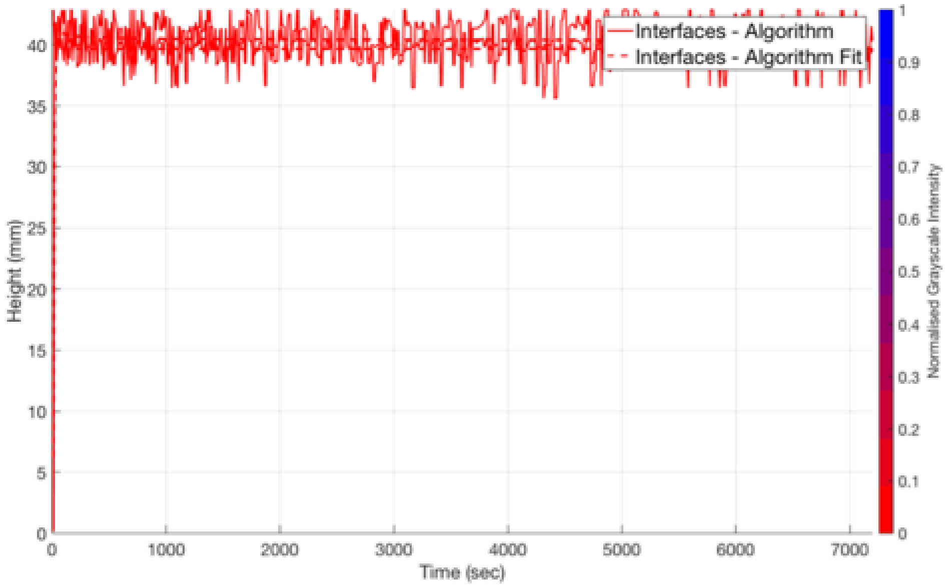

Figure S125: Detected interfaces and normalised grayscale intensity over time of the 0.1M SDBS solution (1.076g/100ml NaCl).

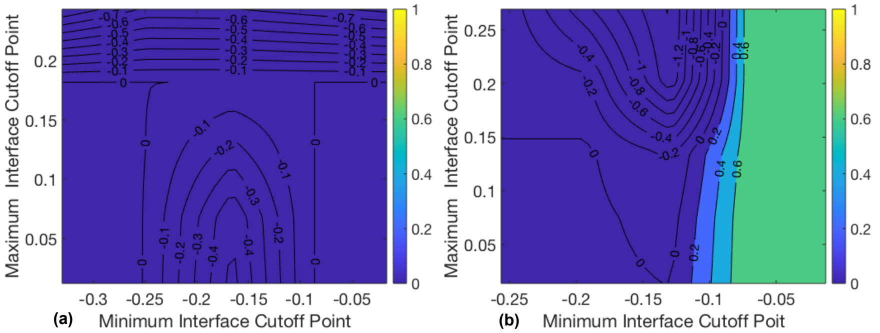

Figure S126: Contour plot of r^2 values for the sigmoidal curve fit for interface 1 data (a) and interface 2 data (b) depending on the maximum and minimum cut-off point combination – 0.1M SDBS solution (1.076g/100ml NaCl).

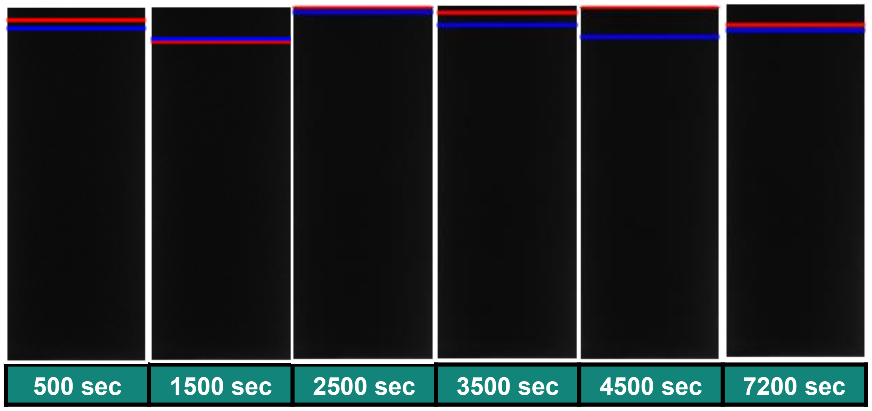

Figure S127: Sample images from 0.1M SDBS solution (1.076g/100ml NaCl) with location of detected interfaces.

Vial 13 - Toluene-water, 0.1M SDBS, 1.5907g/100ml NaCl

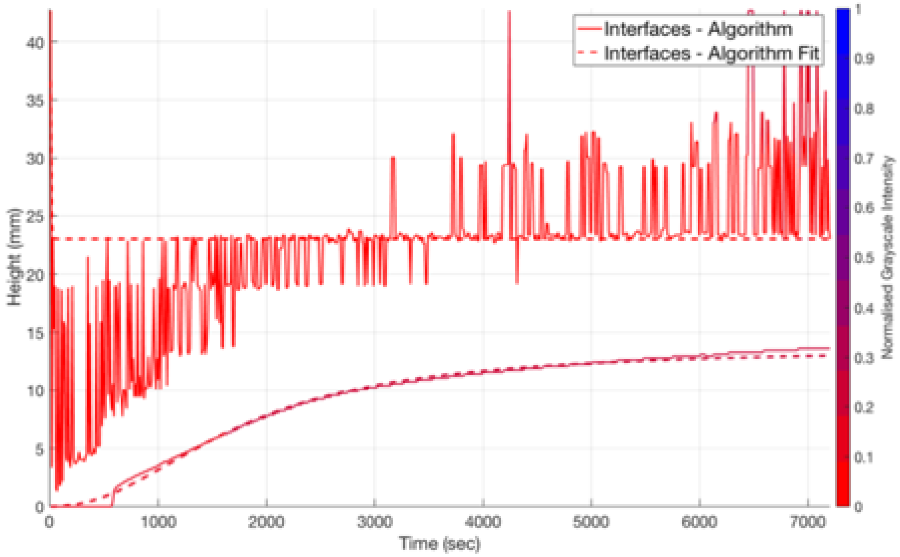

Figure S128: Detected interfaces and normalised grayscale intensity over time of the 0.1M SDBS solution (1.5907g/100ml NaCl).

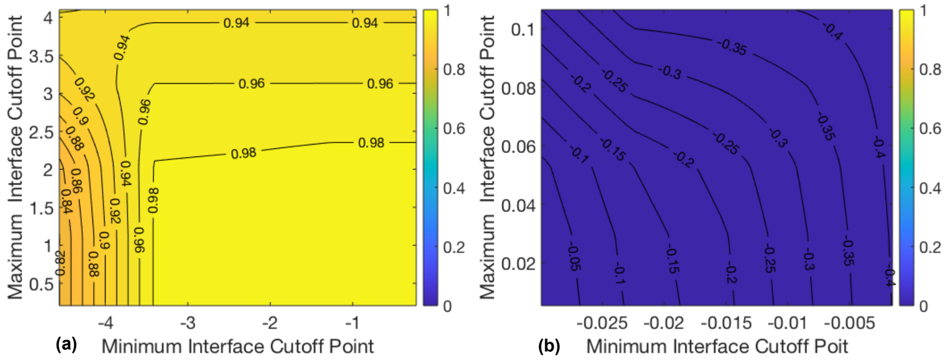

Figure S129: Contour plot of r^2 values for the sigmoidal curve fit for interface 1 data (a) and interface 2 data (b) depending on the maximum and minimum cut-off point combination – 0.1M SDBS solution (1.5907g/100ml NaCl).

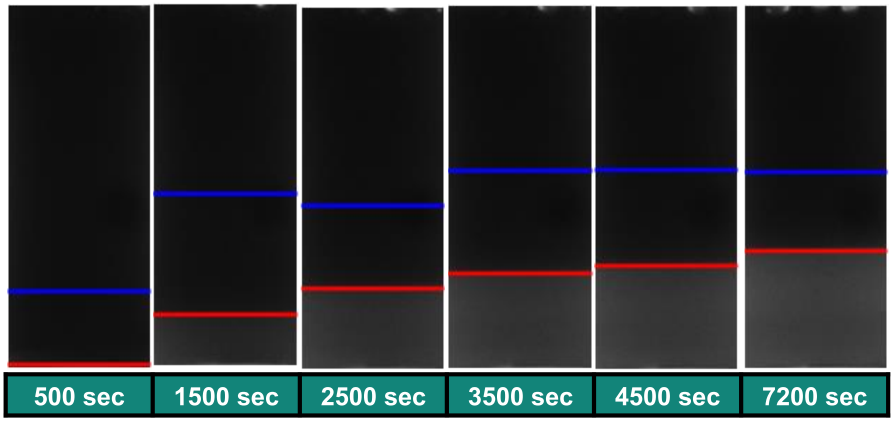

Figure S130: Sample images from 0.1M SDBS solution (1.5907g/100ml NaCl) with location of detected interfaces.

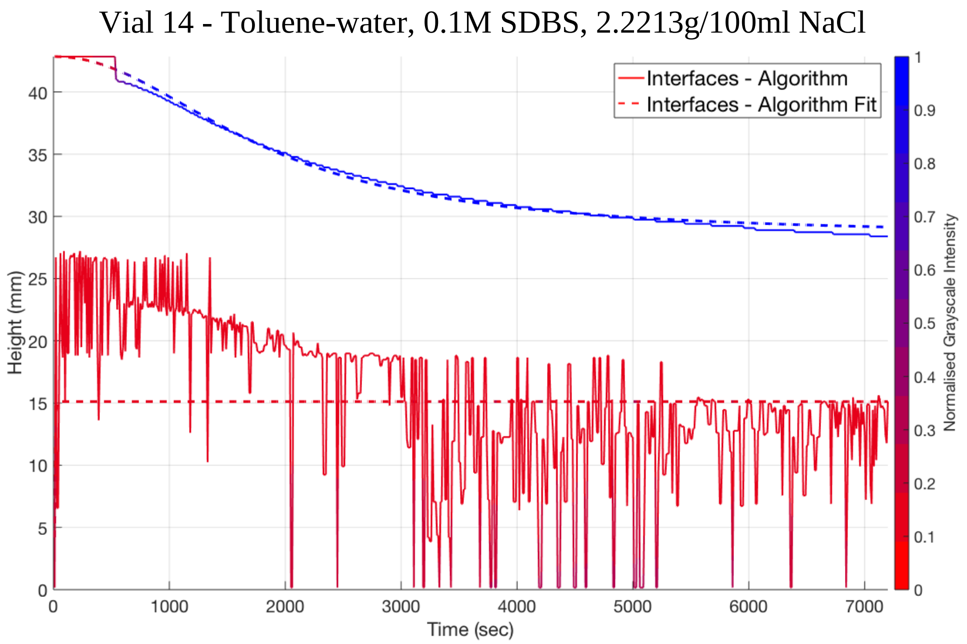

Figure S131: Detected interfaces and normalised grayscale intensity over time of the 0.1M SDBS solution (2.2213g/100ml NaCl).

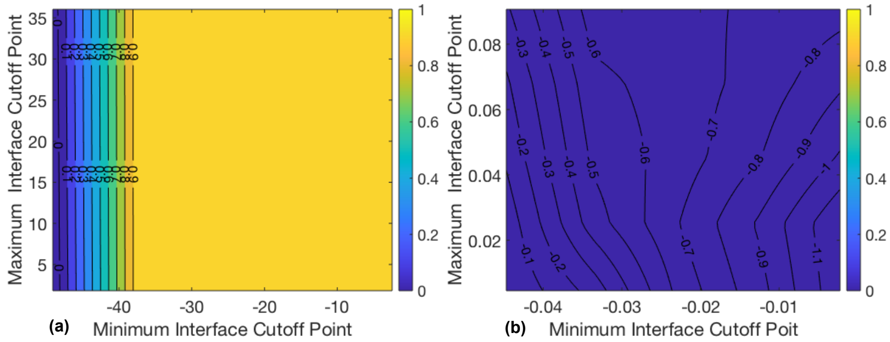

Figure S132: Contour plot of r^2 values for the sigmoidal curve fit for interface 1 data (a) and interface 2 data (b) depending on the maximum and minimum cut-off point combination – 0.1M SDBS solution (2.2213g/100ml NaCl).

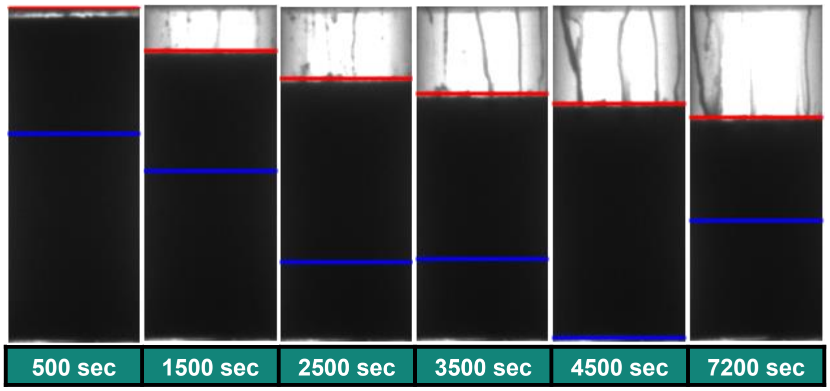

Figure S133: Sample images from 0.1M SDBS solution (2.2213g/100ml NaCl) with location of detected interfaces.

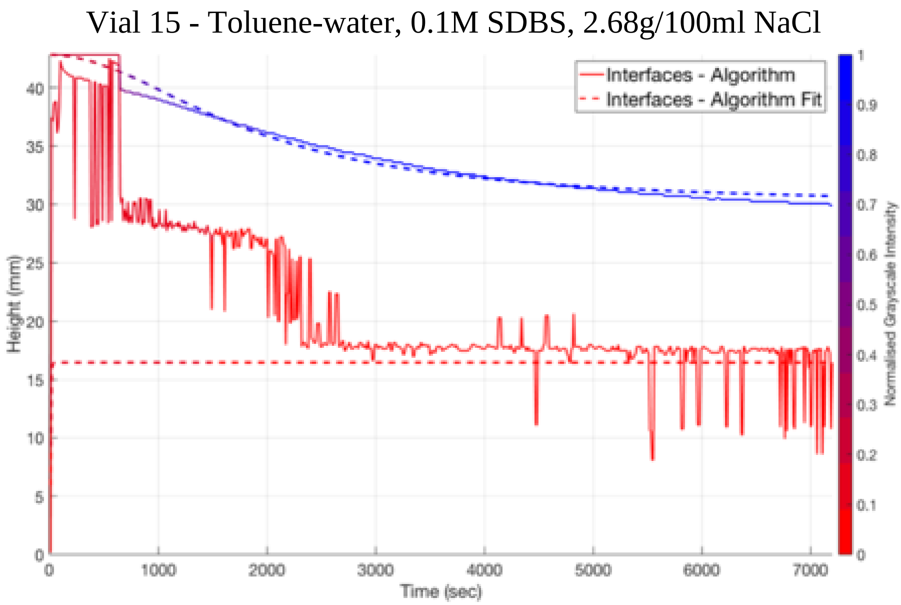

Figure S134: Detected interfaces and normalised grayscale intensity over time of the 0.1M SDBS solution (2.68g/100ml NaCl).

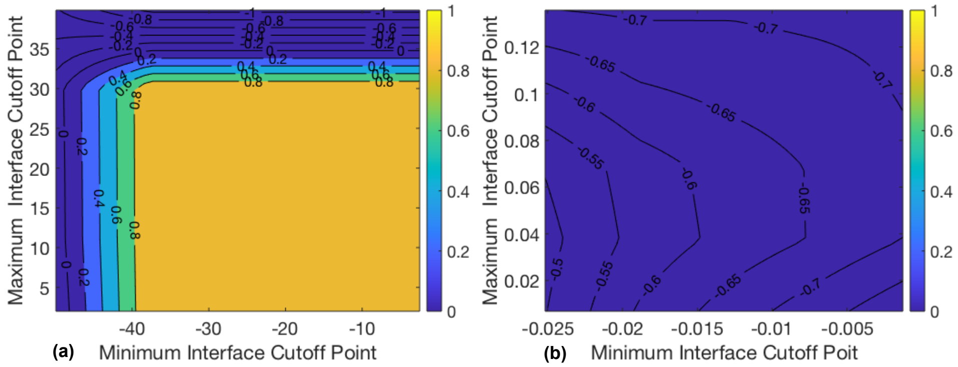

Figure S135: Contour plot of r^2 values for the sigmoidal curve fit for interface 1 data (a) and interface 2 data (b) depending on the maximum and minimum cut-off point combination – 0.1M SDBS solution (2.68g/100ml NaCl).

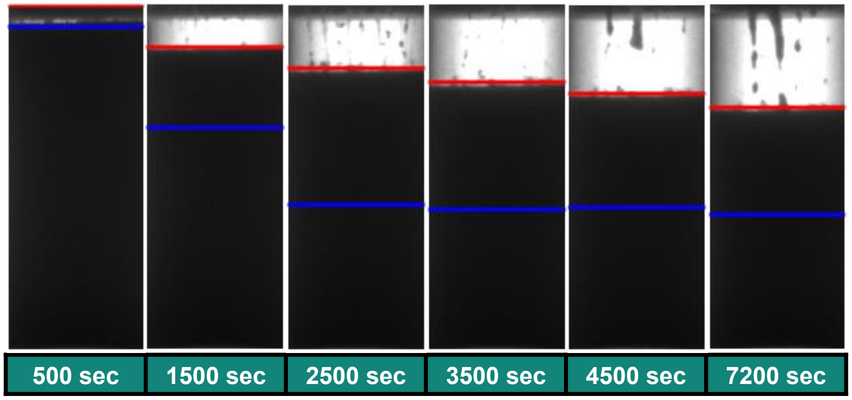

Figure S136: Sample images from 0.1M SDBS solution (2.68g/100ml NaCl) with location of detected interfaces.

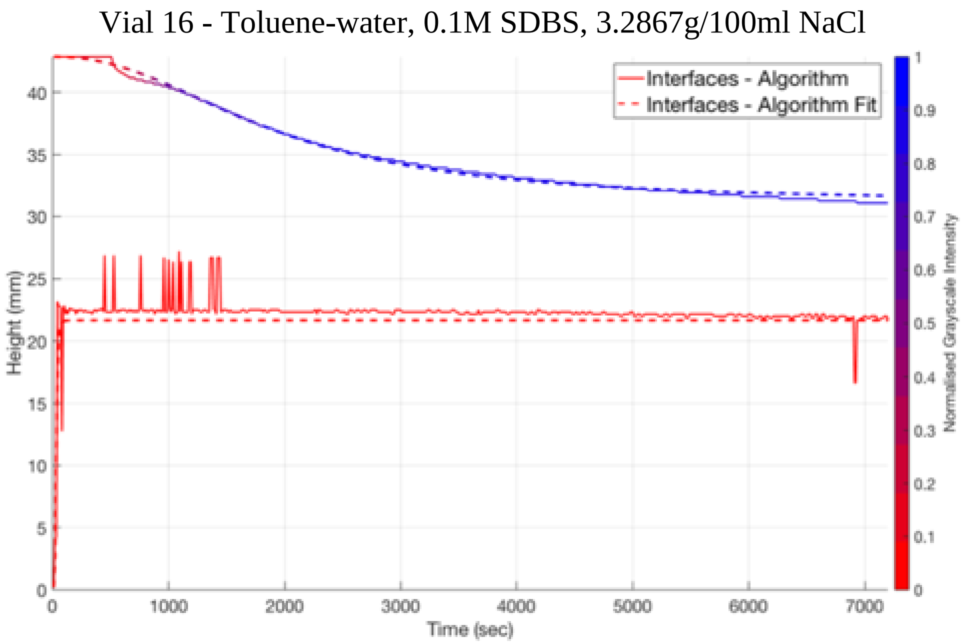

Figure S137: Detected interfaces and normalised grayscale intensity over time of the 0.1M SDBS solution (3.2867g/100ml NaCl).

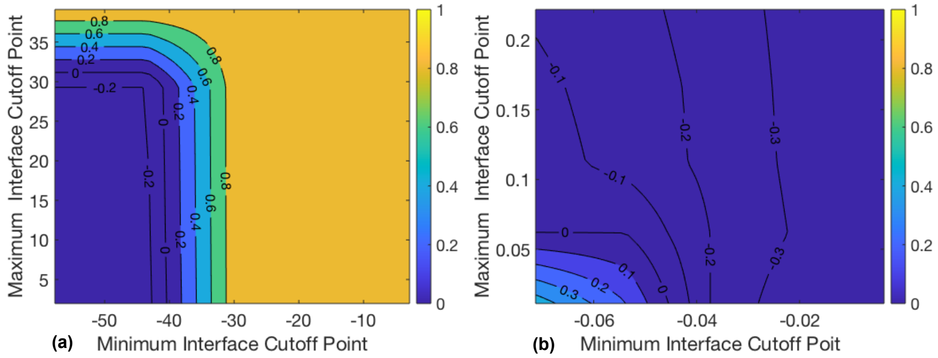

Figure S138: Contour plot of r^2 values for the sigmoidal curve fit for interface 1 data (a) and interface 2 data (b) depending on the maximum and minimum cut-off point combination – 0.1M SDBS solution (3.2867g/100ml NaCl).

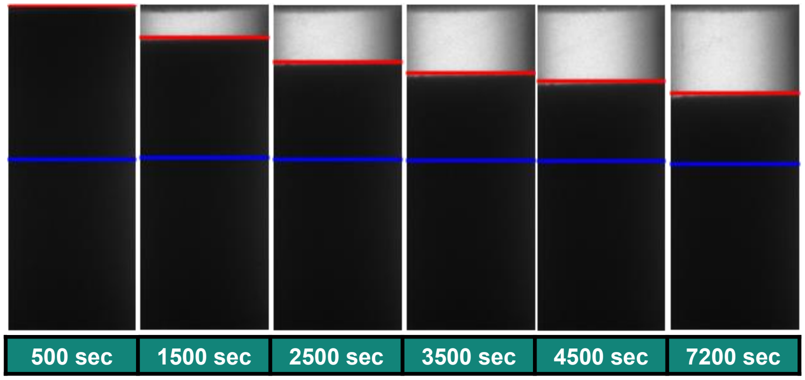

Figure S139: Sample images from 0.1M SDBS solution (3.2867g/100ml NaCl) with location of detected interfaces.

Vial 17 - Toluene-water, 0.1M SDBS, 4.348g/100ml NaCl

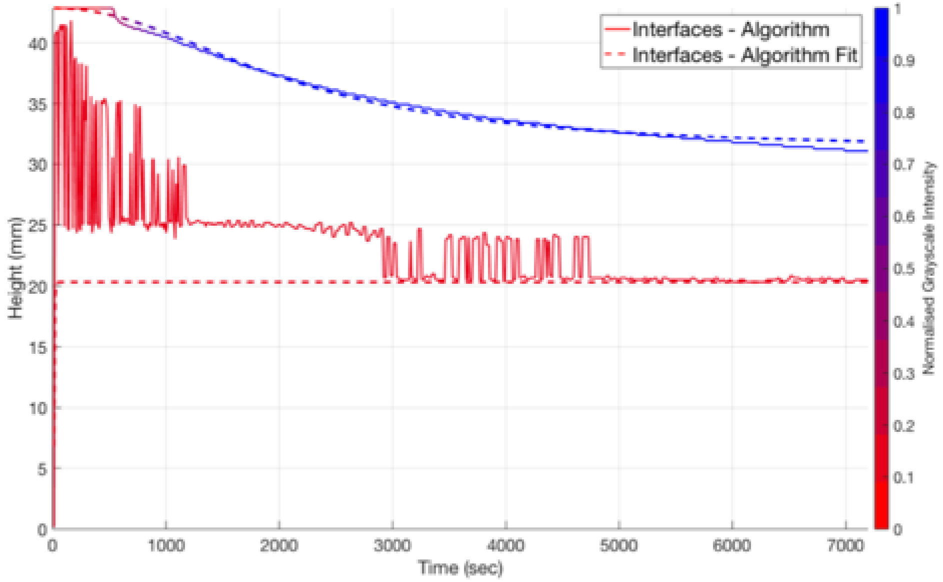

Figure S140: Detected interfaces and normalised grayscale intensity over time of the 0.1M SDBS solution (4.348g/100ml NaCl).

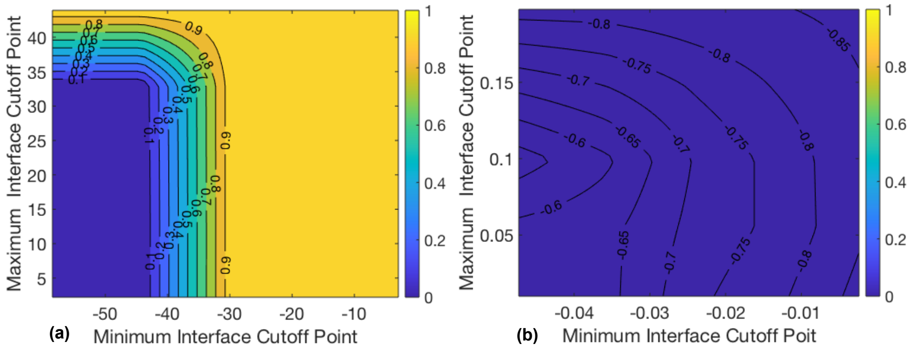

Figure S141: Contour plot of r^2 values for the sigmoidal curve fit for interface 1 data (a) and interface 2 data (b) depending on the maximum and minimum cut-off point combination – 0.1M SDBS solution (4.348g/100ml NaCl).

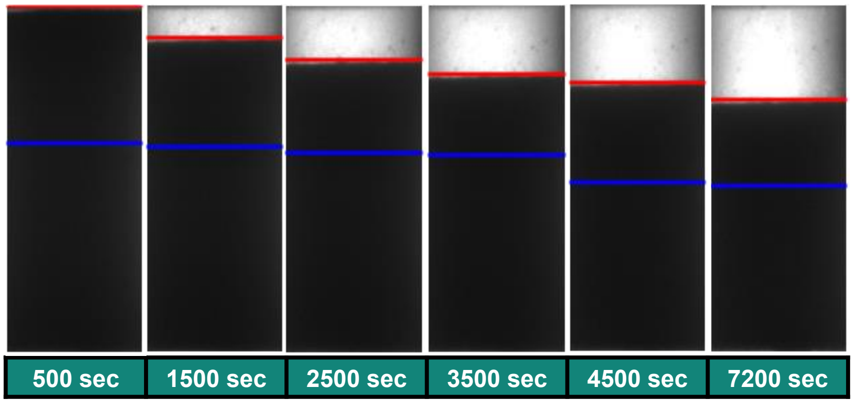

Figure S142: Sample images from 0.1M SDBS solution (4.348g/100ml NaCl) with location of detected interfaces.

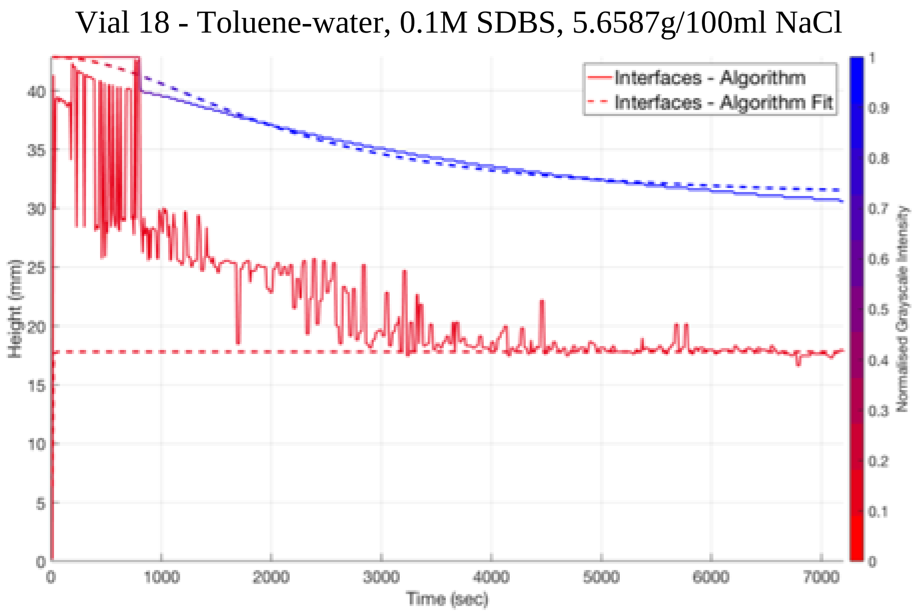

Figure S143: Detected interfaces and normalised grayscale intensity over time of the 0.1M SDBS solution (5.6587g/100ml NaCl).

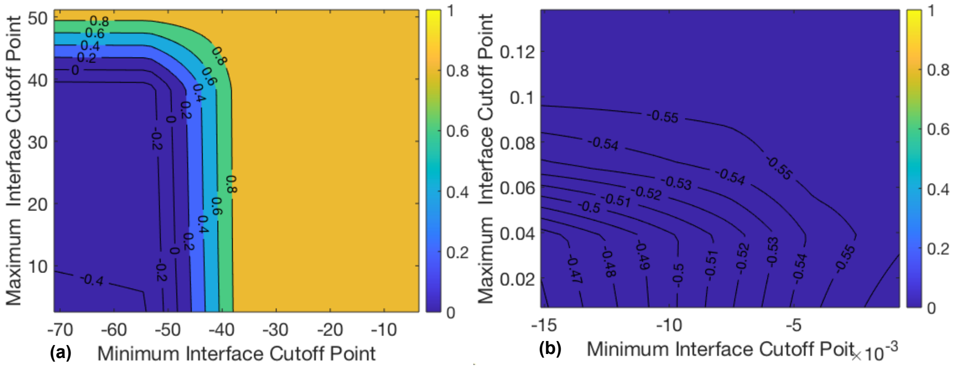

Figure S144: Contour plot of r^2 values for the sigmoidal curve fit for interface 1 data (a) and interface 2 data (b) depending on the maximum and minimum cut-off point combination – 0.1M SDBS solution (5.6587g/100ml NaCl).

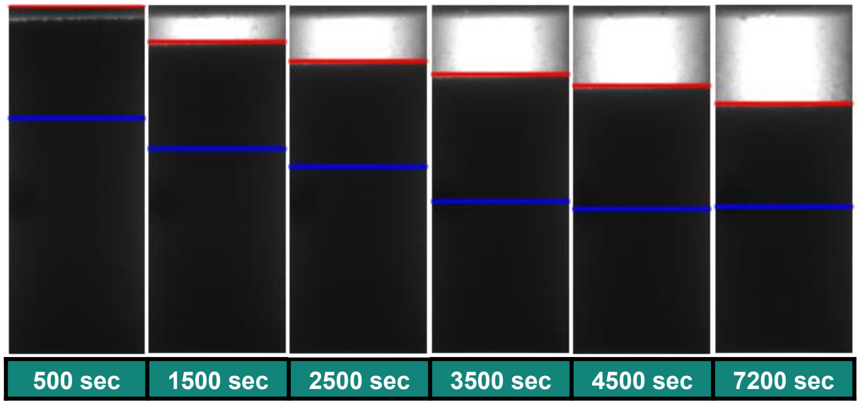

Figure S145: Sample images from 0.1M SDBS solution (5.6587g/100ml NaCl) with location of detected interfaces.

Vial 19 - Toluene-water, 0.1M SDBS, 6.6507g/100ml NaCl

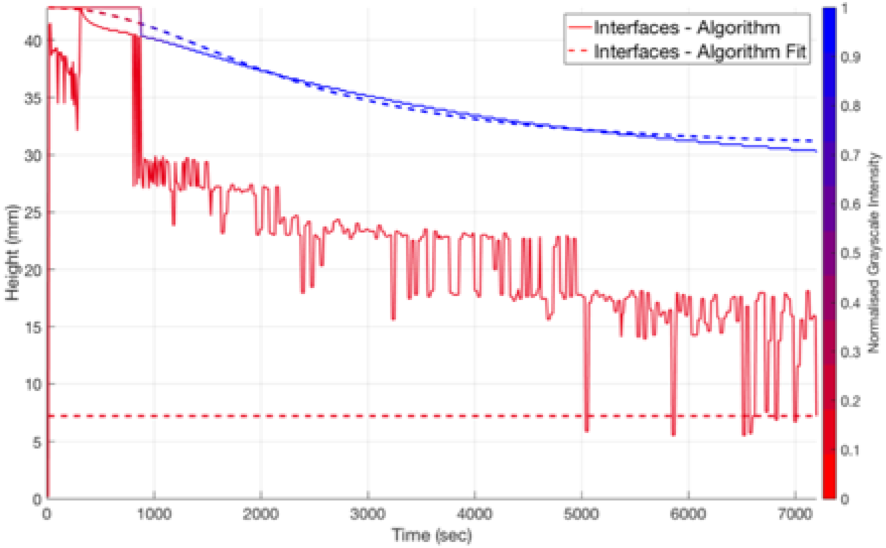

Figure S146: Detected interfaces and normalised grayscale intensity over time of the 0.1M SDBS solution (6.6507g/100ml NaCl).

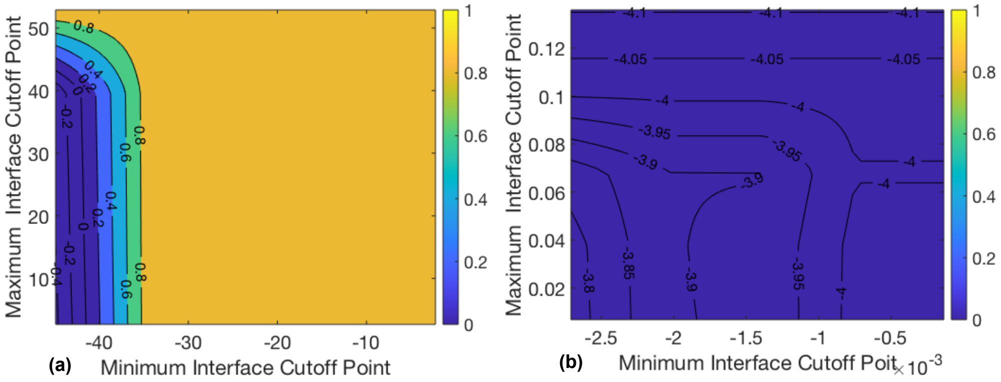

Figure S147: Contour plot of r^2 values for the sigmoidal curve fit for interface 1 data (a) and interface 2 data (b) depending on the maximum and minimum cut-off point combination – 0.1M SDBS solution (6.6507g/100ml NaCl).

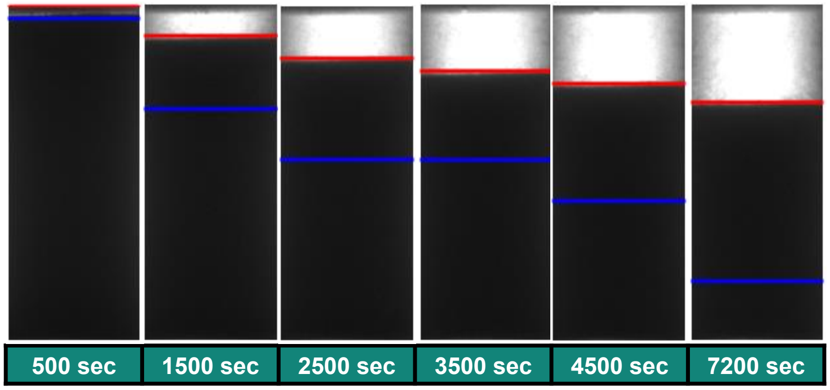

Figure S148: Sample images from 0.1M SDBS solution (6.6507g/100ml NaCl) with location of detected interfaces.

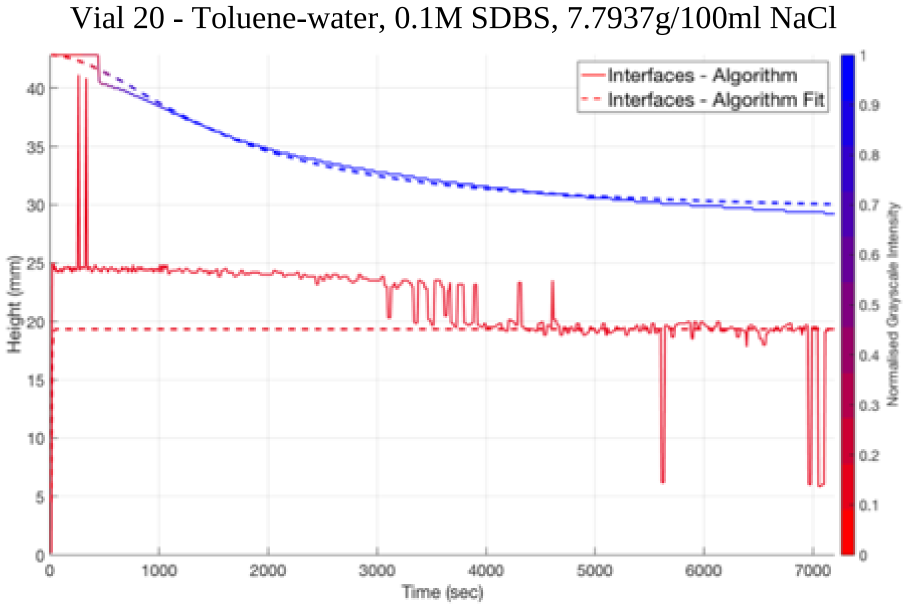

Figure S149: Detected interfaces and normalised grayscale intensity over time of the 0.1M SDBS solution (7.7937g/100ml NaCl).

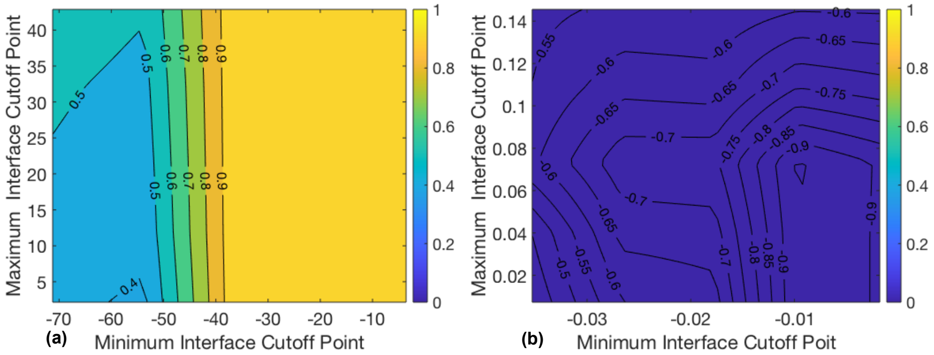

Figure S150: Contour plot of r^2 values for the sigmoidal curve fit for interface 1 data (a) and interface 2 data (b) depending on the maximum and minimum cut-off point combination – 0.1M SDBS solution (7.7937g/100ml NaCl).

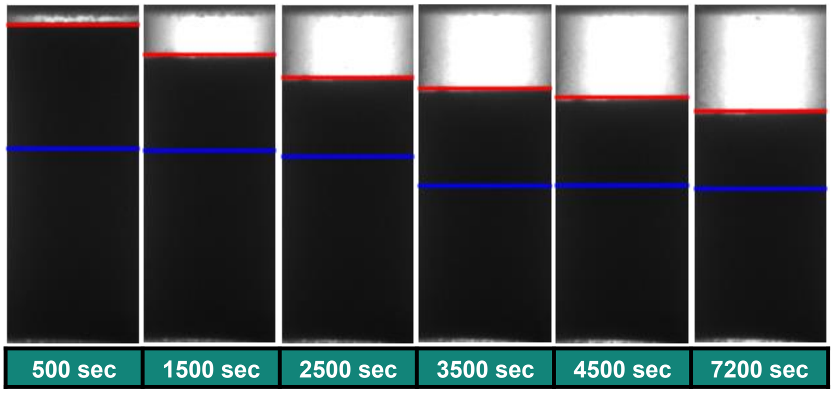

Figure S151: Sample images from 0.1M SDBS solution (7.7937g/100ml NaCl) with location of detected interfaces.

4.3 Scale up experiments

Table S11: Non-surfactant solution times to reach 90% of their final height at 0.2 litre scale and 20 litre scale.

Volume (L)	Height (mm)	Interface	Separation time (sec)	
			Deionised water	Glycine buffer
0.2	66	**Top**	24.6	59.33
		**Bottom**	6.33	148
20	270	**Top**	36	44.5
		**Bottom**	45.5	69

Table S12: Surfactant solution times to reach 90% of their final height at 0.2 litre scale and 20 litre scale.

Volume (L)	Height (mm)	Interface	Separation time (sec)		
			HLD = -0.49	HLD = 0	HLD = 0.45
0.012	43	Top	7200	140	60
		Bottom	7200	2090	450
20	270	Top	9000	51	59
		Bottom	9000	136	72

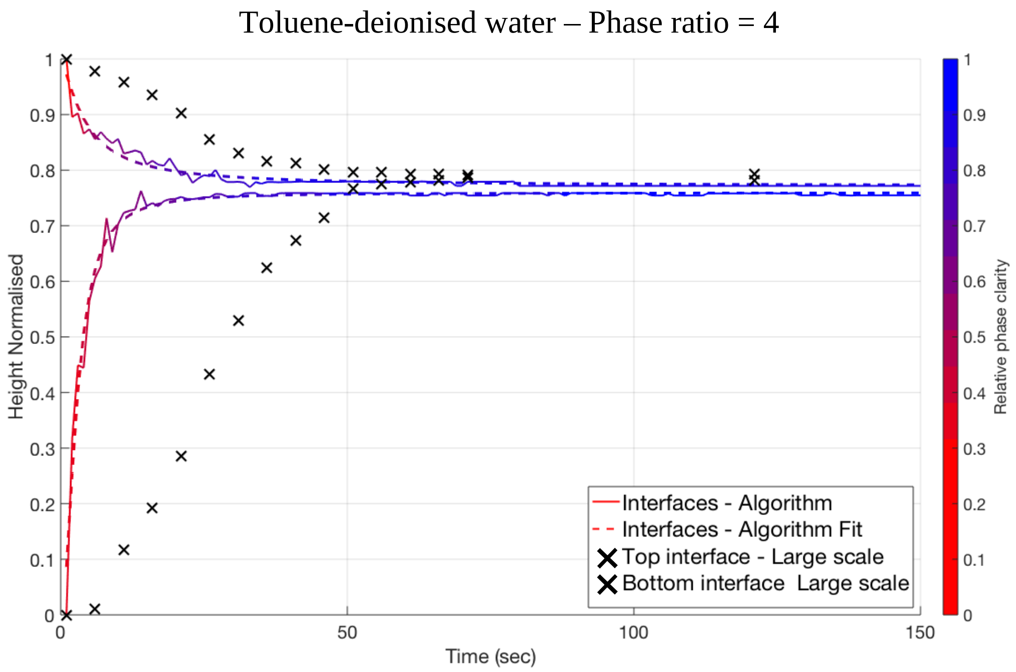

Figure S152: Normalised Height of interfaces over time of the toluene-deionised water case at a phase ratio of 4 at 20 litre scale and small scale.

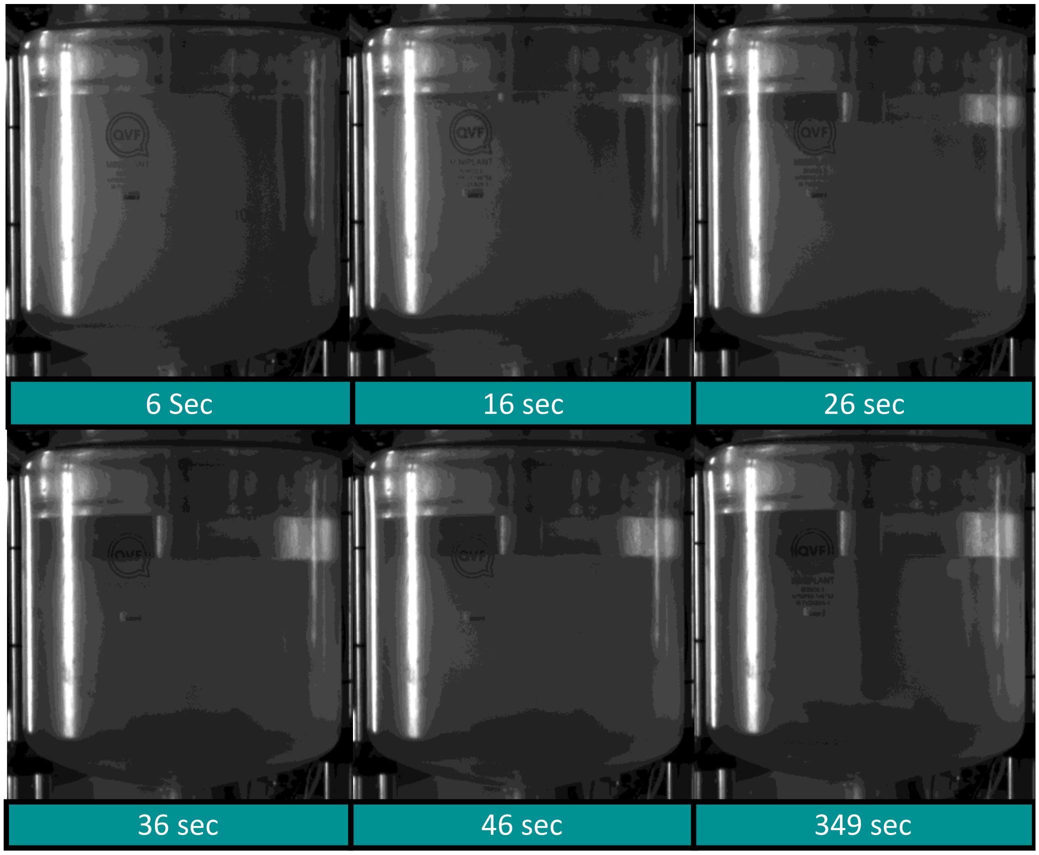

Figure S153: Sample images from toluene-deionised water, phase ratio = 4, 20 litre separation

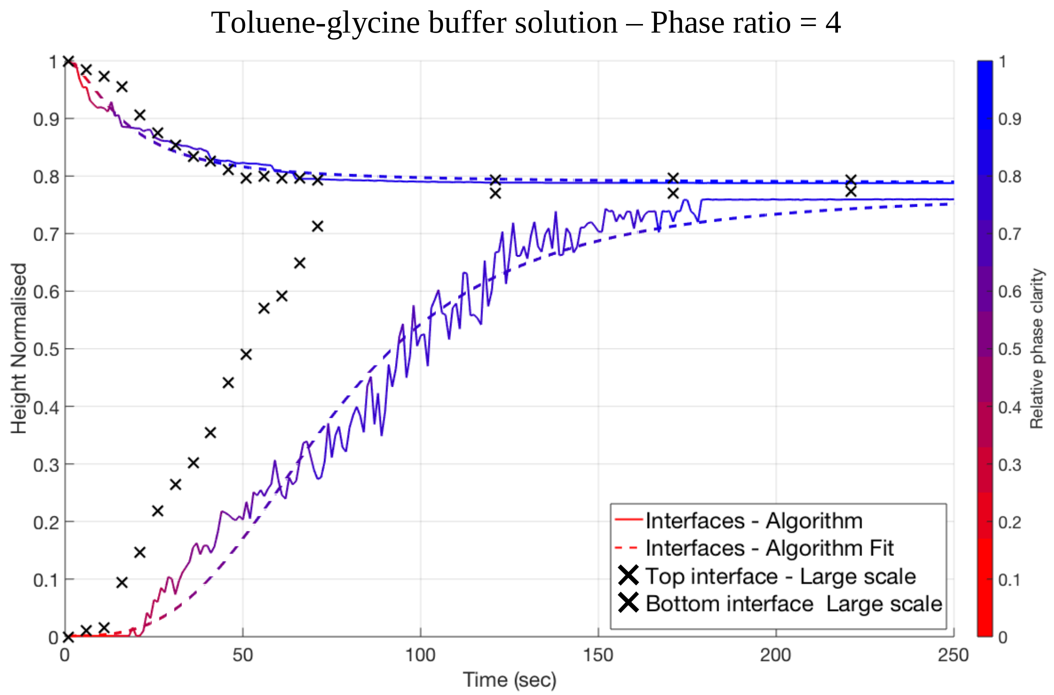

Figure S154: Normalised Height of interfaces over time of the toluene-glycine buffer solution at a phase ratio of 4 at 20 litre scale and small scale.

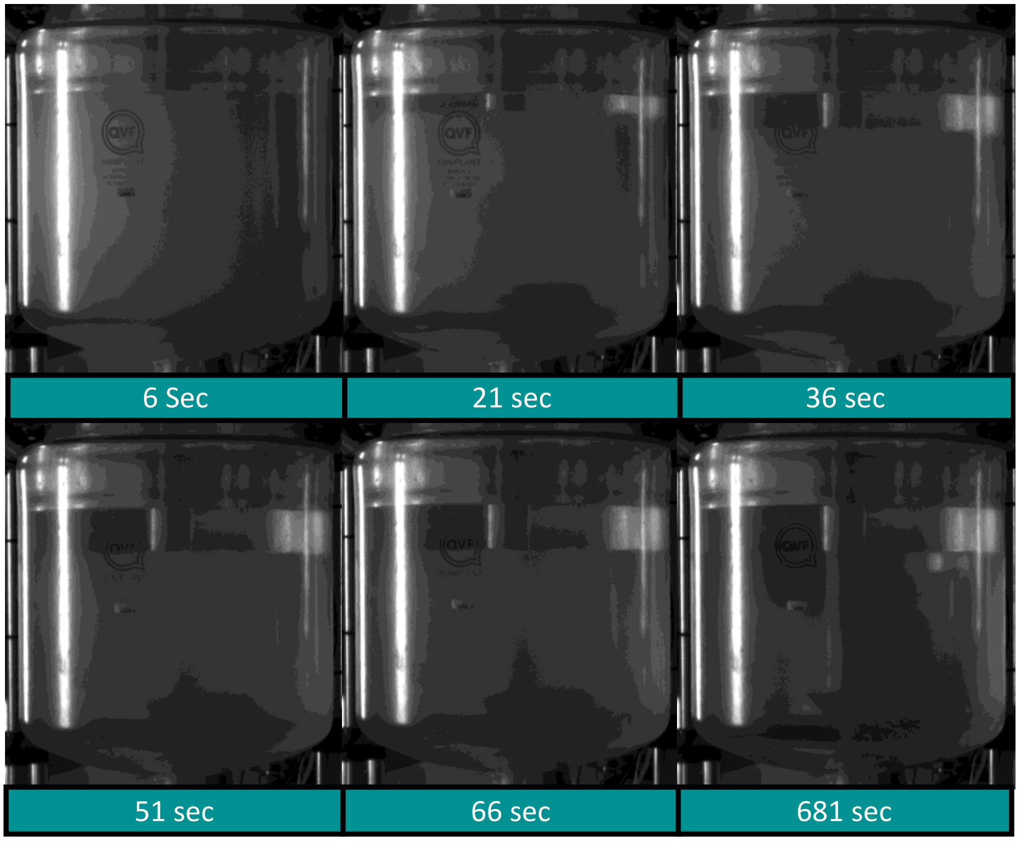

Figure S155: Sample images from toluene-glycine buffer solution, phase ratio = 4, 20 litre separation.

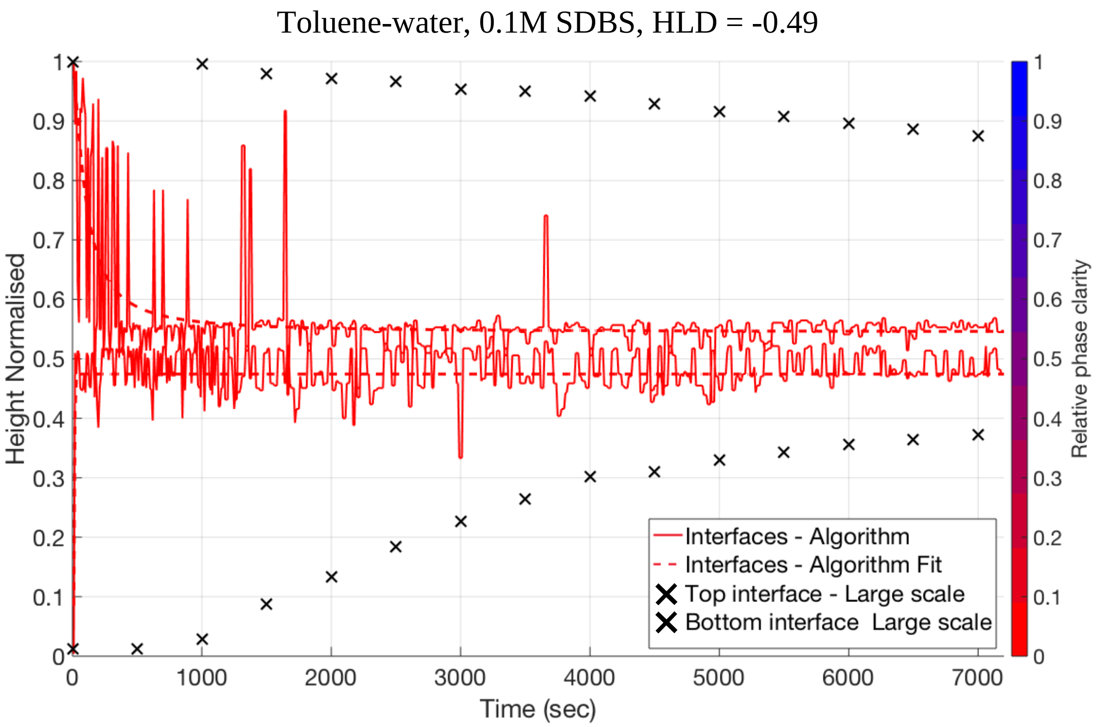

Figure S156: Normalised Height of interfaces over time of the toluene-SDBS surfactant solution, HLD = -0.49 at a phase ratio of 1 at 20 litre scale and small scale.

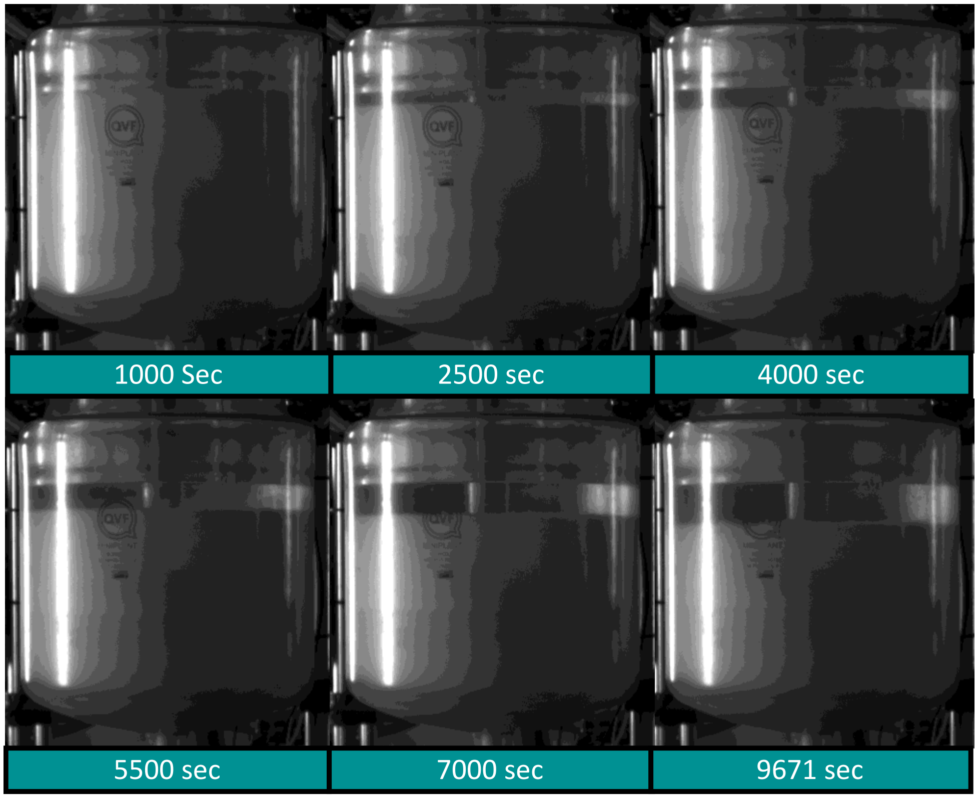

Figure S157: Sample images from toluene-SDBS surfactant solution, HLD = -0.49, phase ratio = 1, 20 litre separation.

Toluene-water, 0.1M SDBS, HLD = 0.006

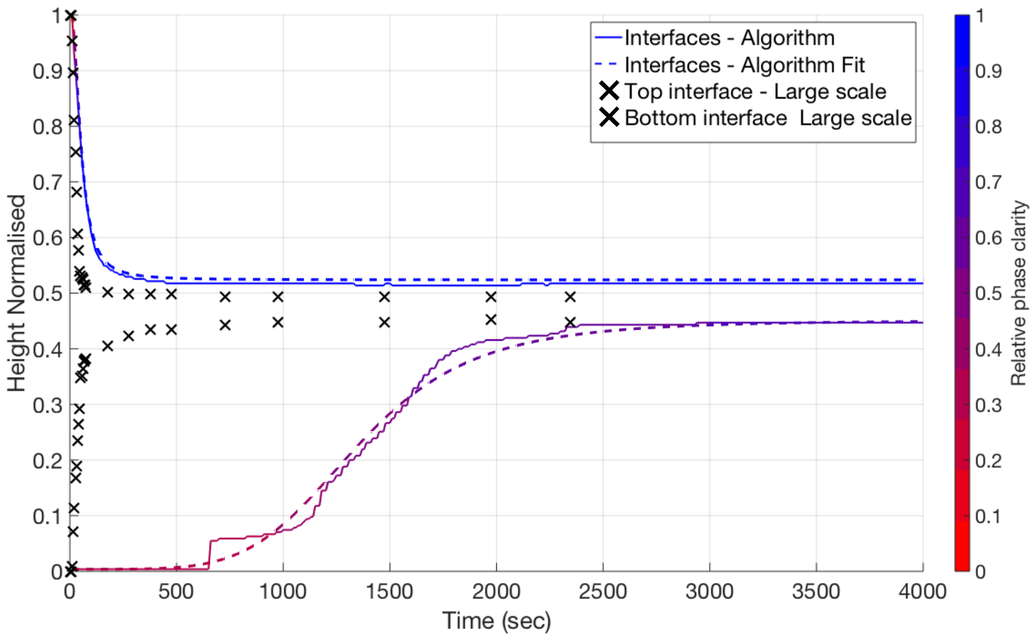

Figure S158: Normalised Height of interfaces over time of the toluene-SDBS surfactant solution, HLD = 0.006 at a phase ratio of 1 at 20 litre scale and small scale.

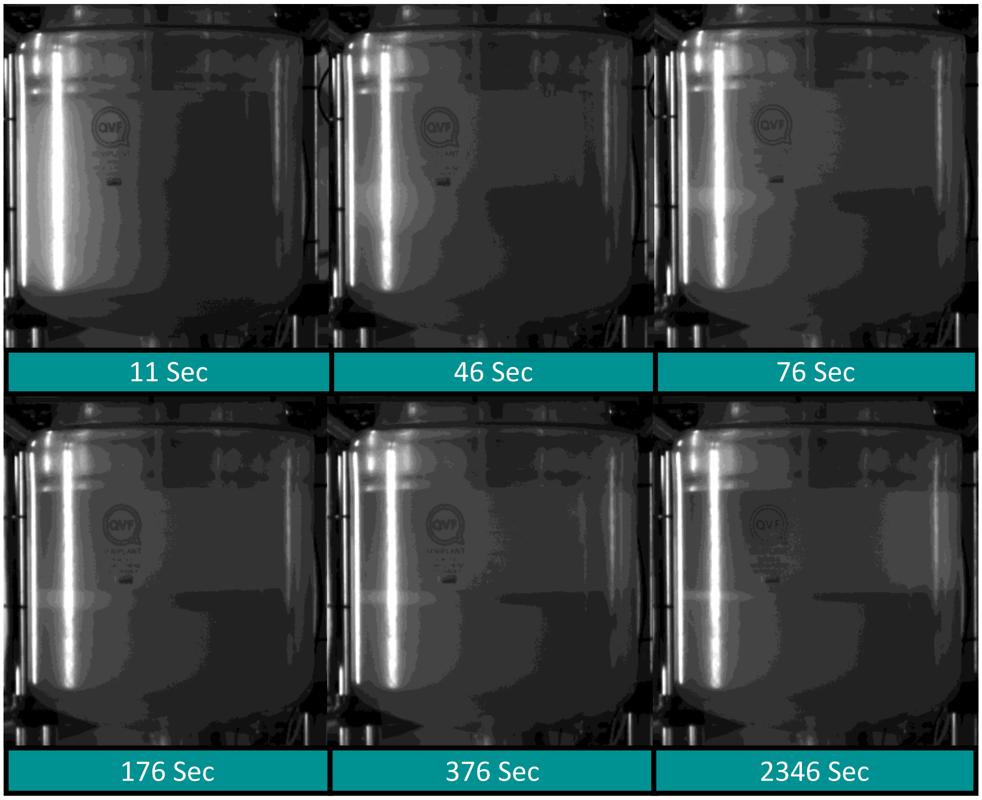

Figure S159: Sample images from toluene-SDBS surfactant solution, HLD = 0.006, phase ratio = 1, 20 litre separation.

Toluene-water, 0.1M SDBS, HLD = 0.45

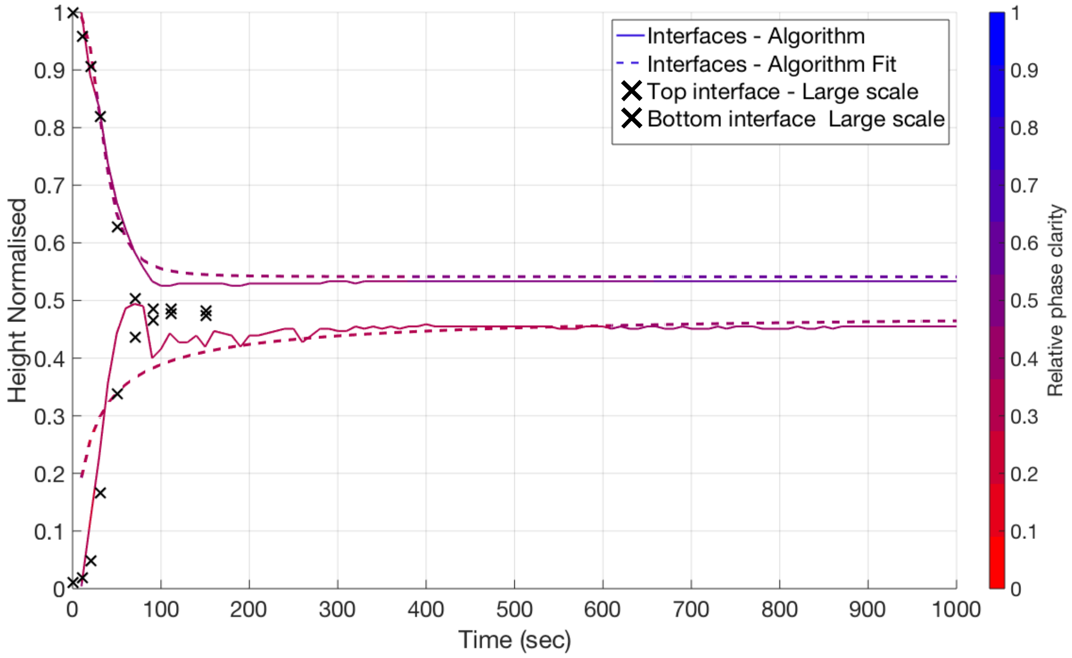

Figure S160: Normalised Height of interfaces over time of the toluene-SDBS surfactant solution, HLD = 0.45 at a phase ratio of 1 at 20 litre scale and small scale.

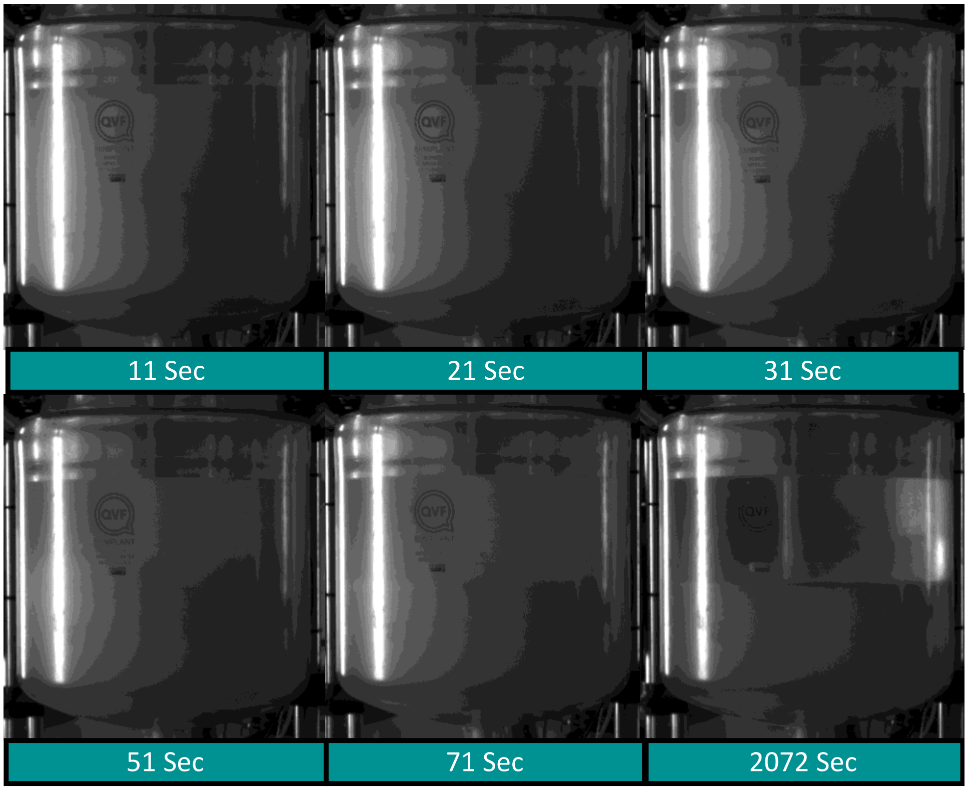

Figure S161: Sample images from toluene-SDBS surfactant solution, HLD = 0.45, phase ratio = 1, 20 litre separation.

5.0 Experimental rig development

During development of the interface detection algorithm an initial prototype of an automatic shaker rig was developed. The shaker would consist of a rack which could hold 8-12 samples in place and be shook for a period of time using a motor and suitable control system. The shaker rack could be stacked to multiply the number of samples that can be analysed at once by n – number of stacks. As an initial proof of concept a single vial containing sunflower oil and water was shook using an Arduino and stepper motor at up to 362 rpm. Figure S161 shows the level of emulsification in the vial at various rpms. The successful emulsification of sunflower oil and water shows that the shaker provides enough mixing energy to successfully and consistently emulsify a liquid-liquid mixture.

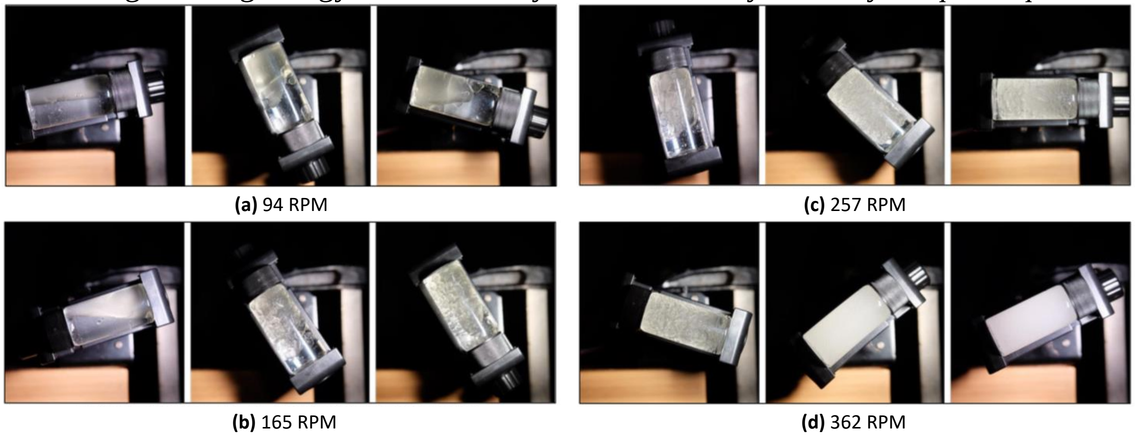

Figure S162: Vial shaker prototype demonstration at various RPM's. Each image at each RPM is taken 30 seconds apart.

6.0 A summary of the HLD method (Abbott, 2017)

$$\mathrm{HLD} = \mathrm{Cc} - \mathrm{k.EACN} - \alpha.(\mathrm{T} - 25) + \mathrm{f(S)}$$

Cc is the surfactant 'characteristic' value that relates to the hydrophobic/hydrophilic nature of the surfactant molecule, k is a constant (0.17), EACN is the Effective Alkane Carbon Number and α is a constant (0.01 for ionic surfactants). T is the temperature of the emulsion in °C and f(S) is a function of salinity that depends on the type of surfactant. For ionic surfactants f(S) = ln(S+SurfSal) where S is the salinity in g/100ml and SurfSal is the salinity contributed by the surfactant in g/100ml NaCl. When HLD = 0 the lowest interfacial tension of the system has been reached. A type III or IV microemulsion can form if the surfactant is dissolved in a sufficiently high concentration. A positive HLD value corresponds to a W/O emulsion and a negative HLD value results in an O/W emulsion. At HLD = 0, separation happens rapidly due to the extremely low interfacial tensions which result in very little resistance to coalescence. Reduced mechanical agitation is required to produce smaller droplets closer to this phase inversion point. The time required for emulsion phases to separate increases exponentially as the HLD value deviates from 0. Only when a system is situated far from the phase inversion point does the time for separation tend to reduce again, due to increased interfacial tension, larger droplet sizes and less efficient surfactant interfacial packing. This characterisation method can be used to understand a given liquid-liquid work-up and establish whether it is likely to result in long separation times or rag layer formation.

Reference: Abbott SJ. Surfactant science: principles & practice. DEStech Publications, Incorporated; 2017.
